# Post-polymerisation oxyfunctionalisation of styrene and butadiene-based (co-)polymers using a homogeneous manganese catalyst†

**DOI:** 10.1039/d5fd00093a

**Published:** 2025-07-11

**Authors:** Maartje Otten, Jeroen Hendriks, Nino Kalános, Arnaud Thevenon, Pieter C. A. Bruijnincx

**Affiliations:** a Organic Chemistry & Catalysis, Institute for Sustainable and Circular Chemistry, Faculty of Science, Utrecht University Universiteitsweg 99, 3584 CG Utrecht The Netherlands a.a.thevenon-kozub@uu.nl p.c.a.bruijnincx@uu.nl

## Abstract

Post-polymerisation modification of commodity hydrocarbon-based polymers provides access to functional polymers not readily available through bottom-up synthesis methods. Here, we demonstrate the oxyfunctionalisation of different styrenic and rubbery (co-)polymers using a well-established and robust manganese-based homogeneous catalyst, MnTACN, a 1,4,7-trimethyl-1,4,7-triazacyclononane ligand-bearing di-nuclear tri-μ-oxo bridged Mn(iv) compound, and hydrogen peroxide as a green oxidant. Using various grades of polystyrene (PS) and polybutadiene (PBD), we successfully oxyfunctionalised the polymer backbones with alcohol (PS and PBD), ketone (PS) and epoxide (PBD) functional groups. Under optimised conditions, total functionalisation degrees up to 5% for PS and 18% for PBD can be achieved. Next to the homopolymers, we also show oxyfunctionalisation degrees as high as 11%, of the butadiene-derived part of a styrene–butadiene–styrene block-co-polymer (SBS). These results underscore the versatility of a single catalytic system for the oxyfunctionalisation of various C–H bonds as well as the C=C bonds found in these commodity hydrocarbon polymers. Detailed analysis of the oxidised polymers before and after subsequent oxidative cleavage of the installed diol moieties on the PBD backbone suggest that the functional groups are randomly spaced along the polymer backbone. Moreover, this second oxidative cleavage also offers the possibility to selectively break down the polymer backbone after oxyfunctionalisation into a mixture of dialdehyde oligomers consisting of 4 up to 32 monomeric units. For PBD and low/mid *M*_w_ PS, oxyfunctionalisation coincided with minimal backbone cleavage or crosslinking, as evidenced by gel permeation chromatography (GPC). For the high molecular weight PS samples and SBS, GPC analysis suggests that backbone cleavage is in contrast more pronounced upon oxyfunctionalisation. The thermal properties of the oxyfunctionalised materials are largely unchanged, with decomposition temperatures decreasing with increasing functionalisation degrees, but overall remaining in the high thermal stability regime.

## Abstract 2

We demonstrate the oxyfunctionalisation of styrenic and rubbery (co-)polymers using a manganese-based homogeneous catalyst, MnTACN, a 1,4,7-trimethyl-1,4,7-triazacyclononane ligand and hydrogen peroxide as a green oxidant.

## Graphical abstract

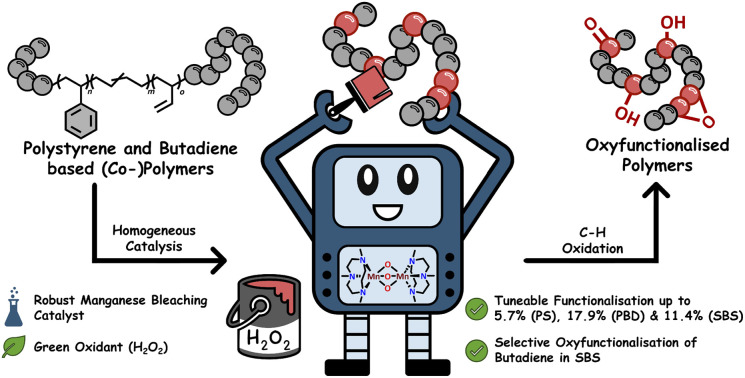

## Introduction

Commodity polymers made from hydrocarbons such as polystyrene (PS) and polybutadiene (PBD) have highly attractive properties, including their durability, being lightweight, and high processability.^1,2^ As a result, these styrenic and rubbery (co-)polymers are omnipresent in our daily lives. A common strategy to tune their bulk properties is to co-polymerise them with a co-monomer bearing a desired functionality. This is industrially applied *e.g.* in the production of copolymers of PS and PBD where the high flexibility of the PBD homopolymer is combined with the high tensile strength of the PS homopolymer. Combining the properties of the homopolymers results in co-polymers with extended property space, of which high impact polystyrene (HIPS) and styrene–butadiene–styrene (SBS) block-co-polymers are very prominent examples.^3^ While inclusion of more polar co-monomers is also desirable, such as in acrylonitrile–butadiene–styrene (ABS) co-polymers, the range of polar monomers suitable for co-polymerisation is limited. In particular, monomers bearing oxyfunctional groups are typically not compatible with the current polymerisation strategies.^4–8^ This is clearly an undesirable restriction on the chemical space accessible through bottom-up synthesis methods for styrenic and rubbery polymers. Alternative approaches to bottom-up instalment of polar functional groups to tailor the polymer properties, *i.e.* increase their compatibility in polymer blends or to install handles to promote controlled degradation for end-of-life, are therefore highly sought after. The nature of and degree to which the polar functional groups can be installed is a crucial parameter to change, control and fine-tune the bulk properties of polymers such as PS or PBD.^9^

**Scheme 1:**
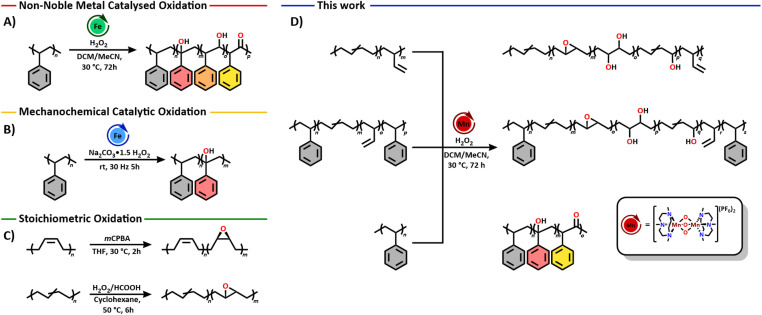
(A) Non-noble metal catalysed oxidation of polystyrene.^16^ (B) Solvent-free mechanochemical catalytic oxidation of polystyrene.^18^ (C) Stoichiometric oxidation of polybutadiene by mCPBA and H_2_O_2_/formic acid.^20,22^ (D) This work on the oxidation of polystyrene and polybutadiene using a robust manganese oxidation catalyst.

Post-polymerisation modification (PPM) has emerged as a versatile late-stage strategy for expanding polymer chemical space.^10^ PPM has shown to be a powerful tool to oxyfunctionalise different types of hydrocarbon-based polymer backbones in a well behaved manner and enhance the polymeric properties without loss of the original desirable bulk properties of the starting polymer.^11–17^ For PS and PBD, homogeneously catalysed oxyfunctionalisation approaches are scarce, however. One of the few examples known for oxyfunctionalisation of PS is reported by McArthur and Baird using an iron *N*,*N*′-dimethyl-*N*,*N*′-bis(2-pyridylmethyl)-ethane-1,2-diamine system (Scheme 1A).^16^ This catalytic system showed controlled oxyfunctionalisation of low molecular weight PS (*M*_w_ = 1.0 kDa), installing 2° and 3° alcohol functional groups. Some of the secondary alcohols were subsequently oxidized to the corresponding ketones. In contrast, when PS with a high *M*_w_ (120 kDa) was used during the reaction, significant backbone crosslinking and cleavage (to a *M*_w_ of 26 kDa) was observed. More recently, Gupta and Sivaram demonstrated the solvent-free mechanochemical oxidation of low *M*_w_ models of PS and PP using an iron-tetra-amido macrocyclic ligand based catalyst.^18^ While reactivity was promising on low *M*_w_ polymer models, more industrially relevant high molecular weight polymer samples were not included (Scheme 1B). The oxidation of unsaturated bonds, such as those in PBD, has been explored with heterogeneous catalysts as well as with stoichiometric oxidants such as *m*-chloroperbenzoic acid (*m*CPBA) or hydrogen peroxide (H_2_O_2_)/formic acid (Scheme 1C).^19,20^ While for both approaches high epoxidation degrees of 5% up to 35% were achieved, no information was reported on any molecular weight changes after oxyfunctionalisation. Examples on oxidation of the unsaturated bonds in the backbone using homogeneous catalytic systems are scarce, and do not report on possible molecular weight changes either.^21^

Herein, we aimed to look into a robust and well-defined homogeneous catalyst with a first-row transition metal capable of oxidising both aliphatic C–H and unsaturated bonds in (un)saturated polyolefins. A catalytic system well known for such oxidative activity is the manganese-1,4,7-trimethyl-1,4,7-triazacyclononan (Mn(TACN)) catalyst which has been developed by Wieghardt *et al.*, as a biomimetic model of manganese-containing enzymes.^23^ The Mn(TACN) catalytic system was thereafter extensively studied by Hage and co-workers, with H_2_O_2_ as the oxidant, patented by Unilever, and is nowadays still used in dishwasher tablets.^24–26^ The catalyst has shown to be highly active towards the oxidation of both small molecule alkanes such as cyclohexane and adamantane as well as alkenes such as styrene, cyclohexene and norbornene.^26,27^ This high oxidative activity makes this catalyst a highly interesting candidate for selective polymer functionalisation. Furthermore, the use of one catalyst for the oxidation of different types of C–H bonds in (un)saturated polyolefins, has not been reported up to now and is highly interesting when looking at the oxyfunctionalisation of co-polymers, such as SBS, and chemo-selective functionalisation of mixed plastic wastes. Accordingly, we report the oxyfunctionalisation of PS, PBD and SBS using Mn(TACN) as a robust catalyst and hydrogen peroxide as a green oxidant (Scheme 1D). We show that alcohols, ketones and epoxides can be readily introduced, in functionalisation degrees up to 18%, after optimisation of the reaction using mild conditions with catalyst loadings of 5.0% for PS and as low as 0.03% for PBD. We demonstrate that the catalyst is active towards both PS and PBD homo- and co-polymers and that the installed groups on the PBD backbone also offer potential for further exploitation in degradation strategies.

## Results and discussion

To develop the oxyfunctionalisation PPM methodology for styrene and butadiene-based polymers, we first looked into a proper solvent system for the reaction. Typically, oxidation reactions catalysed by Mn(TACN) are run in water or acetonitrile (MeCN). However, oxyfunctionalisation does not proceed efficiently in these solvents as the polymers are not soluble and remain as a suspension. Typical solvents used for dissolution of styrenic and butadiene-based polymers are polar, non-protic halogenated solvents such as chloroform, dichloromethane and tetrachloroethane or tetrahydrofuran (THF). Of these, THF is not suitable as a solvent for the targeted oxidation reaction, considering its propensity to form potentially dangerous peroxides. We therefore used a combination of MeCN and dichloromethane (DCM) in a 3 : 7 (v/v) ratio as a suitable single-phase dual-solvent system. It enabled solubilisation of both the polymers and the catalyst, while maintaining the same catalytic activity and selectivity as with the traditional solvents for the oxidation of cyclohexane.

**Fig. 1:**
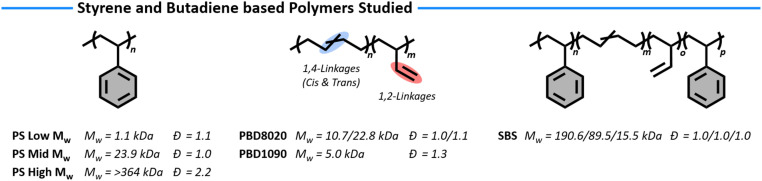
Polystyrene, polybutadiene and styrene–butadiene–styrene block-co-polymers studied in this research with the corresponding molecular weights and dispersity.

With the solvent system being set for the oxidation reactions of the polymeric materials we selected a set of styrene and butadiene-based homo- and co-polymers to investigate the PPM efficiency (Fig. 1). For PS, we explored the functionalisation of two well-defined commercial polymers (polystyrene low molecular weight (PSLMW) *M*_w_ = 1.1 kDa, *Đ* = 1.1 and polystyrene mid molecular weight (PSMMW) *M*_w_ = 23.9 kDa, *Đ* = 1.0) as well as the functionalisation of high molecular weight PS, representative of industrially produced materials (polystyrene high molecular weight (PSHMW), *M*_w_ = >364 kDa, *Đ* = 2.2). To compare to PS, we looked at two different grades of PBD, one containing 80% *cis* and *trans* 1,4-type linkages and 20% vinyl 1,2-type linkages (PBD8020 *M*_w_ = 10.7/22.8 kDa, *Đ* = 1.1/1.0) and the other containing 10% *cis* and *trans* 1,4-type linkages and 90% vinyl 1,2-type linkages (PBD1090 *M*_w_ = 5.0 kDa, *Đ* = 1.3). Last, we used a commercial SBS containing 30 wt% styrene (*M*_w_ = 15.5/89.5/190.6 kDa, *Đ* = 1.0/1.0/1.0) to study the effect of the oxyfunctionalisation on both monomer types present in one co-polymer.

### Oxyfunctionalisation of polystyrene

**Scheme 2:**
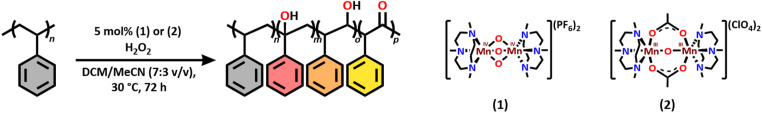
Oxyfunctionalisation of PSHMW using the tri-oxo (1) and mono-oxo-di-acetate (2) bridged analogues of MnTACN.

We started off by exploring the catalytic activity of both the tri-oxo (1) and the mono-oxo-di-acetate (2) bridged analogues of the Mn(TACN) catalyst system (Scheme 2) on PSHMW. Analogue 1 is typically more active towards oxidation, while analogue 2 is more stable.^25^ When testing both analogues, with dropwise addition of the oxidant over the course of 1 h, only trace amounts of oxyfunctionalisation were observed with a 5 mol% catalyst loading of analogue 2, whereas analogue 1 effectively oxyfunctionalised PS under the same reaction conditions. This is in line with previous results demonstrating that the electrophilicity of the Mn(iv) species (1) is much higher than for the Mn(iii) (2), making it a better catalyst for C–H bond activation.^23,28^ Control experiments showed that oxyfunctionalisation of PS does not occur in the absence of either the catalyst or H_2_O_2_, indicating that both are essential for the reaction to proceed.

**Fig. 2:**
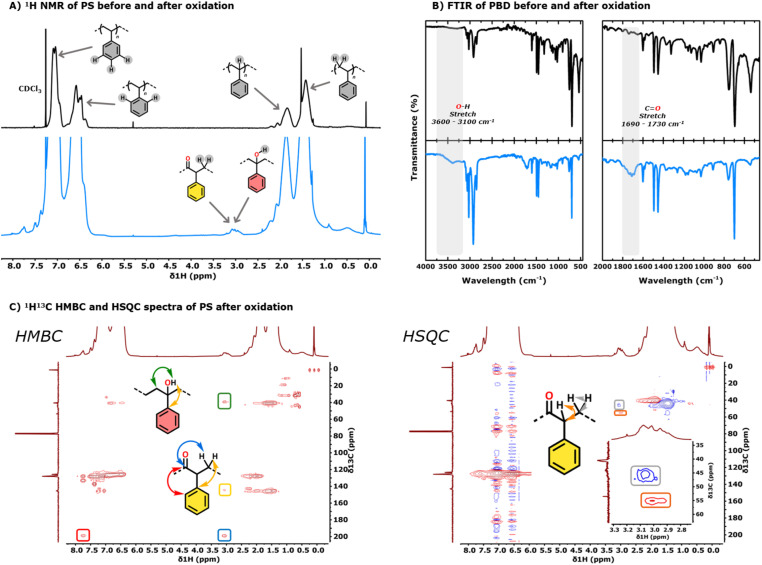
(A) ^1^H NMR spectra of unfunctionalised PS (black trace) and oxyfunctionalised PS (blue trace). (B) FTIR spectra of unfunctionalised PS (black trace) and oxyfunctionalised PS (blue trace). (C) ^1^H^13^C HMBC and HSQC spectra of oxyfunctionalised polystyrene.

Oxyfunctionalisation of the PS backbone is evidenced in the ^1^H NMR spectrum by a new broad resonance at 3.05 ppm, which has previously been assigned to a combination of alcohol functional groups and α-CH_2_ protons adjacent to a carbonyl group (Fig. 2A).^16,18,29^ This assignment is further supported by the FTIR spectrum of the oxidised polymer which shows new vibrations at 3450 cm^−1^, corresponding to the O–H stretch of an alcohol, and at 1715 cm^−1^, corresponding to a C=O stretch of a carbonyl (Fig. 2B). The ^1^H^13^C HMBC spectrum shows that the broad resonance at 3.05 ppm couples only with a secondary carbon adjacent to the alcohol at 39.4 ppm (Fig. 2C), indicating that alcohol functional groups are predominantly located on tertiary carbons (red-highlighted monomeric unit in Scheme 2). While oxyfunctionalisation also occurs at secondary positions (orange-highlighted monomeric unit in Scheme 2), these secondary alcohols are more prone to further oxidation to ketones. This is supported by the ^1^H^13^C HSQC spectrum (Fig. 2C), where the broad resonance at 3.05 ppm correlates with an α-CH carbon at 55.1 ppm and a β-CH_2_ carbon at 45.3 ppm, consistent with a ketone-containing structure (yellow-highlighted monomeric unit in Scheme 2).^18,29^ The absence of new resonances at around 5 ppm in the ^1^H NMR and 155 ppm in the ^13^C NMR, as well as no coupling in the ^1^H^13^C 2D spectra in these regions, suggests that the phenyl rings are not oxidised.^26^

Due to the signal overlap of the alcohol and carbonyl resonances at 3.05 ppm in the ^1^H NMR, quantification of each individual type of functional group on the oxidised PS is challenging. As a result, only a range of the oxyfunctional groups installed can be provided. Integration of the resonance at ∼3.05 ppm shows the presence of 1.8% to 3.7% oxyfunctional groups (FG%, *i.e.* per 100 monomers) after reaction with 5 mol% of catalyst (Table 1, entry 5). Reducing the catalyst loading of (1) to 1 mol%, decreased the degree of oxyfunctionalisation to 1.3–2.9% (Table 1, entry 4), while no oxyfunctionalisation occurred at 0.5 mol% loading (Table 1, entry 3).

**Table 1:** Investigation of the catalytic oxidation conditions on the oxyfunctionalisation of PS materials using the Mn(TACN)-tri-oxo-bridged analogue

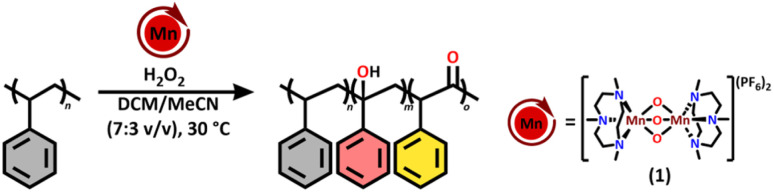						
Entry	Sample	Cat. (mol%)	Portions H_2_O_2_^a^	Reaction time (h)	eFG%	Yield recovered material (%)
1	PSHMW	0	1	72	0	91
2	PSHMW	5	0	72	0	90
3	PSHMW	0.5	1	72	0	98
4	PSHMW	1	1	72	1.3–2.9	84
5	PSHMW	5	1	72	1.8–3.7	88
6^b^	PSHMW	5	3	144	2.9–5.7	86
7^c^	PSHMW	5	10	312	2.8–5.4	85
8^d^	PSHMW	5	2	192	2.3–4.6	87
9	PSMMW	5	1	72	0.7–1.3	87
10^d^	PSLMW	5	2	192	2.2–4.4	86

a In total 6.0 equivalents of H_2_O_2_ were used in respect to the monomeric unit for each experiment.

b 3 × 2.0 equivalents of H_2_O_2_ were added with a 24-hour time interval.

c 10 × 0.6 equivalents of H_2_O_2_ were added with a 24 h time interval.

d 2 × 3.0 equivalents of H_2_O_2_ were added with a 96 h time interval.

e A range for the functionalisation degree (FG%) is presented due to signal overlap in ^1^H NMR, hindering individual quantification of the alcohol and carbonyl.

Then, we looked into the effect of H_2_O_2_ addition methods while keeping the catalyst loading constant at 5 mol% and the total volume of oxidant unchanged. H_2_O_2_ is typically added slowly to the substrate and catalyst to avoid catalyst decomposition, unwanted H_2_O_2_ disproportionation and to thus provide controlled oxidation of the substrate with higher yields.^16,30^ To further slow the addition rate of H_2_O_2_, the total volume was added dropwise in three portions, with 24-hour intervals between each. This slower addition method led to increased oxyfunctionalisation of PS, reaching 2.9–5.7% (Table 1, entry 6 and 7). Addition of even smaller portions (10×) at the same 24-hour intervals (Table 1, entry 7) did not further improve the degree of functionalisation (2.8–5.4%) but did demonstrate the good stability of the catalyst over the prolonged reaction time. A slightly lower FG% of 2.3–4.6% is observed when H_2_O_2_ is added in two larger portions with a 96-h interval (Table 1, entry 8).

The oxyfunctionalisation method proved applicable to PS samples of varying molecular weight. Oxidation of PSMMW resulted in a slightly lower FG% (0.7–1.3%) when the same oxidant ratio was added in a single portion (Table 1, entry 9). In contrast, the oxidation of PSLMW using the portion-wise addition method yielded a similar FG% (2.2–4.4%) to that observed for PSHMW (Table 1, entry 10). The observed FG% is comparable to the solvent-free hydroxylation approach by Sivaram and Gupta, where 4–6% FG% was observed on a low *M*_w_ PS.^18^ The work of the group of Baird demonstrated a higher FG% 10–20% on low *M*_w_ PS; however, backbone cleavage was also observed.^16^

Subsequently, we examined whether the oxyfunctionalised PS (OPS) retained the integrity of its backbone, specifically, whether backbone cleavage or crosslinking had occurred. GPC analysis shows no evidence of major backbone cleavage or crosslinking for OPSLMW (Table 2). The minor increase in molecular weight from 1.1 kDa to 1.4 kDa is attributed to the effect of the installed groups on the backbone, as the overall dispersity of the material is not significantly affected. In contrast, GPC analysis reveals a substantial decrease in molecular weight for both OPSMMW (23.9 kDa to 14.8 kDa) and OPSHMW (364 kDa to ∼20 kDa), concomitant with an increase in dispersity (Table 2). The introduction of polar groups is known to potentially lead to secondary column interactions beyond size-based separation, such as electrostatic interactions with the stationary phase.^31,32^ To rule out this possibility, we conducted GPC analysis in various solvents and consistently observed a decrease in molecular weight, independently of the GPC eluent (CHCl_3_, THF, DMF, and DMF with 10% LiCl). This suggests that the decrease in molecular weight is not due to changes in the polymer’s hydrodynamic volume or solvation effects induced by the polar groups, but rather due to actual chain scission. Since no small molecules or gaseous by-products are detected during the reaction, we suspect a random chain scission mechanism rather than chain-end cleavage, which would also be consistent with the observed increase in dispersity. Surprisingly, despite this apparent bond cleavage, additional end-groups, including aldehydes or carboxylic acids typically expected in such cases, are not detected by multinuclear 1D and 2D NMR spectroscopy. We are currently investigating these discrepancies further.

**Table 2:** Gel permeation chromatography (GPC) results of the oxyfunctionalised PS materials

Entry	Sample	FG%	*M*_w_ (kDa)	*M*_n_ (kDa)	*Đ* (*M*_w_/*M*_n_)
1	PSLMW	0	1.1	1.0	1.1
2	OPSLMW	2.2–4.4	1.4	1.2	1.2
3	PSMMW	0	23.9	23.2	1.0
4	OPSMMW	0.7–1.3	16.8	12.2	1.4
5	PSHMW	0	>364.0	163.3	2.2
6	OPSHMW	2.9–5.7	14.7	5.9	2.5
7	OPSHMW	2.8–5.4	19.5	7.1	2.8
8	OPSHMW	2.3–4.6	17.0	6.3	2.7

Analysis of the glass-transition temperature (*T*_g_) of the OPS materials showed almost identical thermal properties compared to the parent polymers (Table 3). For the OPSHMW samples *T*_g_ is ∼10–15 °C higher than expected given how the observed backbone cleavage would be expected to impact this parameter (see below).^33,34^ OPSLMW, which didn’t suffer from backbone cleavage, showed an increase in *T*_g_ from 37.5 °C to 51.3 °C (Table 3), in line with previous research on low molecular weight PS.^16^ As typically observed in literature for polar group functionalised hydrocarbon polymers, the thermal decomposition (*T*_d(90 wt%)_) of the oxidised polymers decreases with an increasing FG% compared to the parent polymeric material (Table 3). The decrease in molecular weight for the high *M*_w_ PS samples is also expected to assist here in a decrease in the observed *T*_d_. As the Δ*T*_d max_ is only ∼70 °C, the oxyfunctionalised materials would still be thermally stable enough for use in various applications.

**Table 3:** Thermal property results of the oxyfunctionalised PS materials

Entry	Sample	FG%	*T*_g_ (°C)	*T*_d(90 wt%)_ (°C)
1	PSLMW	0	37.5	396.4
2	OPSLMW	2.2–4.4	51.3	322.6
3	PSHMW	0	104.6	404.1
4	OPSHMW	2.9–5.7	104.8	341.5
5	OPSHMW	2.8–5.4	104.6	348.8
6	OPSHMW	2.3–4.6	104.7	335.2

### Oxyfunctionalisation of polybutadiene

**Scheme 3:**
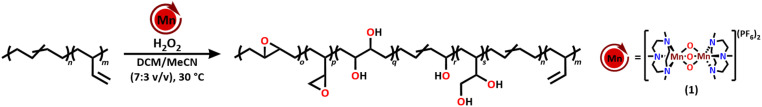
Catalytic oxyfunctionalisation conditions tested on PBD using the tri-oxo (1) bridged analogue of MnTACN.

Next to oxidation of aliphatic C–H bonds, Mn(TACN) is also known to convert unsaturated bonds to epoxides and diols.^26,27^ In MeCN the epoxides formed are typically less susceptible to ring opening and are therefore often the major product observed, while in more acidic media diol formation becomes preferred.^35,36^ As we used the same solvent system as for PS oxyfunctionalisation (PBD also did not dissolve in aqueous media), we expect minor epoxide hydrolysis. We first investigated the oxidation reaction on PBD8020 (Scheme 3), which contains three distinct types of unsaturated bonds in this polymer, potentially offering insight into the chemoselectivity of the catalyst.

**Fig. 3:**
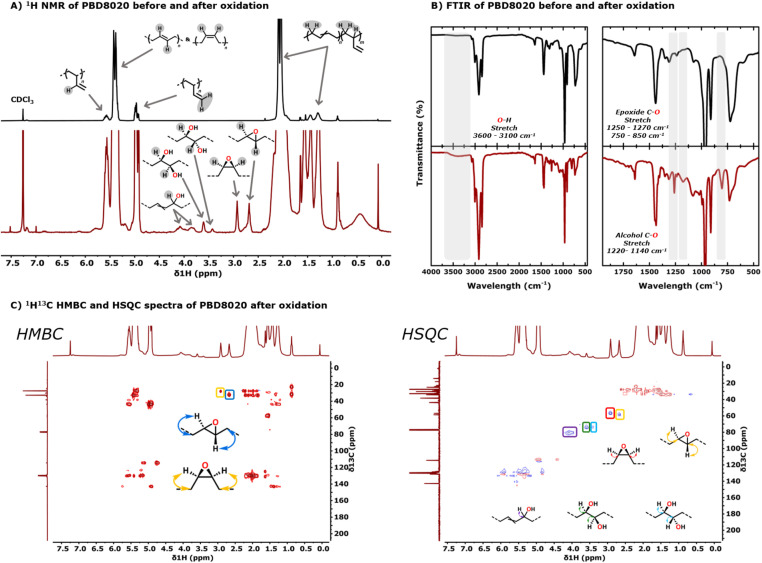
(A) ^1^H NMR spectra of unfunctionalised PBD8020 (black trace) and oxyfunctionalised PBD8020 (red trace). (B) FTIR spectra of unfunctionalised PBD8020 (black trace) and oxyfunctionalised PBD8020 (red trace). (C) ^1^H^13^C HMBC and HSQC spectra of oxyfunctionalised PBD8020.

Compared to PS, oxyfunctionalisation of PBD8020 was possible with a catalyst loading as low as 0.03 mol%. With this catalyst loading the ^1^H NMR spectrum of the oxidised polymer shows two new broad resonances at 2.91 ppm and 2.67 ppm, attributed to the *cis* and *trans* epoxides, respectively (Fig. 3A).^19,20^ This is further supported by the ^1^H^13^C HMBC and ^1^H^13^C HSQC spectra where we observe the ^2^*J*-coupling of the epoxide protons with the neighbouring aliphatic carbons at 28.2 ppm for the *cis* epoxide and 32.1 ppm for the *trans* epoxide; and the ^1^*J*-coupling of the epoxide protons with tertiary carbons at 57.0 ppm for the *cis* epoxide and 59.0 ppm for the *trans* epoxide (Fig. 3C).^19,22,37^ Additionally, the formation of the epoxides is supported by the C–O vibration for an epoxide at ∼1260 cm^−1^ and 780 cm^−1^ in FTIR (Fig. 3B). Epoxidation of the terminal unsaturated bonds is surprisingly not observed as we do not detect a carbon resonance at ∼47 ppm or ^1^*J*-coupling in the epoxide region in ^1^H NMR with such a lower carbon shift.^38^ Additionally, we observe the appearance of two sets of resonances at 3.60 ppm and 3.41 ppm in ^1^H NMR corresponding to the *cis* and *trans* diol, most likely formed by *cis*-dihydroxylation and ring opening of the epoxides, respectively.^39–41^ This is further supported by the observed ^1^*J*-coupling of the diol protons with tertiary carbons at 74.6 ppm in ^1^H^13^C-HSQC. Lastly, we observe two new resonances at 4.07 ppm and 3.84 ppm, assigned to a secondary alcohol alpha to the unsaturated bond as a result of allylic oxidation or possible migration of the unsaturated bond.^42,43^ This assignment is further supported by the observed ^1^*J*-coupling of the protons with a tertiary carbon at 80.5 ppm in ^1^H^13^C HSQC and by the O–H stretch at ∼3450 cm^−1^ and the C=C stretches at ∼1650 cm^−1^. There is no indication for over-oxidation of the alcohol groups to carbonyls, as a typical resonance at ∼200 ppm in ^13^C NMR nor a ^2^*J* or ^3^*J* coupling is not observed in ^1^H^13^C HMBC. This is further supported by the FTIR spectrum, in which C=O vibrations are not observed at ∼1720 cm^−1^. Additionally, no resonances for oxyfunctionalisation are observed in the absence of the catalyst or hydrogen peroxide.

Integrating the resonances for the epoxides, diols and allylic alcohol, shows that the epoxides (FG_*cis*-epoxide_ = 0.4% FG_*trans*-epoxide_ = 0.9%) and the allylic alcohol (FG_alpa-OH_ = 1.2%) are predominantly formed. Next to that, we observe minor *cis*-dihydroxylation (FG_*cis*-diol_ = 0.1%) and trace amounts of ring opening of the *trans* epoxide to the *trans*-diol (FG_*trans*-diol_ = 0.03%), making the FG_total_ = 2.6% (Table 4, entry 3). Similarly to the functionalisation of PS we observe a significant increase of the FG_total_% from 2.6% to 15.2% when the H_2_O_2_ is added portion wise to the reaction mixture instead of in 1 batch using the same catalyst loading (Table 4, entry 4). This increase in oxyfunctionalisation is predominantly caused by an increase in allylic alcohol formation. Such allylic oxidation typically occurs at high H_2_O_2_ concentrations, which is in line with our observations when H_2_O_2_ is added in two portions with a 96 h interval.^44^ Under the latter conditions, FG_alpha-OH_ increases further to 10.4% and the FG_total_% increases up to 17.9% (Table 4, entry 5).

**Table 4:** Investigation of the catalytic oxidation conditions on the oxyfunctionalisation of PBD materials

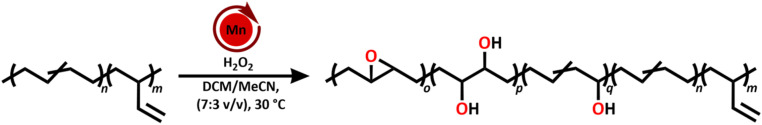											
Entry	Sample	Cat. (mol%)	Portions H_2_O_2_^a^	Reaction time (h)	FG_*cis*-epoxide_%	FG_*trans*-epoxide_%	FG_*cis*-diol_%	FG_*trans*-diol_%	FG_allylic-OH_%	FG_total_%	Yield recovered material (%)
1	PBD8020	0	1	72	0	0	0	0	0	0	93
2	PBD8020	0.03	0	72	0	0	0	0	0	0	94
3	PBD8020	0.03	1	72	0.4	0.9	0.1	0.03	1.2	2.6	87
4^b^	PBD8020	0.03	3	144	1.3	3.5	0.6	0.3	9.6	15.2	84
5^c^	PBD8020	0.03	2	192	2.3	4.2	0.7	0.3	10.4	17.9	86
6^b^	PBD1090	0.03	3	144	0	0	0	0	0	0	89

a In total 2.0 equivalents of H_2_O_2_ were used in respect to the monomeric unit for each experiment.

b 3 × 0.67 equivalents of H_2_O_2_ were added with a 24-hour time interval.

c 2 × 1.0 equivalents of H_2_O_2_ were added with a 96 h time interval.

The absence of functionalisation of the terminal double bonds in PBD8020 is somewhat surprising given that the catalyst is known to oxidise terminal alkenes such as those in 2,4-dimethyl-1-heptene.^44^ This prompted us to investigate this further using a different grade of PBD, namely PBD1090, which contains 10% *cis* and *trans* 1,4-type linkages and 90% vinyl 1,2-type linkages. When subjected to the same reaction conditions as PBD8020, no oxyfunctionalisation of PBD1090 was observed by ^1^H NMR. This may be due to the lower electron density of these double bonds or their limited accessibility, potentially caused by chain folding that hinders catalyst access (Table 4, entry 6).^19,25^

Rewardingly, GPC analysis of the oxyfunctionalised PBD8020 shows no evidence for any (major) undesired backbone cleavage (nor crosslinking) of the materials (Table 5). Additionally, there is no indication of the formation of smaller alkane or alkene fragments by NMR, which emphasises the well-behaved oxidation of these unsaturated polymers.

**Table 5:** Gel permeation chromatography (GPC) and thermogravimetric analysis (TGA) of the oxyfunctionalised PBD materials

Entry	Sample	FG%	*M*_w_ (kDa)	*M*_n_ (kDa)	*Đ* (*M*_w_/*M*_n_)	*T*_d(90 wt%)_ (°C)
1	PBD8020	0	10.7/22.8	10.1/22.0	1.1/1.0	406.6
2	OPBD8020	2.6	10.0/29.1	7.9/25.5	1.3/1.1	392.2
3	OPBD8020	15.2	10.2/31.9	8.3/27.5	1.2/1.2	366.2
4	OPBD8020	17.9	9.7/29.4	7.7/25.6	1.2/1.1	350.1

The thermal properties, similar to PS, showed the *T*_d(90 wt%)_ decreases upon (increasing) oxyfunctionalisation of the polymer backbone. The largest *T*_d(90 wt%)_ decrease of 56.5 °C is observed for the material bearing a FG_Total_ = 17.9%. Comparison of our results with previously reported related polyenes containing similar allyl alcohol groups or PBDs with high epoxy FG% (≥26% epoxy groups), suggests that the presence of the allyl alcohol groups is the primary factor responsible for decreasing thermal stability; epoxy groups are not thought to significantly affect the polymers’ stability.^43,45^ Unfortunately, the *T*_g_’s of the OPBD8020 polymers could not be measured as they are outside of the range accessible with our DSC. Based on previous work on related oxyfunctionalised polyenes and PBDs, we expect the *T*_g_ of the OPBD8020 materials to increase with increasing oxyfunctionalisation, as polar groups such as alcohols, ketones and epoxides enhance intermolecular interactions and increase the backbone rigidity.^43,45^

### Oxyfunctionalisation of the SBS block-co-polymer

**Fig. 4:**
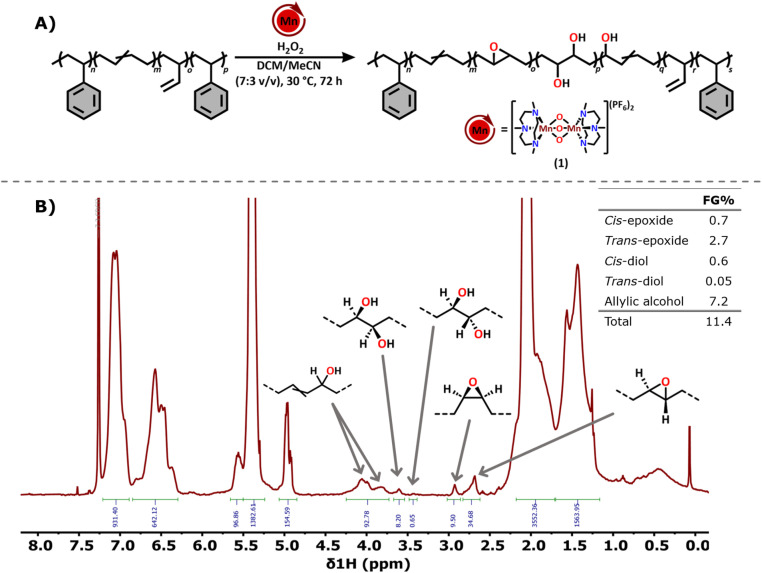
(A) Catalytic oxyfunctionalisation conditions tested on SBS using the tri-oxo (1) bridged analogue of MnTACN. (B) ^1^H NMR spectrum after the oxyfunctionalisation of styrene–butadiene–styrene (SBS) block-co-polymer, with the corresponding FG% of each installed oxyfunctional group.

Encouraged by the oxyfunctionalisation of PS and PBD8020, we lastly investigated the functionalisation of a styrene–butadiene–styrene (SBS) block-co-polymer, with 30 wt% styrene, to explore the chemoselectivity of the catalyst in the presence of both aliphatic and unsaturated C–H bonds. A catalyst loading of 1 mol% was used, so that oxyfunctionalisation of both the styrene and the butadiene blocks could be expected (Fig. 4A). After subjecting the SBS polymer to the oxidation conditions, with hydrogen peroxide having been added dropwise in one batch, the ^1^H NMR and ^1^H^13^C 2D NMR data show that the butadiene block was selectively oxidised while the styrene block remained untouched (Fig. 4B). Similar to PBD8020, a high selectivity towards the *trans* epoxide (2.7%) and the allylic alcohol (7.2%) was observed, with minor amounts of *cis* epoxide (0.7%) and *cis*-diol (0.6%) also being detected as well as a trace amount of the *trans*-diol as a result of ring opening of the *trans* epoxide (0.05%). Taken together, this amounts to an overall FG_total_ of 11.4%.

Similar to the results observed for PS, GPC analysis of the modified SBS revealed a significant decrease in molecular weight after oxyfunctionalisation (Table 6), which could be attributed to backbone cleavage in the PS block. However, consistent with previous observations, no gaseous products, small molecule formation, nor appearance of additional end groups could be detected by NMR spectroscopy, findings that contradict the GPC results. We are currently investigating this in more detail. The thermal decomposition temperature of the oxyfunctionalised SBS decreased similar to the PS and PBD homopolymers by 29 °C as a result of the installed functional groups as well as the backbone cleavage. Also, for the (modified) SBS, any changes in *T*_g_ could unfortunately not be measured, as this parameter is again also not accessible with our equipment. As noted above, an increase in *T*_g_ compared to the unfunctionalised polymer would be expected.

**Table 6:** Gel permeation chromatography (GPC) and thermogravimetric analysis (TGA) of the oxyfunctionalised SBS materials

Entry	Sample	FG%	*M*_w_ (kDa)	*M*_n_ (kDa)	*Đ* (*M*_w_/*M*_n_)	*T*_d(90 wt%)_ (°C)
1	PS-PBD-PS	0	190.6/89.5/15.5	183.6/87.9/15.2	1.0/1.0/1.0	407.2
2	PS-OPBD-PS	11.4	22.7	18.7	1.2	377.9

### Backbone cleavage of oxyfunctionalised polybutadiene

**Fig. 5:**
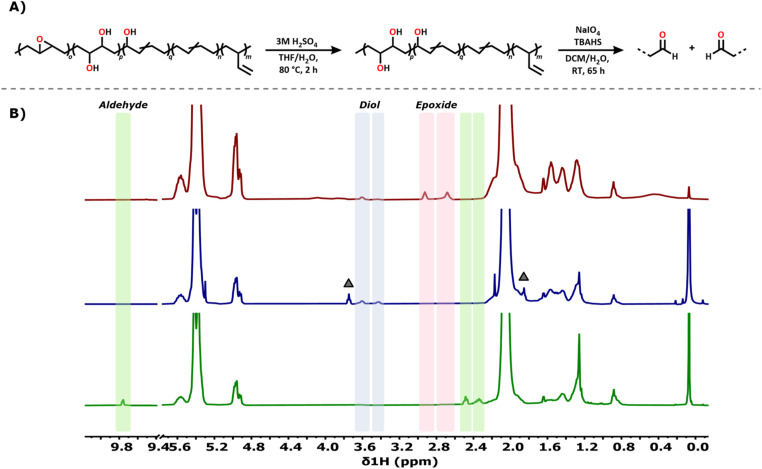
(A) Ring-opening of the epoxides installed on the PBD backbone using sulfuric acid and subsequent oxidative cleavage of the diol-moieties. (B) ^1^H NMR spectra of oxyfunctionalised PBD8020 (red trace), fully alcohol functionalised PBD8020 after ring opening of the epoxides (blue trace) and oxidatively cleaved PBD8020 to di-aldehydes (green trace). The Δ in the blue trace is residual THF.

Finally, we looked into a possible upcycling or degradation strategy by oxidative cleavage of the diol motifs installed on PBD.^20^ Such a cleavage procedure would also provide insight into the spacing of the oxyfunctional groups through analysis of the resulting products mixture. To this end, we first opened the epoxides using sulfuric acid (H_2_SO_4_) to generate the corresponding diols (Fig. 5A). These diols were then cleaved to aldehydes using sodium periodate (NaIO_4_) in the presence of tetrabutylammonium hydrogen sulfate (TBAHS) as a phase transfer catalyst. Monitoring the reaction, the diol resonance at ∼3.5 ppm in ^1^H NMR spectrum disappeared, while new signals emerged: a broad resonance at 9.75 ppm, a triplet at 2.48 ppm and a multiplet at 2.33 ppm, features characteristic of aldehydes (Fig. 5B). This is further supported by ^13^C NMR and ^1^H^13^C HMBC spectra, which show a new resonance for a quaternary carbon at 202.5 ppm coupling to ^1^H signals at 2.48 ppm and 2.33 ppm. As no gas formation, pressure build up, or resonances indicative of carboxylic acid were observed, we concluded that overoxidation does not occur under these conditions.

Having confirmed that the backbone is cleaved, we analysed the backbone length of the obtained di-aldehyde fractions. NMR analysis did not allow determination of the backbone lengths of the di-aldehyde products due to signal overlap. GPC analysis did offer insight into the distribution of di-aldehydes, as multiple peaks were detected of decreasing backbone length compared to its parent PBD polymer. The minor high *M*_w_ fractions range from 10.0 kDa to 4.0 kDa and major backbone lengths rage from ∼1.7 kDa to 200 Da. We expect that smaller di-aldehyde fractions, *i.e.* lower than 200 Da, are formed as well, but cannot be detected on the particular GPC column used. As the sample still contained high molecular weight backbone fragments, GC analysis of low molecular weight di-aldehyde fractions was unfortunately not possible. Nevertheless, the observed decrease in molecular weight by GPC suggests that the installed functional groups are randomly distributed on the PBD backbone with a spacing ranging from 4 up to 32 monomeric units and that the oxyfunctional groups might not be evenly distributed on all polymer chains. The oxidative decomposition strategy does open possibilities for chemical degradation of PBD backbone materials and might allow for selective recycling of butadiene-bearing co-polymers.

## Conclusion

Mn(TACN) has been shown to effectively oxyfunctionalise a range of styrene- and butadiene-based polymers using hydrogen peroxide as a green oxidant. With this robust and versatile catalyst, we were able to install various oxygen-containing functional groups on the polymer backbones under mild reaction conditions. High degrees of oxyfunctionalisation were achieved, with FG_total_ values reaching up to 17.9% for PBD, 5.7% for PS, and 11.4% for SBS. Hydroxyl and ketone groups were introduced to PS, while epoxides, alcohols and diols could be installed on PBD and selective oxyfunctionalisation of butadiene in SBS is achieved. For the latter, selectivity was notably high towards the *trans*-epoxide and allylic alcohol. Oxyfunctionalisation did not affect the thermal properties of the polymeric materials much, with the biggest impact being on the thermal decomposition which was lowered by 30 °C to 70 °C depending on the extent of functional group decoration. GPC analysis confirms that the oxidation of PBD and low molecular weight PS does not result in significant backbone cleavage or crosslinking, whereas the data suggests backbone cleavage in the high molecular weight PS and SBS samples.

The modified polymers offer various opportunities for further PPM and depolymerisation, as demonstrated by the clean conversion of the epoxides on PBD to diols by simple ring opening with a strong acid and the subsequent selective oxidative cleavage of the backbone through the installed diol motifs. Analysis of the backbone cleavage products suggests that the oxyfunctional groups are randomly spaced on the polymer backbone. More generally, the effective scission of the oxidised PBD opens possibilities for recycling of butadiene-based polymers.

## Author contributions

Project design was done by M. Otten, A. Thevenon and P. C. A. Bruijnincx. Synthesis, characterisation, thermal property analysis and gel permeation chromatography (GPC) experiments were performed by M. Otten, J. Hendriks and N. Kalános. All authors discussed the results and assisted during manuscript preparation.

## Conflicts of interest

There are no conflicts to declare.

## Supplementary Material

FD-262-D5FD00093A-s001

## Data Availability

The data related to the work described in this paper is available in the Yoda repository using the following link: https://doi.org/10.24416/UU01-982M0E.
