# Supplementary material for: Post-polymerisation oxyfunctionalisation of styrene and butadiene-based (co-)polymers using a homogeneous manganese catalyst
Source: Faraday Discuss. 2025 Jul 11;262:349–66. doi: 10.1039/d5fd00093a (PMC12442954; doi:10.1039/d5fd00093a)
Supplement: FD-262-D5FD00093A-s001 [file FD-262-D5FD00093A-s001.pdf]

Supplementary Information (SI) for Faraday Discussions.
This journal is © The Royal Society of Chemistry 2025

Supporting Information

Post-Polymerisation Oxyfunctionalisation of Styrene and Butadiene-Based (Co-)Polymers using a Homogeneous Manganese Catalyst

Maartje Otten[a], Jeroen Hendriks[a], Nino Kalános[a], Arnaud Thevenon[a*] and Pieter C.A. Bruijnincx[a*]

a) Organic Chemistry & Catalysis, Institute for Sustainable and Circular Chemistry, Faculty of Science, Utrecht University, Universiteitsweg 99, 3584 CG, Utrecht, The Netherlands

* Corresponding Authors: Arnaud Thevenon and Pieter C.A. Bruijnincx, Organic Chemistry & Catalysis, Institute for Sustainable and Circular Chemistry, Faculty of Science, Utrecht University, Universiteitsweg 99, 3584 CG Utrecht, The Netherlands; Email: p.c.a.bruijnincx@uu.nl; a.a.thevenon-kozub@uu.nl

Table of Contents

1. General 2
2. Oxyfunctionalisation reactions 3
  2.1 Synthesis of mono-oxo-di-acetate Mn(TACN) (2) 3
  2.2 Catalytic oxidation reaction of PS with mono-oxo-di-acetate Mn(TACN) (2) 5
  2.3 Catalytic oxidation reaction of PS with tri-oxo Mn(TACN) (1) 7
  2.4 Catalytic oxidation reaction of PBD8020 with tri-oxo Mn(TACN) (1) 18
  2.5 Catalytic oxidation reaction of SBS with tri-oxo Mn(TACN) (1) 26
  2.6 Epoxide opening to diol on oxypolybutadiene 29
  2.7 Oxidative cleavage of the diol motives on oxypolybutadiene 31
3. DSC and TGA Data 37
4. GPC Data 49
References 62

1. General

Polybutadiene (100% 1,2-type unsaturated bonds, M_w = 5.0 kDa and 80% 1,4-type, and 20% 1,2 unsaturated bonds, M_w = 10.7/22.8 kDa), polystyrene (M_w = 24 kDa) and styrene-butadiene-styrene block-co-polymer (M_w = 191/90/16 kDa, trimodal weight distribution) were acquired from sigma Aldrich. Polystyrene (M_w = < 364 kDa, and M_w = 1.1 kDa) was acquired from Fischer scientific. The used Mn(TACN) catalyst was kindly supplied by prof.dr. Ronald Hage and CATEXCEL BV. All chemicals were acquired from commercial sources and used as received. *trans*-4-octene, hydrogen peroxide (35% in H_2O) and tetrabutylammonium hydrogensulphate were purchased from Sigma-Aldrich. Dichloromethane, tetrahydrofuran and acetonitrile were acquired from Biosolve. Sodium periodate and sulphuric acid were purchased from VWR Chemicals. All reagents and starting materials were purchased from commercial sources and used without further purification, except when specified. Deuterated solvents were purchased from the Cambridge Isotope Laboratory Incorporation (Cambridge, USA) or Sigma-Aldrich and used as received.

NMR spectroscopy (^{1}H, $^{13}C\{^{1}H\}$, ^{1}H-^{13}C and ^{1}H-^{15}N) was conducted using a 400 MHz Varian spectrometer equipped with AutoX probe and Agilent ProTune probe tuning accessory or a 400 MHz Jeol EZCL G spectrometer with a HFX probe. ^{1}H and ^{13}C NMR chemical shift are reported in the standard δ notation of part per million (ppm) and are referenced to a residual peak of the solvent, as determined relative to SiMe4. Infrared spectroscopy was conducted using a PerkinElmer SpectrumTwo FT-IR Spectrometer equipped with an ATR-probe. Peaks are annotated by (w), (m) and (s) to indicate weak, medium and strong signals, respectively.

Thermogravimetric analysis (TGA) measurements were performed on a TA Instruments TGA Q50 Thermographic Analyser under nitrogen atmosphere on a platinum pan and analysed using TA Instruments Universal Analysis 2000 software. The thermal degradation was investigated with approximately 10-20 mg sample, loaded onto the pan and heated from ambient temperature to 700 °C with a rate of 20 °C/min. Modulated Differential Scanning Calorimetry (MDSC) measurements were performed on a TA Instruments Discovery DSC equipped with a TA Instruments Refrigerated Cooling System 90 and analysed with TA Instruments Trios Software (version 5.7.1.74). Tzero low mass aluminium pans were loaded with approximately 3-5 mg of sample and closed with a Tzero aluminium lid. Samples were first kept at 25 °C and thereafter cooled down at a rate of 2.00 °C/min to -90 °C. After 10 min at -90 °C, the sample was heated to 80 °C at a rate of 2.00 °C/min. After 10 min at 80 °C, the sample was cooled down to 25 °C. The modulation was set at 1 °C/min.

GPC measurements were recorded on as Waters Alliance e2695 Separations Module equipped with 3 x PLGel mixed-D columns in series, a guard column and a Waters 2489 UV/visible Detector or a Shimadzu (SCI-40 controller module, DGU-203 degassing unit, SIL-40 autosampler, CTO-40C column oven, RID-20A refractive index detector) equipped with an Agilent Technologies PLgel 5µm Guard 50 x 7.5 mm guard column and two Agilent Technologies PLgel 5µm MIXED-D 300 x 7.7 mm columns. All polymer samples were dissolved in the corresponding solvent (THF, chloroform, or DMF) and filtered with a 45 µm PTFE syringe filter prior to the GPC analysis. Refractive index or UV detection at 254 nm was performed and molecular weight determinations were based on calibrations with polystyrene standards (**Section 4**).

2. Oxyfunctionalisation reactions

2.1 Synthesis of mono-oxo-di-acetate Mn(TACN) (2)

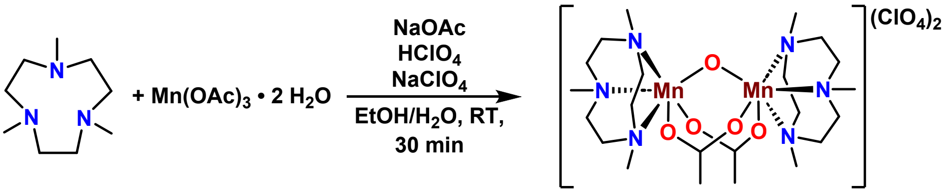

This synthesis was based on literature.[1] A 100 mL Schlenk was charged with 1,4,7-trimethyl-1,4,7-triazacyclononane (203.5 mg, 1.12 mmol, 1.7 eq.) whereafter it was dissolved in an ethanol/water (5 mL/0.9 mL) mixture. $Mn(OAc)_2 \cdot 2H_2O$ (180.6 mg, 0.67 mmol, 1 eq.) and NaOAc (400 mg, 4.88 mmol, 7.3 eq.) were added resulting in a dark brown/red solution, which was stirred at ambient temperature for 30 minutes before the addition of approximately 15 drops of $HClO_4$ using a glass pipette. Then $NaClO_4$ (600 mg, 4.90 mmol, 7.3 eq.) was added, followed by another 8 drops of $HClO_4$. The reaction mixture turned more red throughout the addition of the acid. To isolate the compound, the solvent was evaporated in vacuo and a dark red solid was obtained.

^{1}H NMR (paramagnetic settings) (400 MHz, MeCN-d_3) δ 80.61 (s), 70.32 (s), 67.39 (s), 36.89 (s), 35.28 (s), 21.22 (s), -78.18 (s), -90.70 (s), -95.68 (s) ppm.

ATR-IR 621 (m), 1076 (s, br), 1417 (w, br), 1574 (m, br), 1719 (w, br), 2872 (w), 3612 (w) cm^{-1}.

UV-Vis (MeCN): λ_{max} = 487, 524, 723 nm.

ESI-MS (MeCN): (low fragmentation setting) m/z = 172.1 {[tmtacn+H]$^+$, calc. 172.3}, 293.0 {[$Mn^{III}_2(\mu$-O)(μ-$CH_3CO_2)_2$(tmtacn)$_2$]$^{2+}$, calc 293.3}, 285.0 {[$Mn^{II}(CH_3CO_2)$-(tmtacn)]$^+$, calc. 285.19}

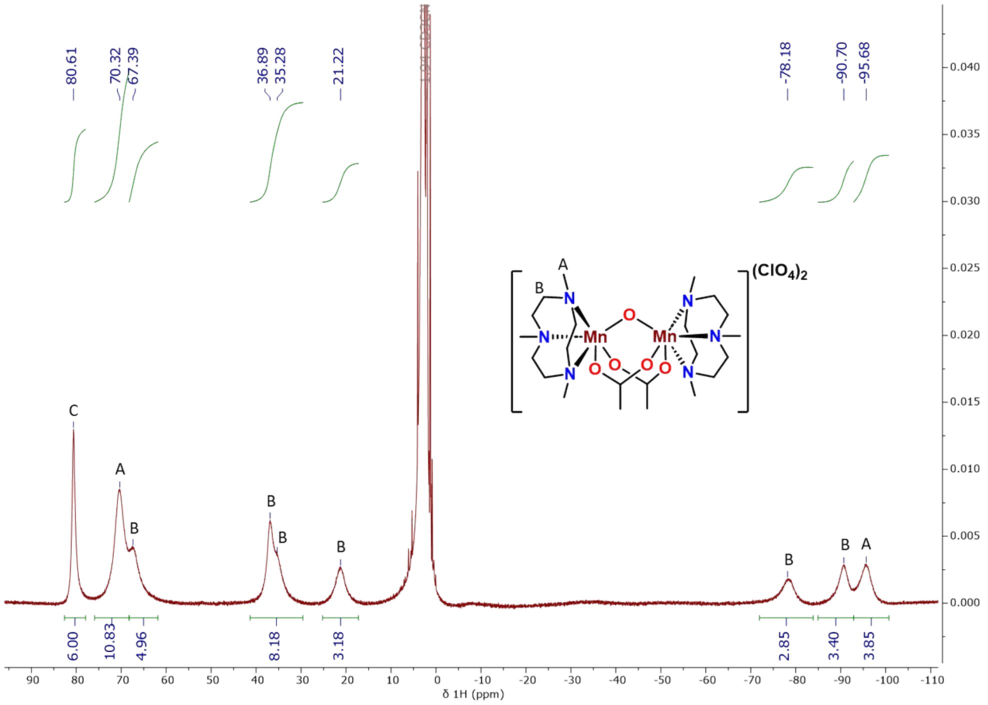

Figure S1: *^{1}H NMR spectrum with paramagnetic settings of [$Mn_2(\mu$-OAc)$_2(\mu$-O)(tmtacn)$_2$]($ClO_4)_2$ measured in MeCN-d_3 at 25 °C. Peaks were assigned as reported by Hage et al.*[2]

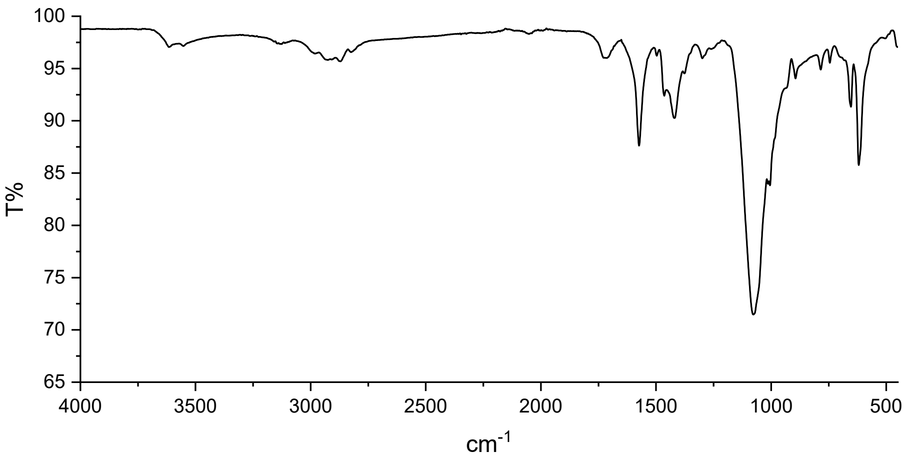

Figure S2: *FTIR spectrum of [Mn_2(μ-OAc)$_2$(μ-O)(tmtacn)$_2$](ClO_4)$_2$.*

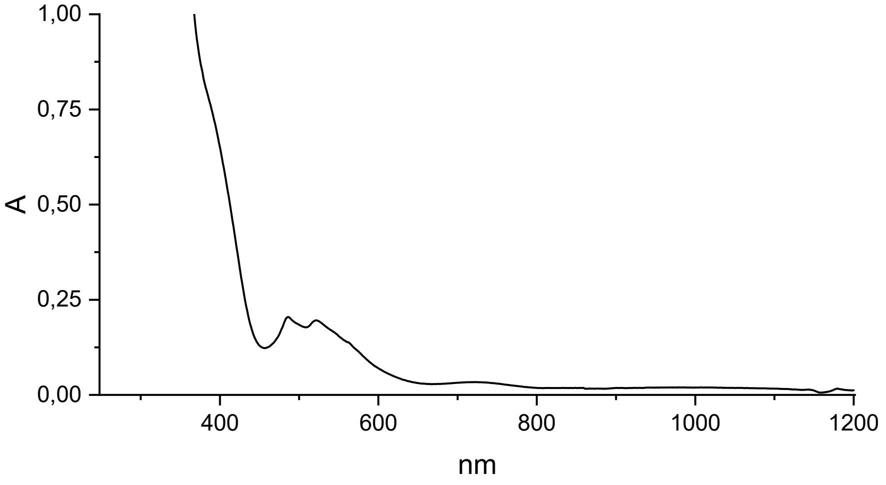

Figure S3: *UV-Vis spectrum of [Mn_2(μ-OAc)$_2$(μ-O)(tmtacn)$_2$](ClO_4)$_2$ measured in MeCN.*

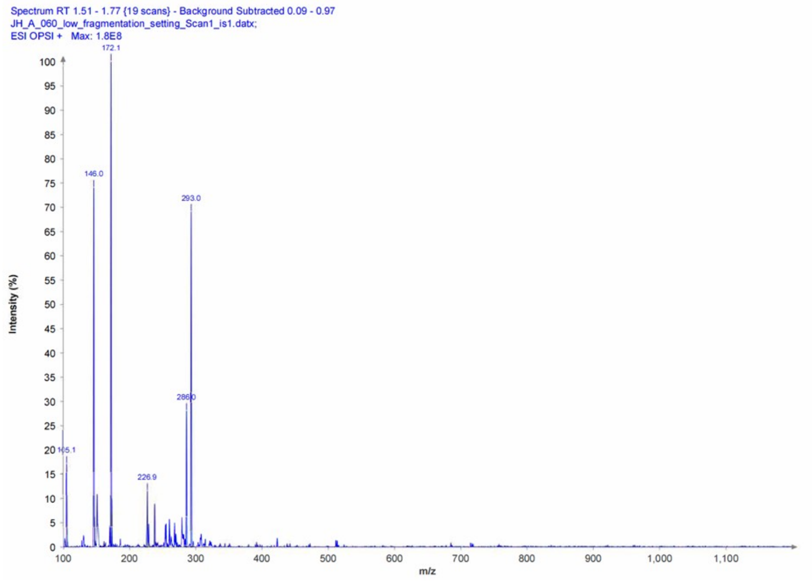

Figure S4: *ESI-MS spectrum of [Mn_2(μ-OAc)$_2$(μ-O)(tmtacn)$_2$](ClO_4)$_2$ measured in MeCN using low fragmentation settings.*

2.2 Catalytic oxidation reaction of PS with mono-oxo-di-acetate Mn(TACN) (2)

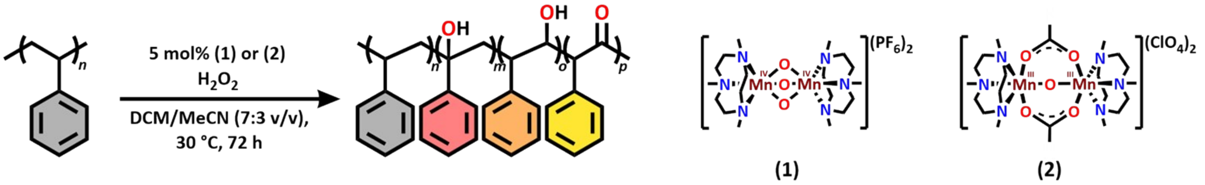

Oxidation reactions were performed based on literature.[3] A 20 mL vial was charged with polystyrene high M_w (48.0 mg, 0.461 mmol, 1 eq.) and dissolved in a mixture of DCM/MeCN (2.8 mL/1.2 mL) at 30 °C. The $[Mn_2(\mu\text{-}OAc)_2(\mu\text{-}O)(tmtacn)_2](ClO_4)_2$ (18.8 mg, 0.024 mmol, 5 mol%) was added and the opening of the vial was covered using parafilm. 1 mL of 35% H_2O_2 was added to the red-coloured solution containing the catalyst and substrate using a syringe pump in 1 hour. During the addition, the colour changed to yellow, and the mixture was left to stir for 72 hours at 30 °C. Afterwards, the reaction mixture was washed with brine and DCM, the organics were dried over Na_2SO_4 and the solvent was evaporated using a rotary evaporator. The obtained off-white solids were thereafter dried in a vacuum oven for 24 hours (Yield = 86%).

^{1}H NMR (400 MHz, 1,1,2,2-tetrachloroethane-d$_2$) δ 7.13 (s, 1H), 7.07 (s, 1H), 6.98 (s, 1H), 6.63 (s, 0.5H), 6.57 (s, 0.5H), 6.51 (s, 0.5H), 6.42 (s, 0.5H), 3.71 (s), 3.48 (s), 3.12 (s), 2.94 (s), 2.25 (s), 2.18 (s), 2.11 (s), 1.92 (s, 1H), 1.48 (s, 2H) ppm.

ATR-IR 697 (s), 755 (m), 1028 (w), 1452 (m), 1493 (m), 1600 (w), 1744 (w), 1771 (w), 1870 (w), 1947 (w), 2847 (w, br), 2920 (m, br), 3026 (m), 3061 (w), 3083 (w), 3515 (w, br) cm^{-1}.

DSC T_g = 106.85 °C

TGA T_d (90 wt%) = 356.3 °C, $T_{max\ wt\ loss}$ = 409.8 °C.

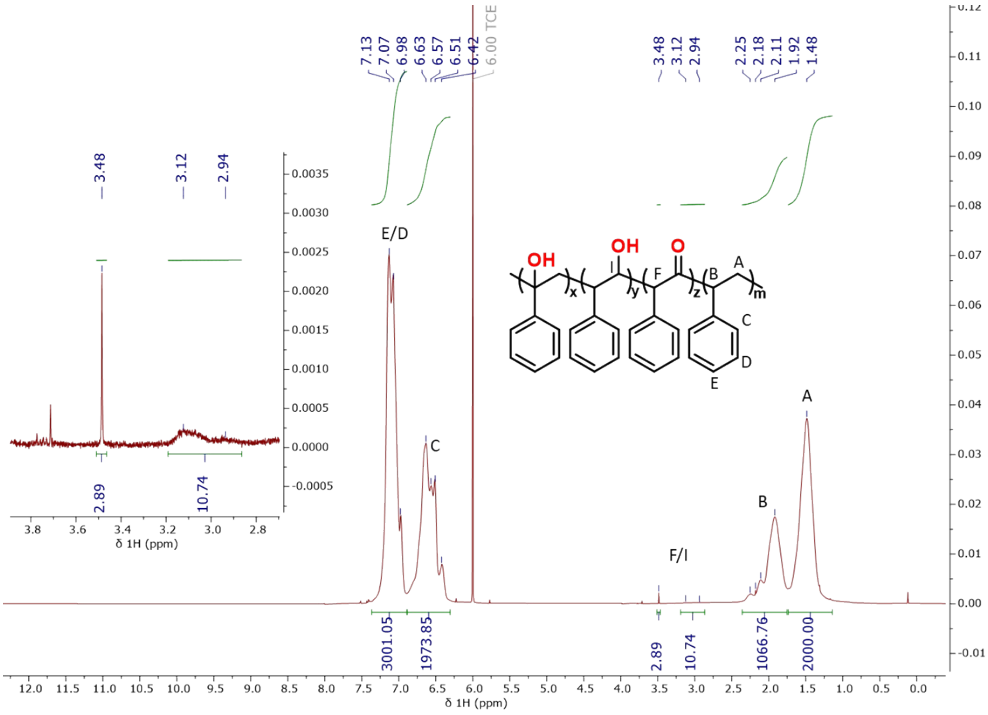

Figure S5: *^{1}H NMR spectrum of the oxidation of polystyrene using $Mn^{III}_2(tmtacn)_2$ measured in 1,1,2,2-tetrachloroethane-d2 at 25 °C.*

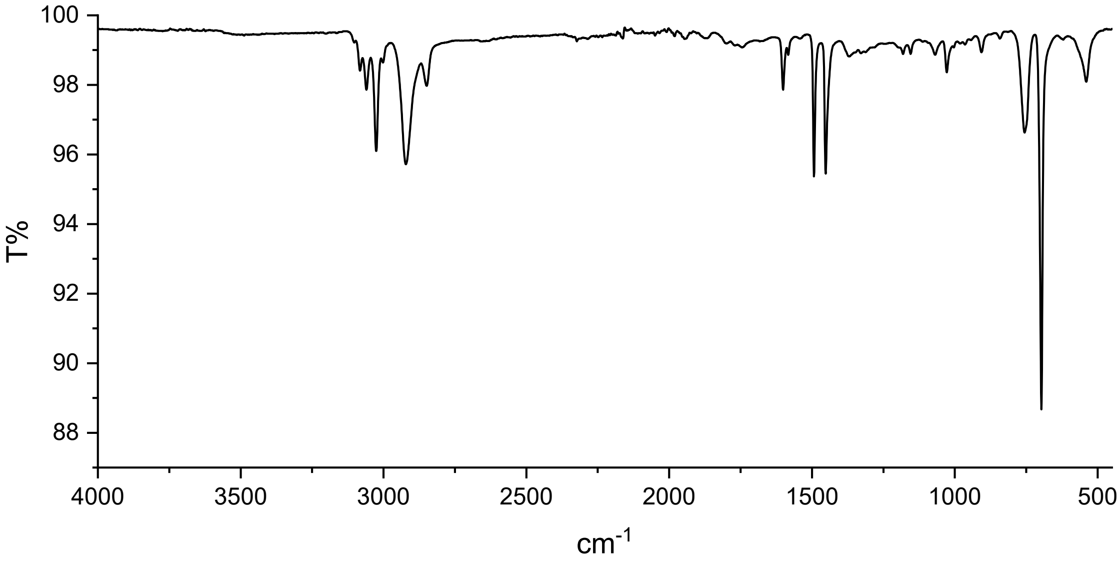

Figure S6: *IR spectrum of oxidised polystyrene using Mn$^{III}{}_2$(tmtacn)$_2$.*

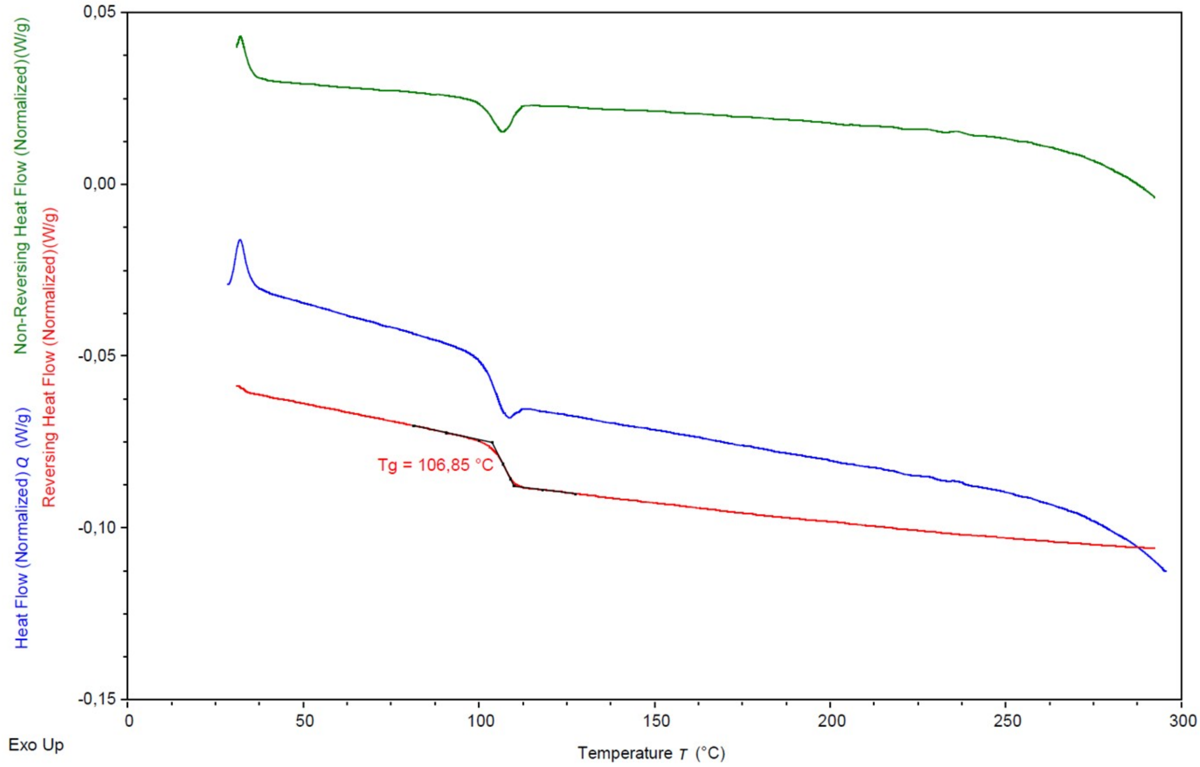

Figure S7: *DSC plot of oxidised polystyrene using Mn$^{III}{}_2$(tmtacn)$_2$. Red trace is reversing heat flow. Blue trace is Heat flow. Green trace is non-reversing heat flow.*

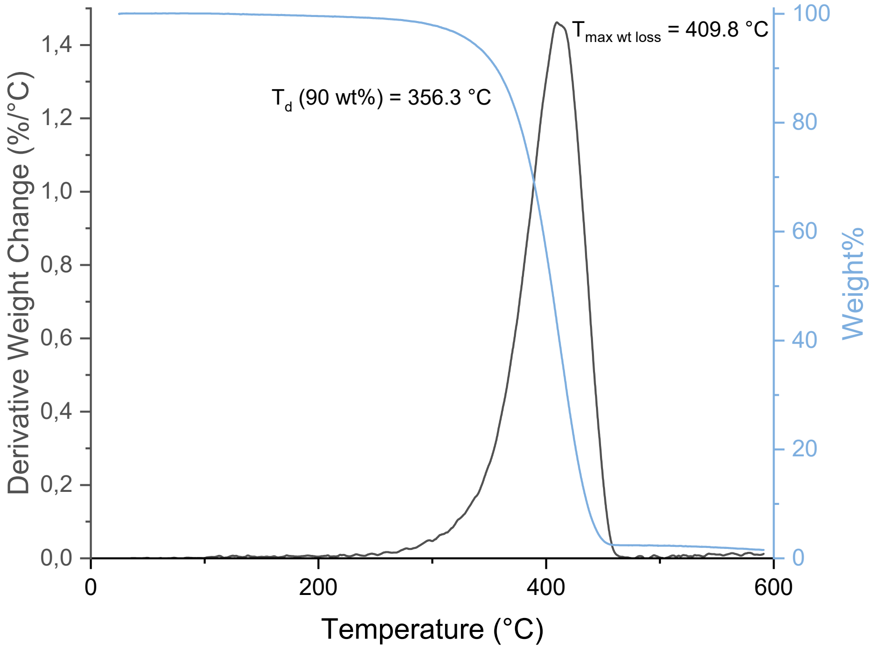

Figure S8: *TGA plot of oxidised polystyrene using* $Mn^{III}_2(tmtacn)_2$.

2.3 Catalytic oxidation reaction of PS with tri-oxo Mn(TACN) (1)

2.3.1 H_2O_2 addition in 1 batch (Table 1, Entry 5)

Oxidation reactions were performed based on literature.[3] A 50 mL flask was charged with polystyrene high M_w (480 mg, 4.61 mmol, 1 eq.) and dissolved in a mixture of DCM/MeCN (8 mL/2 mL) at 30 °C. The MnTACN (**1**) (187.7 mg, 0.23 mmol, 5 mol%) was added and the opening of the flask was covered using parafilm. 10 mL of 10% H_2O_2 was added to the red-coloured solution containing the catalyst and substrate using a syringe pump in 1 hour. During the addition, the colour changed to yellow, and the mixture was left to stir for 72 hours at 30 °C. Afterwards, the reaction mixture was washed with brine and DCM, the organics were dried over Na_2SO_4 and the solvent was evaporated using a rotary evaporator. The obtained off-white solids were thereafter dried in a vacuum oven for 24 hours. (see table below for functionalisation degree and yields)

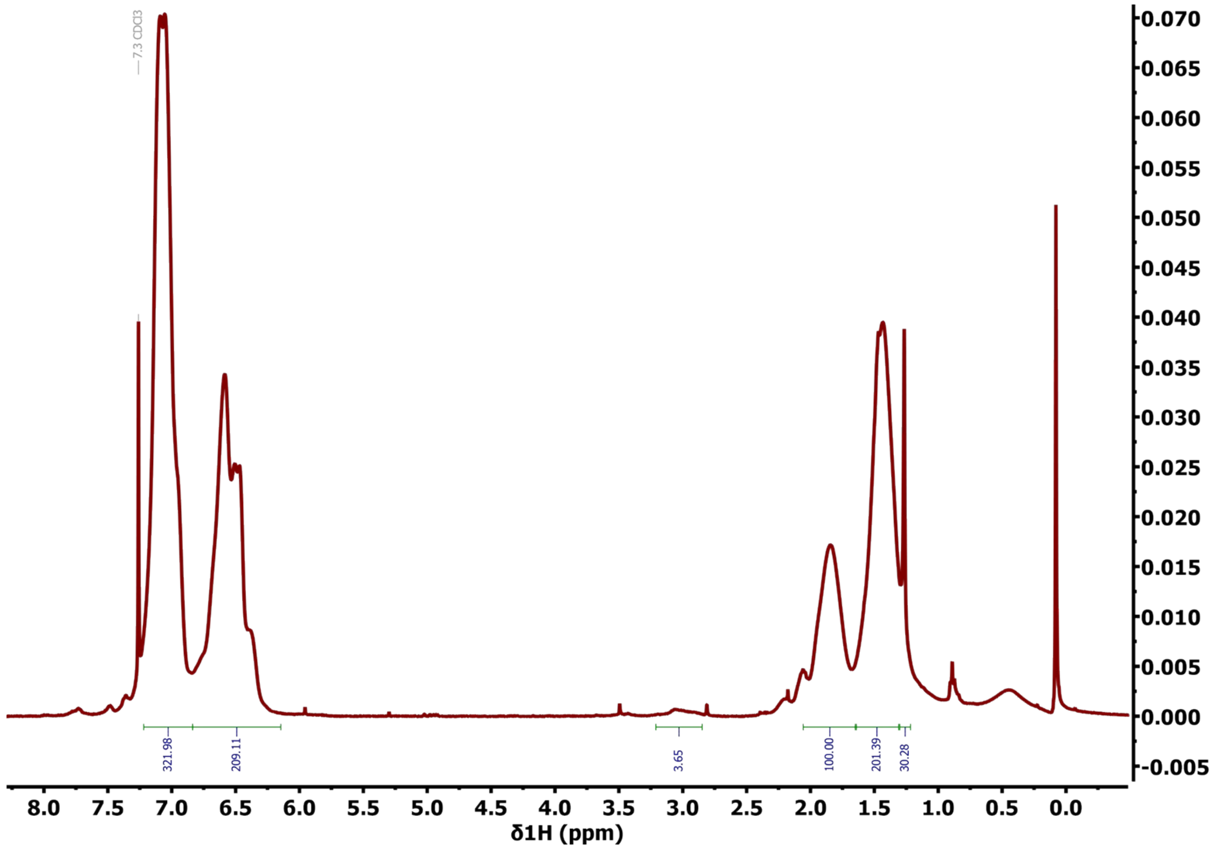

Figure S9: *¹H NMR spectrum of the oxidation of polystyrene using Mn(TACN) measured in chloroform-d at 25 °C.*

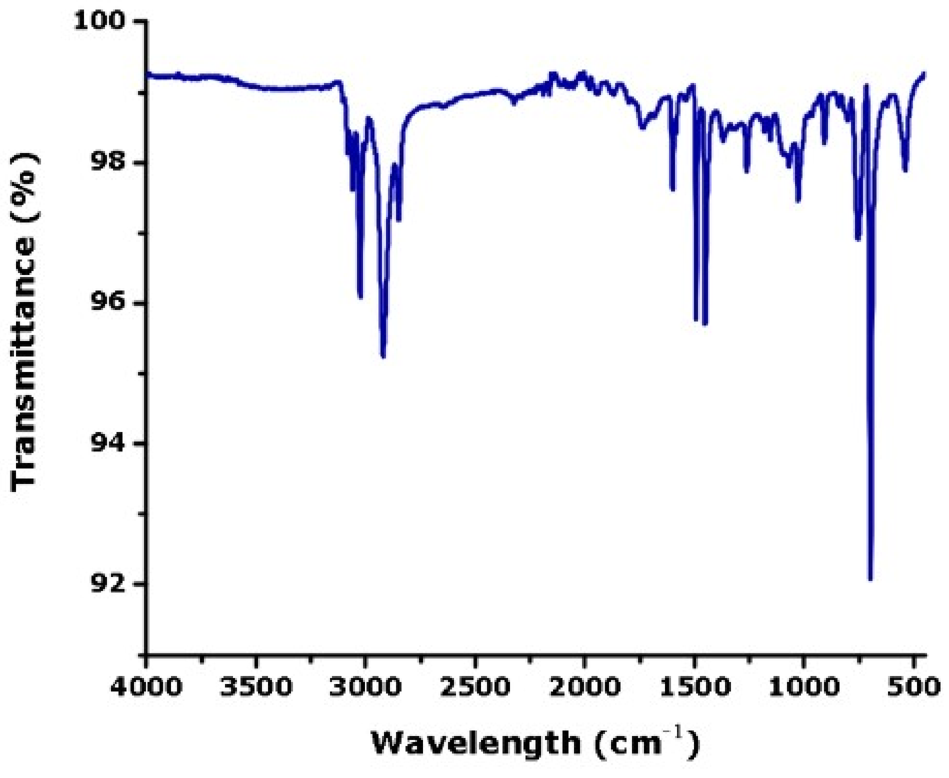

Figure S10: *FTIR spectrum of the oxidation of polystyrene using Mn(TACN).*

2.3.2 H_2O_2 addition 1 batch 1 mol% and 0.05 mol% catalyst loading (Table 1, Entries 3 & 4)

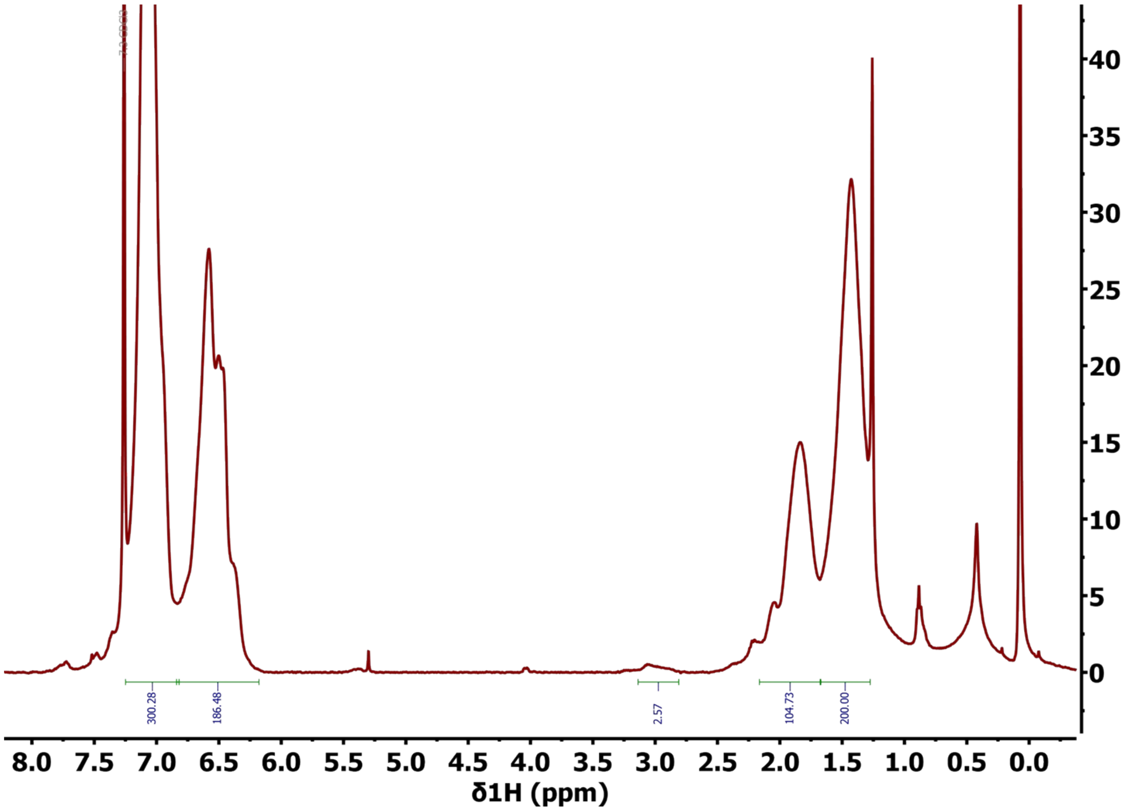

Figure S11: *^{1}H NMR spectrum of the oxidation of polystyrene using 1 mol% Mn(TACN) measured in chloroform-d at 25 °C.*

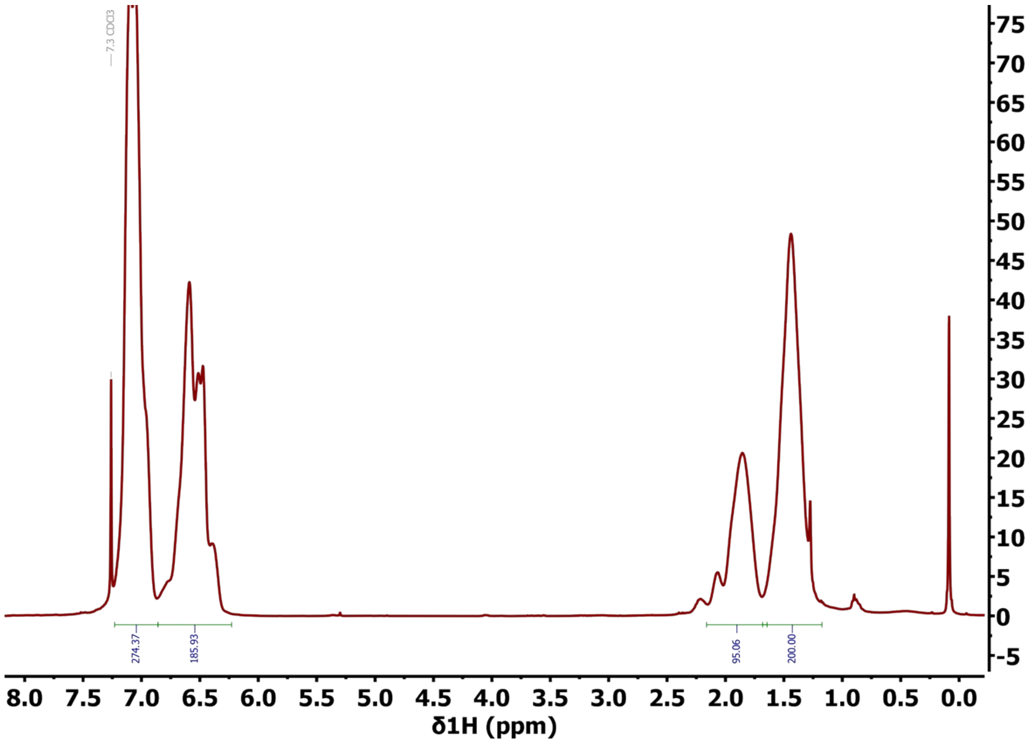

Figure S12: *^{1}H NMR spectrum of the oxidation of polystyrene using 0.05 mol% Mn(TACN) measured in chloroform-d at 25 °C.*

2.3.4 H_2O_2 addition portion wise (Table 1, Entries 6, 7 & 8)

A 50 mL flask was charged with polystyrene high M_w (480 mg, 4.61 mmol, 1 eq.) and dissolved in a mixture of DCM/MeCN (8 mL/2 mL) at 30 °C. The MnTACN (**1**) (187.7 mg, 0.23 mmol, 5 mol%) was added and the opening of the flask was covered using parafilm. 10 mL of 10% H_2O_2 was added portion wise (in 2 batches, 3 batches or 10 batches) to the red-coloured solution containing the catalyst and substrate using a syringe pump in 1 hour with 24 or 96 hour time intervals. During the addition, the colour changed to yellow, and the mixture was left to stir at 30 °C. Afterwards, the reaction mixture was washed with brine and DCM, the organics were dried over Na_2SO_4 and the solvent was evaporated using a rotary evaporator. The obtained off-white solids were thereafter dried in a vacuum oven for 24 hours.

Table 1 Entry 6

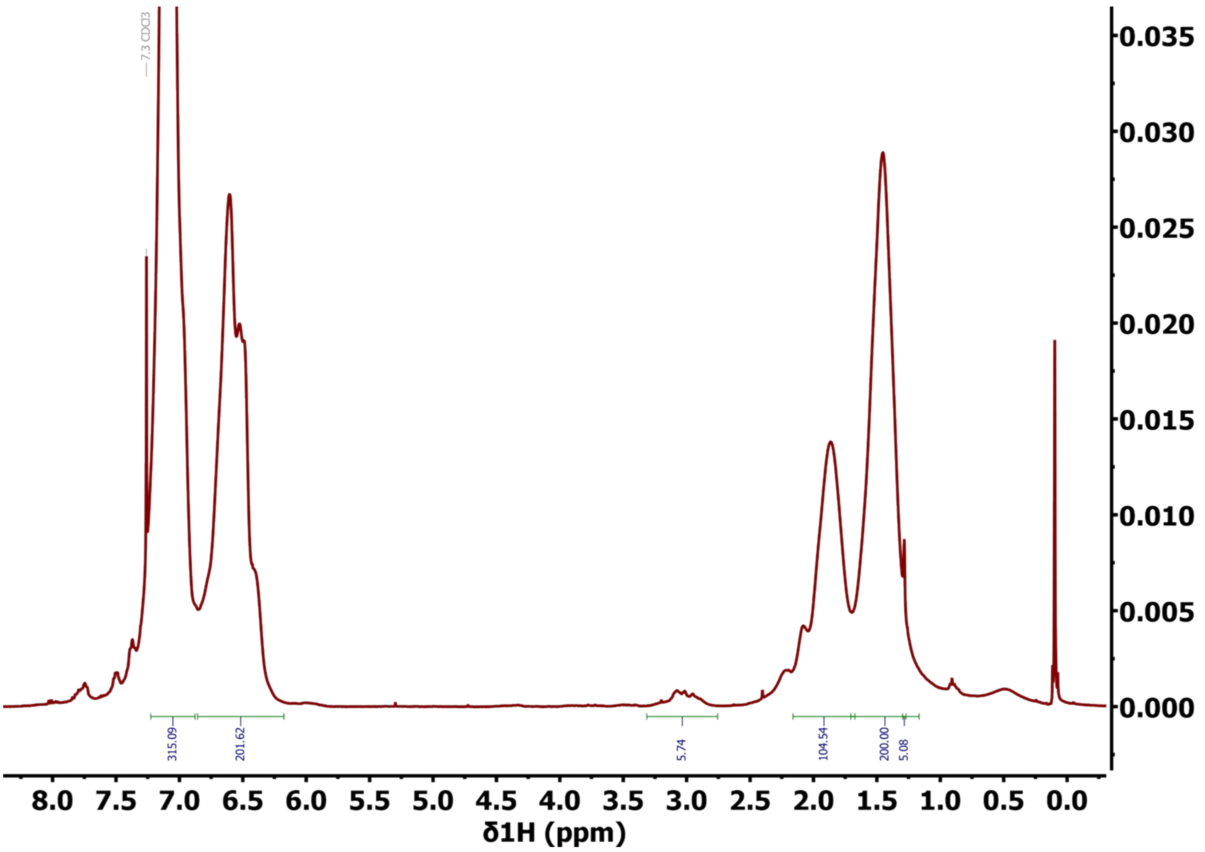

Figure S13: *^{1}H NMR spectrum of the oxidation of polystyrene using 5 mol% Mn(TACN), with 3x addition of H_2O_2 with a 24 h interval measured in chloroform-d at 25 °C.*

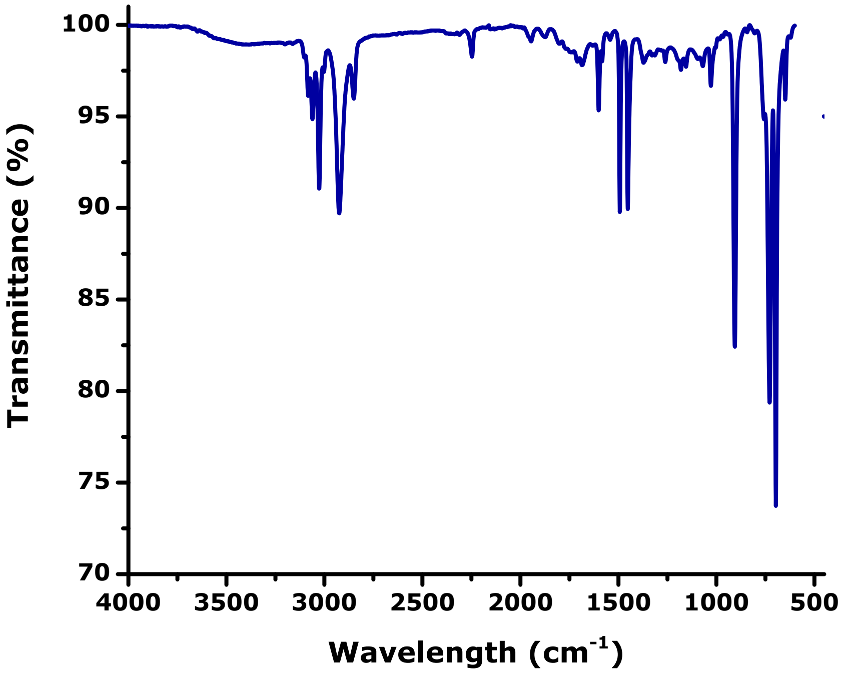

Figure S14: *FTIR spectrum of the oxidation of polystyrene using 5 mol% Mn(TACN) and 3x batch wise addition of H_2O_2 with a 24 hour interval.*

Table 1 Entry 7

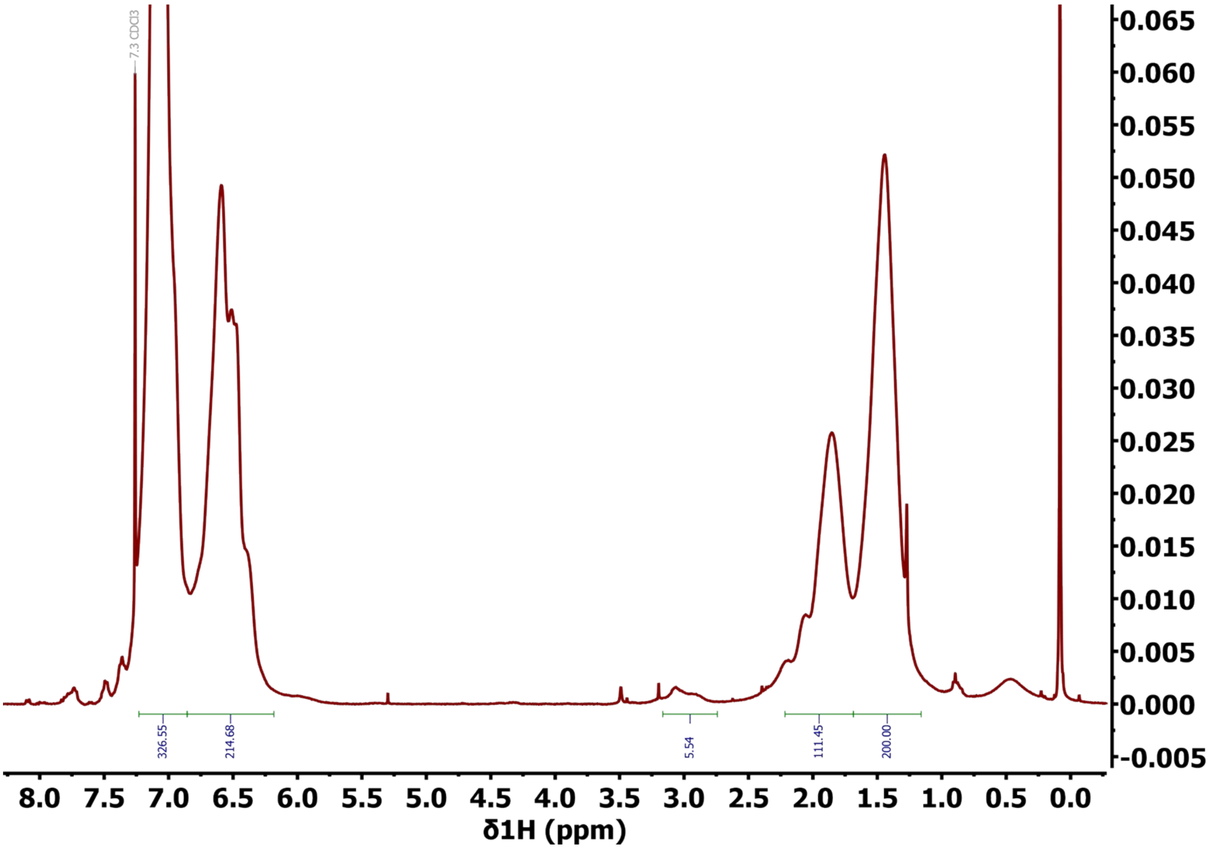

Figure S15: *^{1}H NMR spectrum of the oxidation of polystyrene using 5 mol% Mn(TACN), with 10x addition of H_2O_2 with a 24 h interval measured in chloroform-d at 25 °C.*

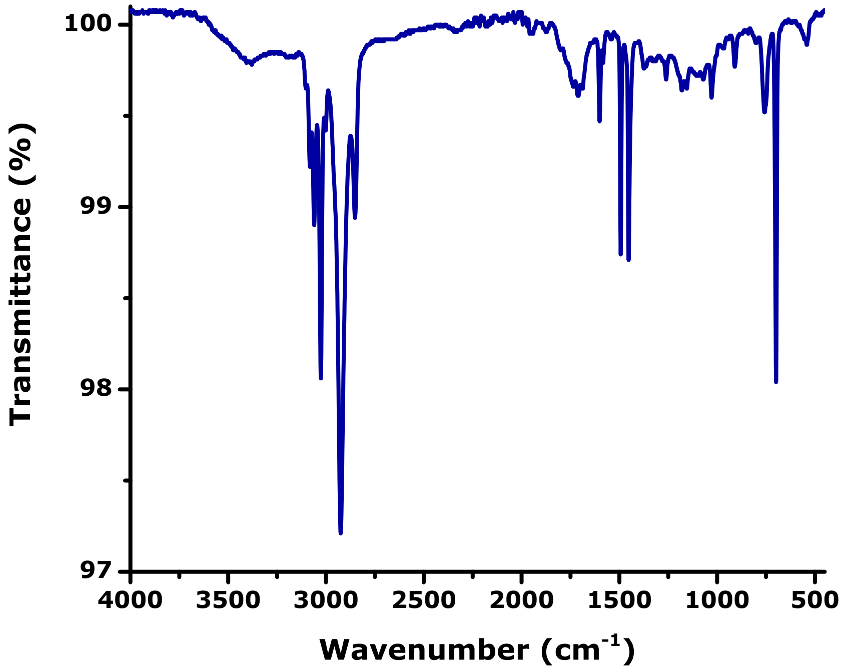

Figure S16: *FTIR spectrum of the oxidation of polystyrene using 5 mol% Mn(TACN) and 10x batch wise addition of H_2O_2 with a 24 hour interval.*

Table 1 Entry 8

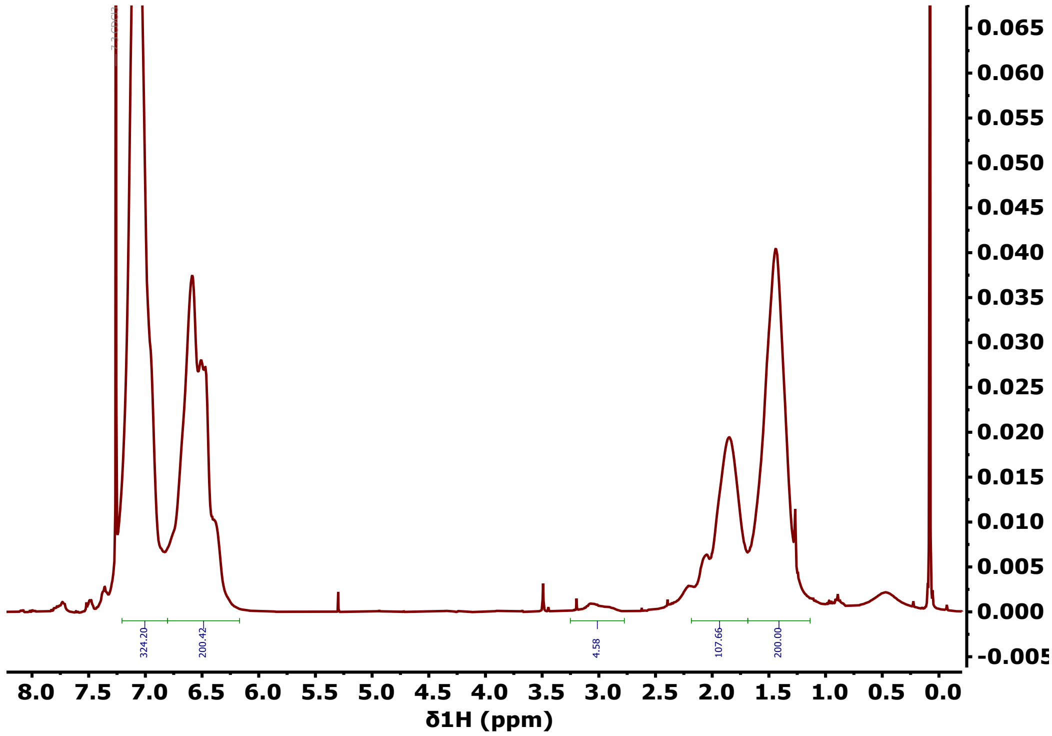

Figure S17: *^{1}H NMR spectrum of the oxidation of polystyrene using 5 mol% Mn(TACN), with 2x addition of H_2O_2 with a 96 h interval measured in chloroform-d at 25 °C.*

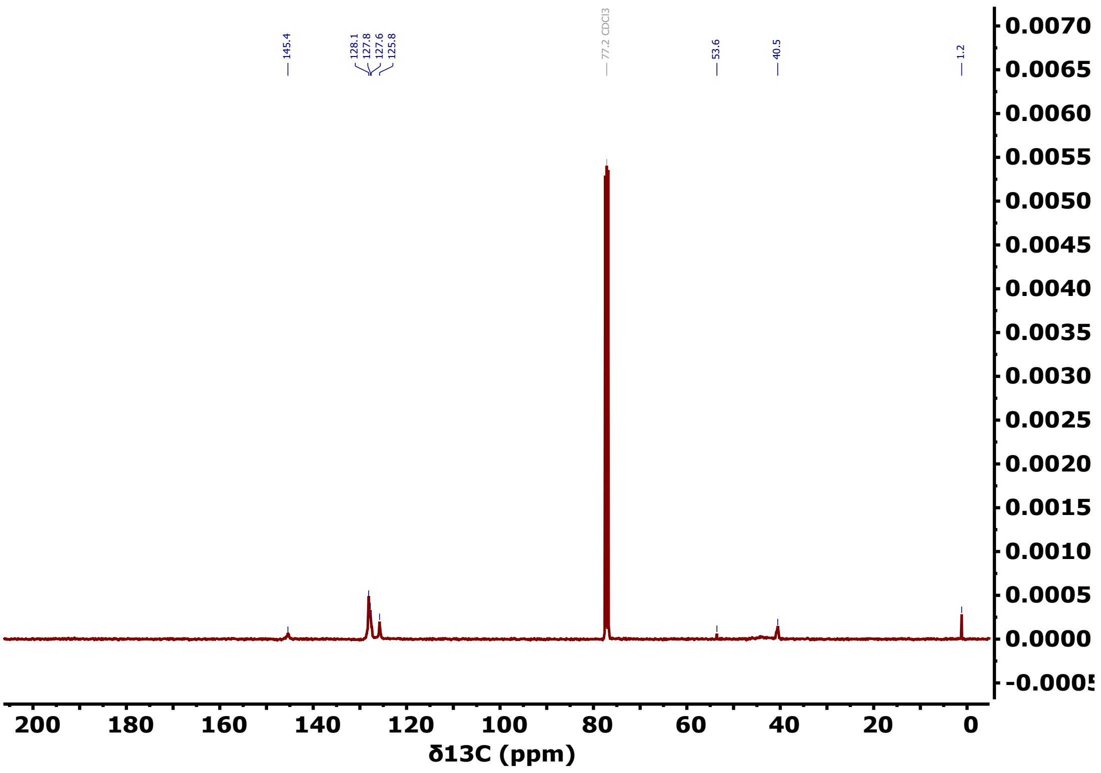

Figure S18: *^{13}C NMR spectrum of the oxidation of polystyrene using 5 mol% Mn(TACN), with 2x addition of H_2O_2 with a 96 h interval measured in chloroform-d at 25 °C.*

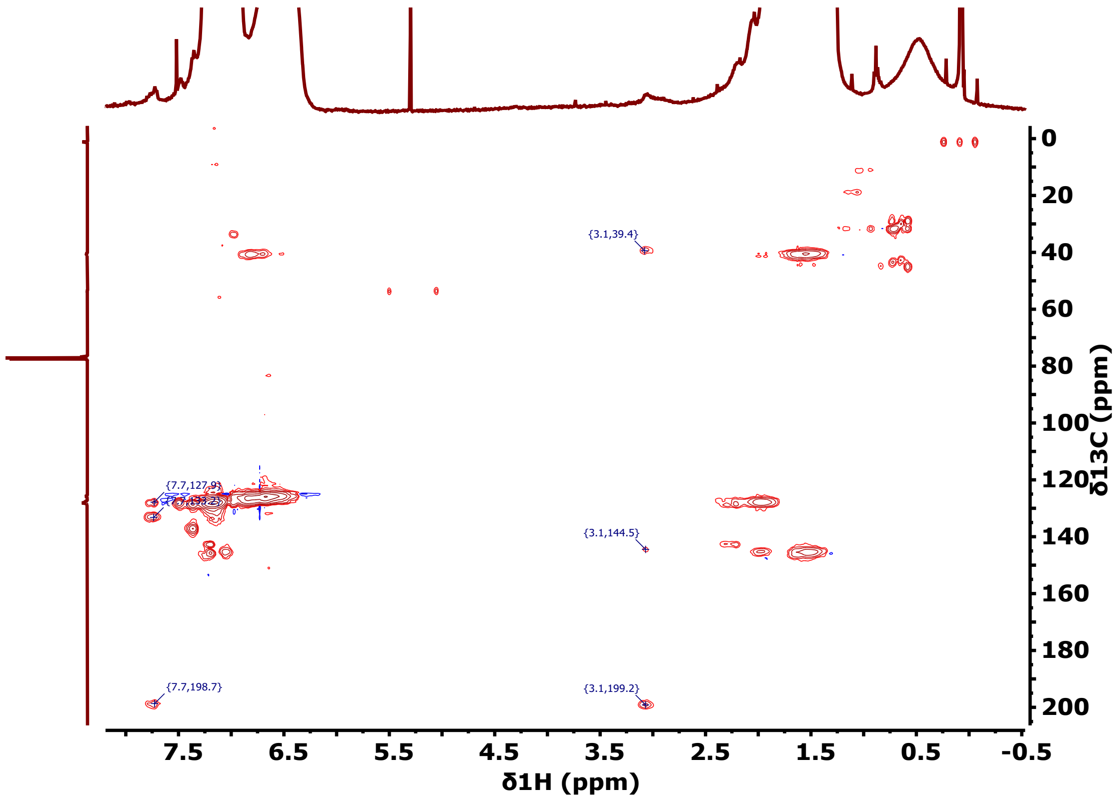

Figure S19: *$^{1}H^{13}C$ HMBC NMR spectrum of the oxidation of polystyrene using 5 mol% Mn(TACN), with 2x addition of H_2O_2 with a 96 h interval measured in chloroform-d at 25 °C.*

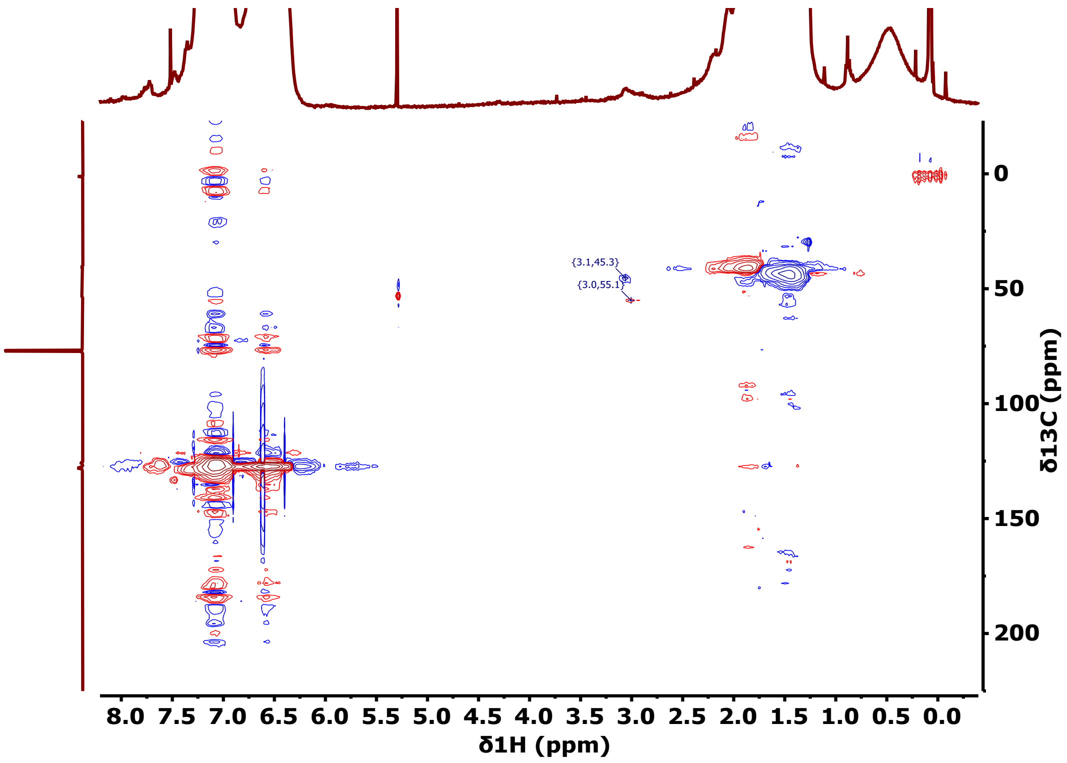

Figure S20: *^{1}H^{13}C HSQC NMR spectrum of the oxidation of polystyrene using 5 mol% Mn(TACN), with 2x addition of H_2O_2 with a 96 h interval measured in chloroform-d at 25 °C.*

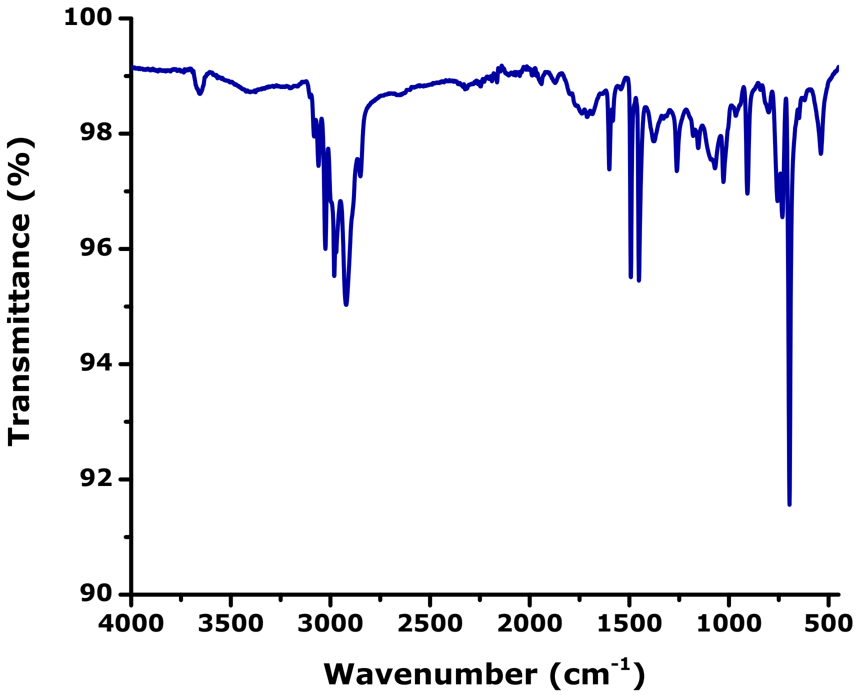

Figure S21: *FTIR spectrum of the oxidation of polystyrene using 5 mol% Mn(TACN) and 2x batch wise addition of H_2O_2 with a 96 hour interval.*

Table 1, Entry 9

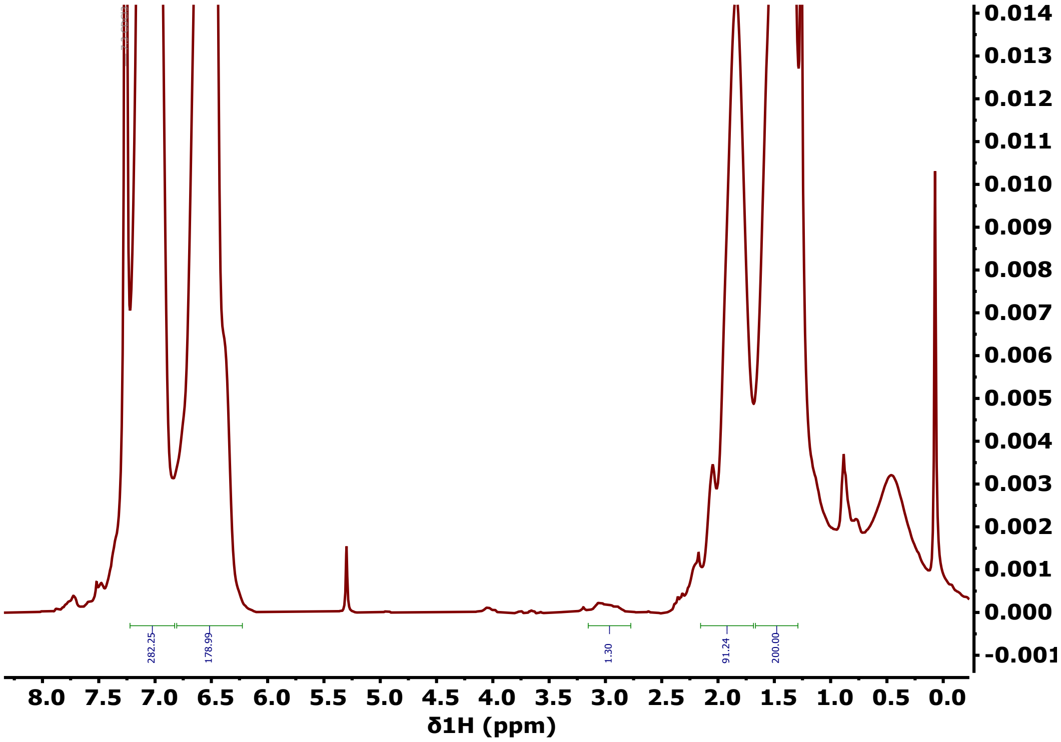

Figure S22: *^{1}H NMR spectrum of the oxidation of polystyrene using Mn(TACN) measured in chloroform-d at 25 °C.*

2.3.5 H_2O_2 addition portion wise (Table 1, Entry 10)

A 50 mL flask was charged with polystyrene low M_w (480 mg, 4.61 mmol, 1 eq.) and dissolved in a mixture of DCM/MeCN (8 mL/2 mL) at 30 °C. The MnTACN (**1**) (187.7 mg, 0.23 mmol, 5 mol%) was added and the opening of the flask was covered using parafilm. 10 mL of 10% H_2O_2 was added portion wise (in 2 batches) to the red-coloured solution containing the catalyst and substrate using a syringe pump in 1 hour with a 96 hour time interval. During the addition, the colour changed to yellow, and the mixture was left to stir at 30 °C. Afterwards, the reaction mixture was washed with brine and DCM, the organics were dried over Na_2SO_4 and the solvent was evaporated using a rotary evaporator. The obtained off-white solids were thereafter dried in a vacuum oven for 24 hours.

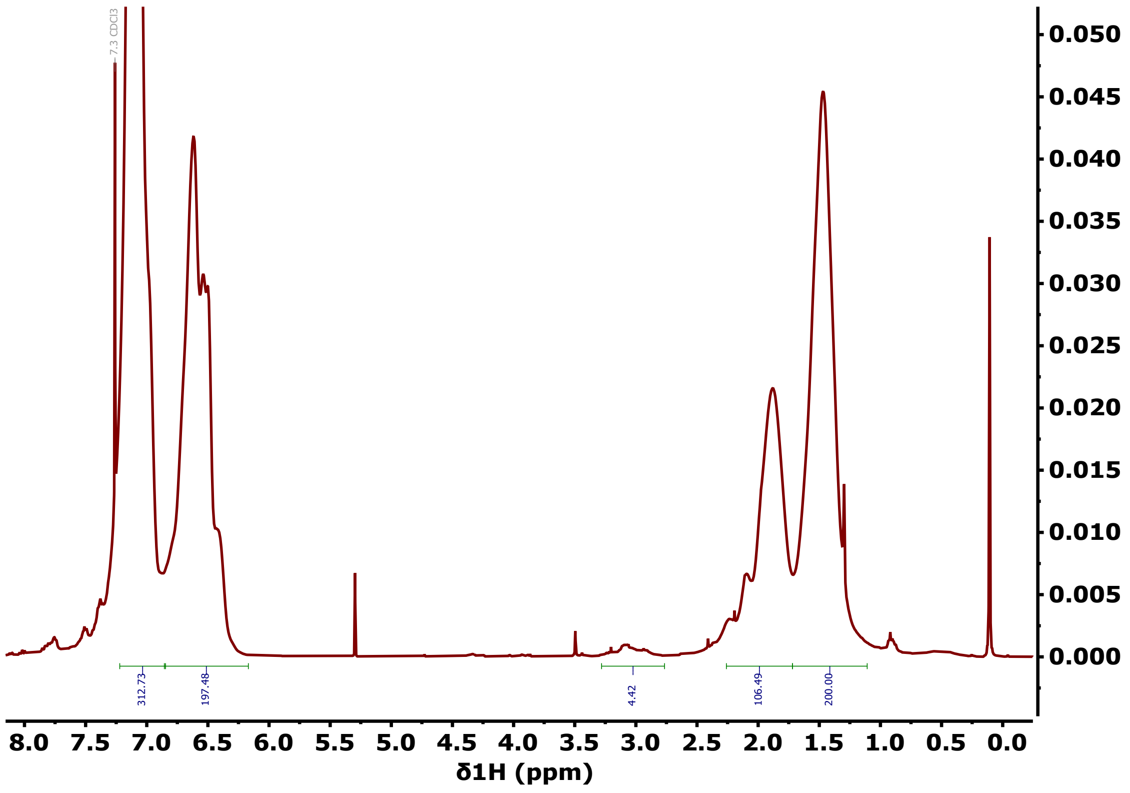

Figure S23: *^{1}H NMR spectrum of the oxidation of polystyrene using 5 mol% Mn(TACN), with 2x addition of H_2O_2 with a 96 h interval measured in chloroform-d at 25 °C.*

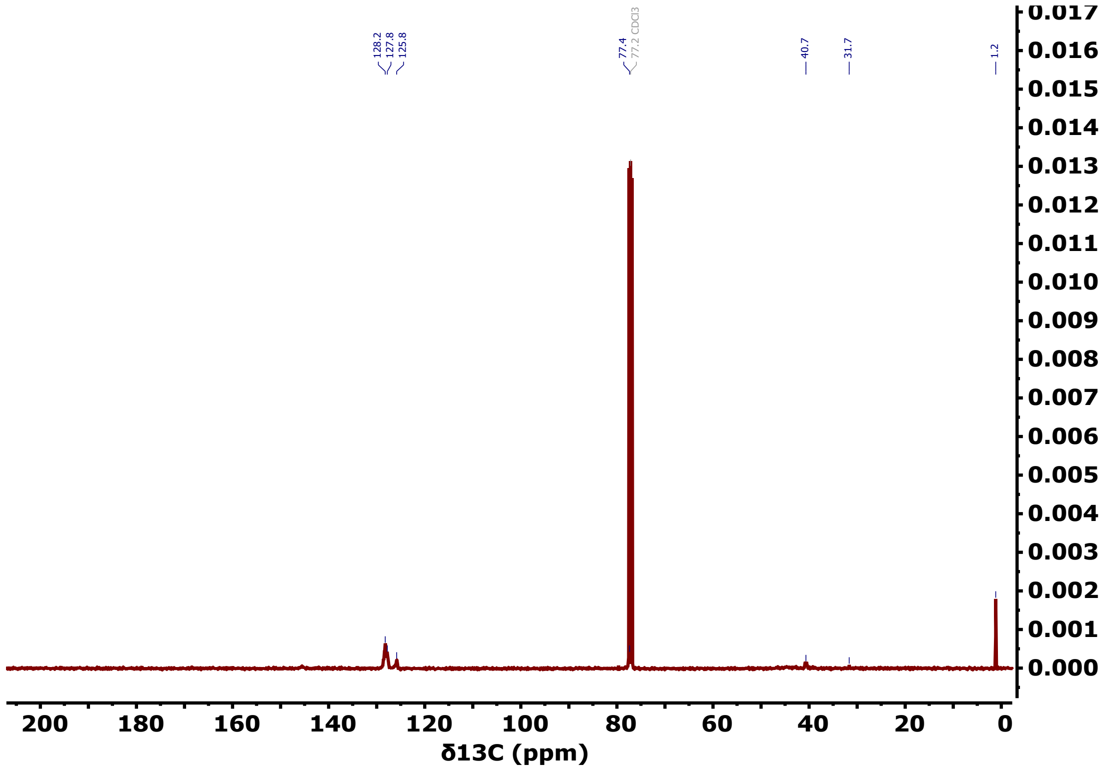

Figure S24: *^{13}C NMR spectrum of the oxidation of polystyrene using 5 mol% Mn(TACN), with 2x addition of H_2O_2 with a 96 h interval measured in chloroform-d at 25 °C.*

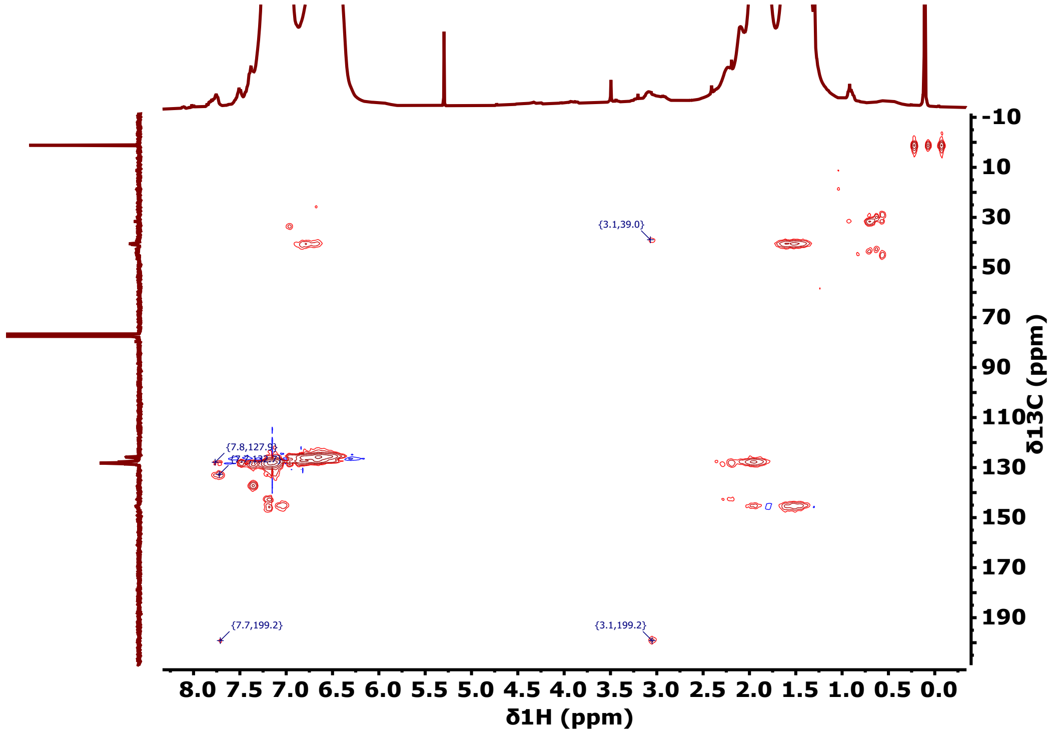

Figure S25: *$^{1}H^{13}C$ HMBC NMR spectrum of the oxidation of polystyrene using 5 mol% Mn(TACN), with 2x addition of H_2O_2 with a 96 h interval measured in chloroform-d at 25 °C.*

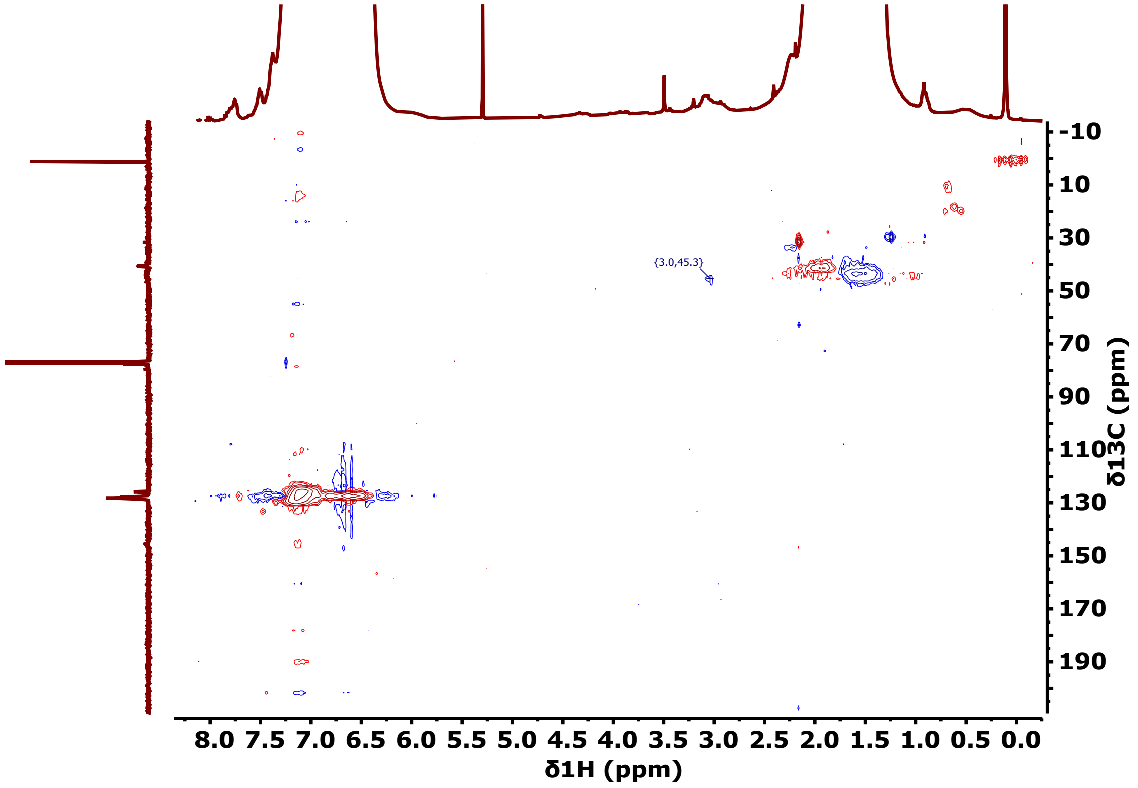

Figure S26: *$^{1}H^{13}C$ HSQC NMR spectrum of the oxidation of polystyrene using 5 mol% Mn(TACN), with 2x addition of H_2O_2 with a 96 h interval measured in chloroform-d at 25 °C.*

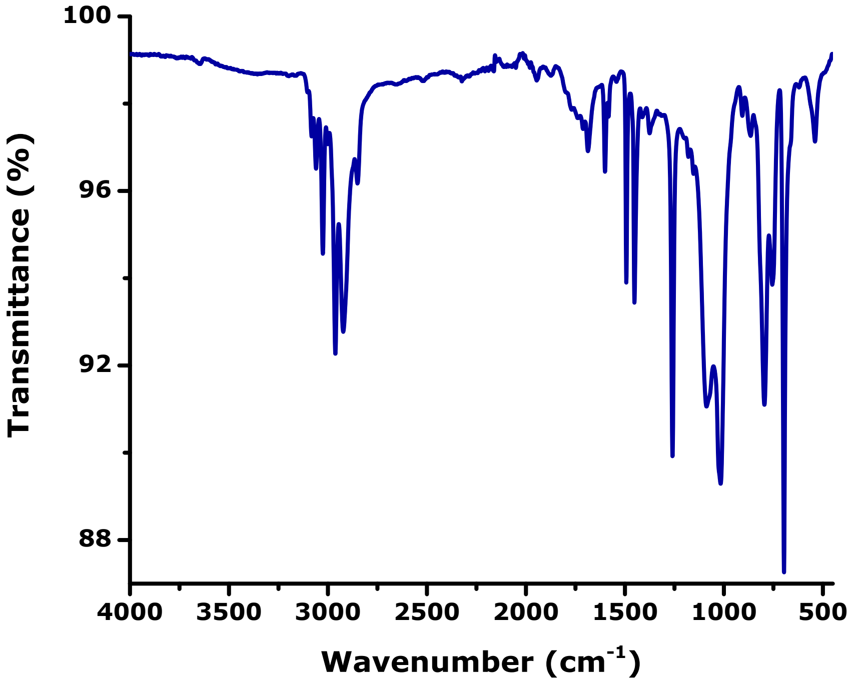

Figure S27: *FTIR spectrum of the oxidation of polystyrene using 5 mol% Mn(TACN) and 2x batch wise addition of H_2O_2 with a 96 hour interval.*

2.4 Catalytic oxidation reaction of PBD8020 with tri-oxo Mn(TACN) (1)

2.4.1 H_2O_2 addition in 1 batch (Table 4, Entry 3)

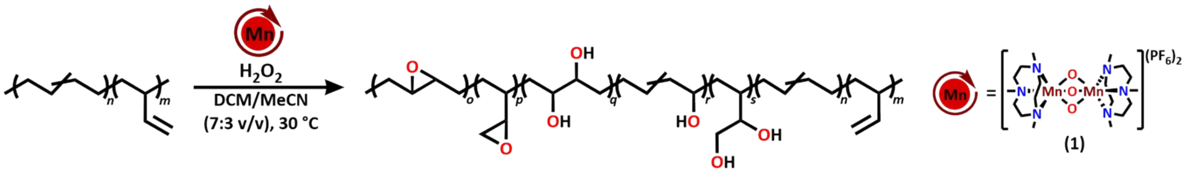

A 50 mL flask was charged with polybutadiene (1000 mg, 18.7 mmol, 1 eq.) and dissolved in a mixture of DCM/MeCN (8 mL/2 mL) at 30 °C. The MnTACN (**1**) (3.7 mg, 0.005 mmol, 0.03 mol%) was added and the opening of the flask was covered using parafilm. 10 mL of 10% H_2O_2 was added to the red-coloured solution containing the catalyst and substrate using a syringe pump in 1 hour. During the addition, the colour changed to yellow, and the mixture was left to stir for 72 hours at 30 °C. Afterwards, the reaction mixture was washed with brine and DCM, the organics were dried over Na_2SO_4 and the solvent was evaporated using a rotary evaporator. The obtained off-white solids were thereafter dried in a vacuum oven for 24 hours. (see table below for functionalisation degree and yields)

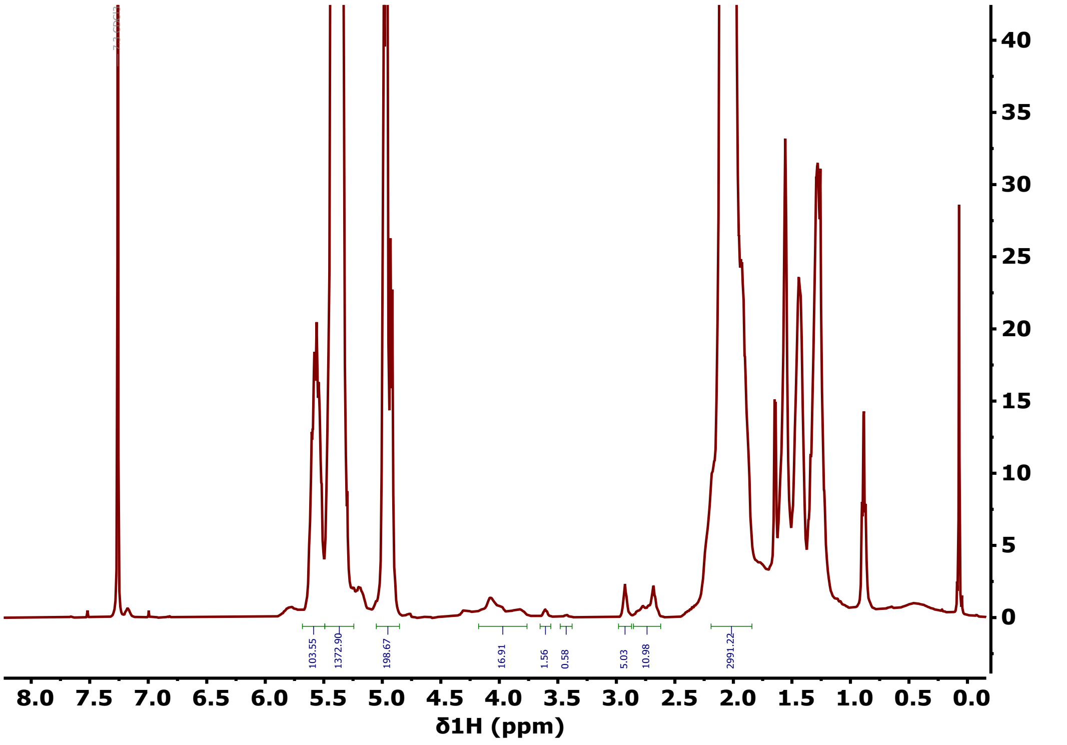

Figure S28: *^{1}H NMR spectrum of the oxidation of polybutadiene using 0.03 mol% Mn(TACN), measured in chloroform-d at 25 °C.*

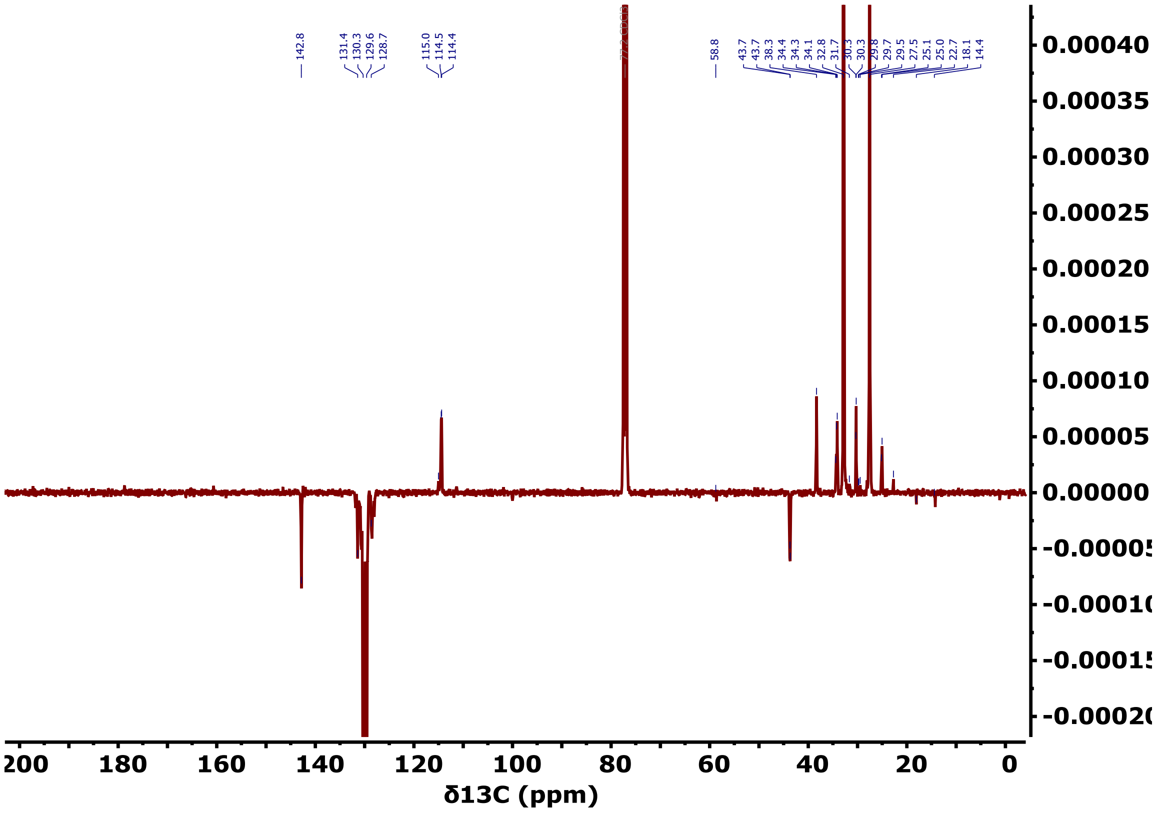

Figure S29: *^{13}C APT NMR spectrum of the oxidation of polybutadiene using 0.03 mol% Mn(TACN), measured in chloroform-d at 25 °C.*

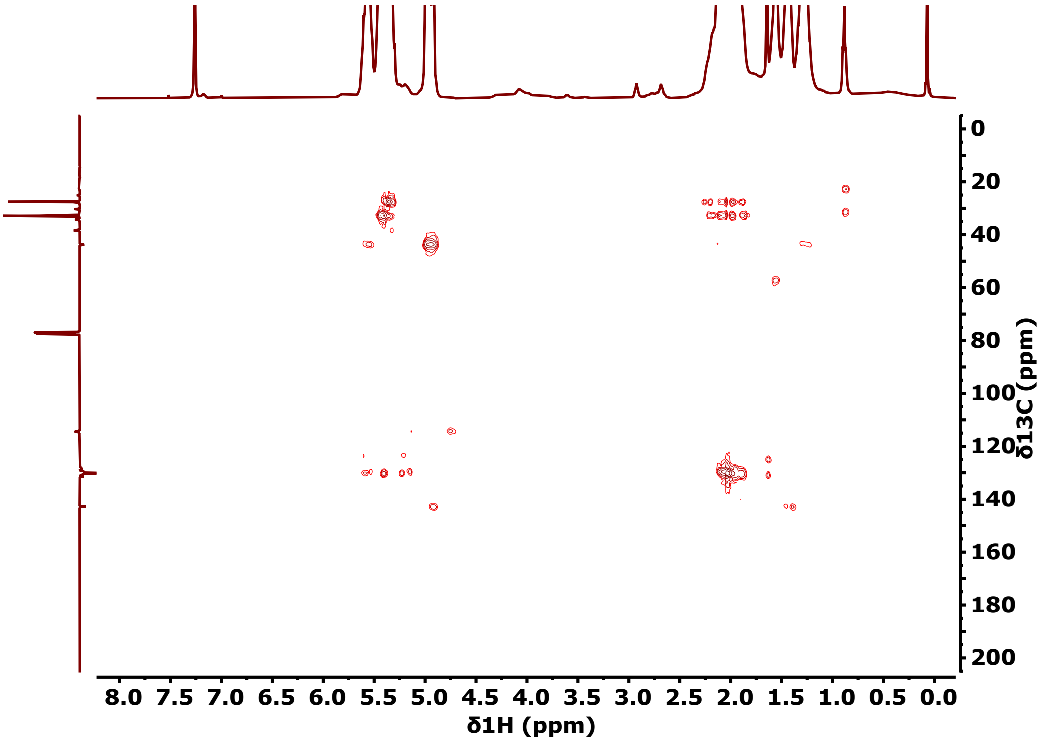

Figure S30: *$^{1}H^{13}C$ HMBC NMR spectrum of the oxidation of polybutadiene using 0.03 mol% Mn(TACN), measured in chloroform-d at 25 °C.*

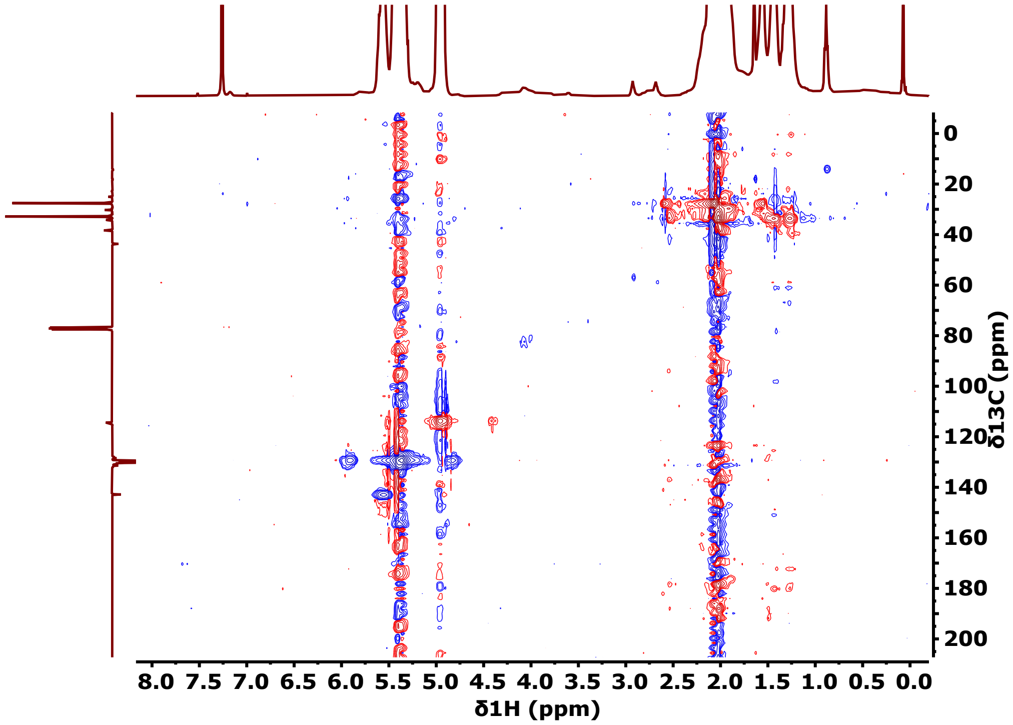

Figure S31: *$^{1}H^{13}C$ HSQC NMR spectrum of the oxidation of polybutadiene using 0.03 mol% Mn(TACN), measured in chloroform-d at 25 °C.*

2.4.2 H_2O_2 addition portion wise (Table 4, Entries 4 & 5)

A 50 mL flask was charged with polybutadiene (1000 mg, 18.7 mmol, 1 eq.) and dissolved in a mixture of DCM/MeCN (8 mL/2 mL) at 30 °C. The MnTACN (**1**) (3.7 mg, 0.005 mmol, 0.03 mol%) was added and the opening of the flask was covered using parafilm. 10 mL of 10% H_2O_2 was added portion wise (in 2 batches or 3 batches) to the red-coloured solution containing the catalyst and substrate using a syringe pump in 1 hour with 24 or 96 hour time intervals. During the addition, the colour changed to yellow, and the mixture was left to stir at 30 °C. Afterwards, the reaction mixture was washed with brine and DCM, the organics were dried over Na_2SO_4 and the solvent was evaporated using a rotary evaporator. The obtained off-white solids were thereafter dried in a vacuum oven for 24 hours.

Table 2, Entry 4

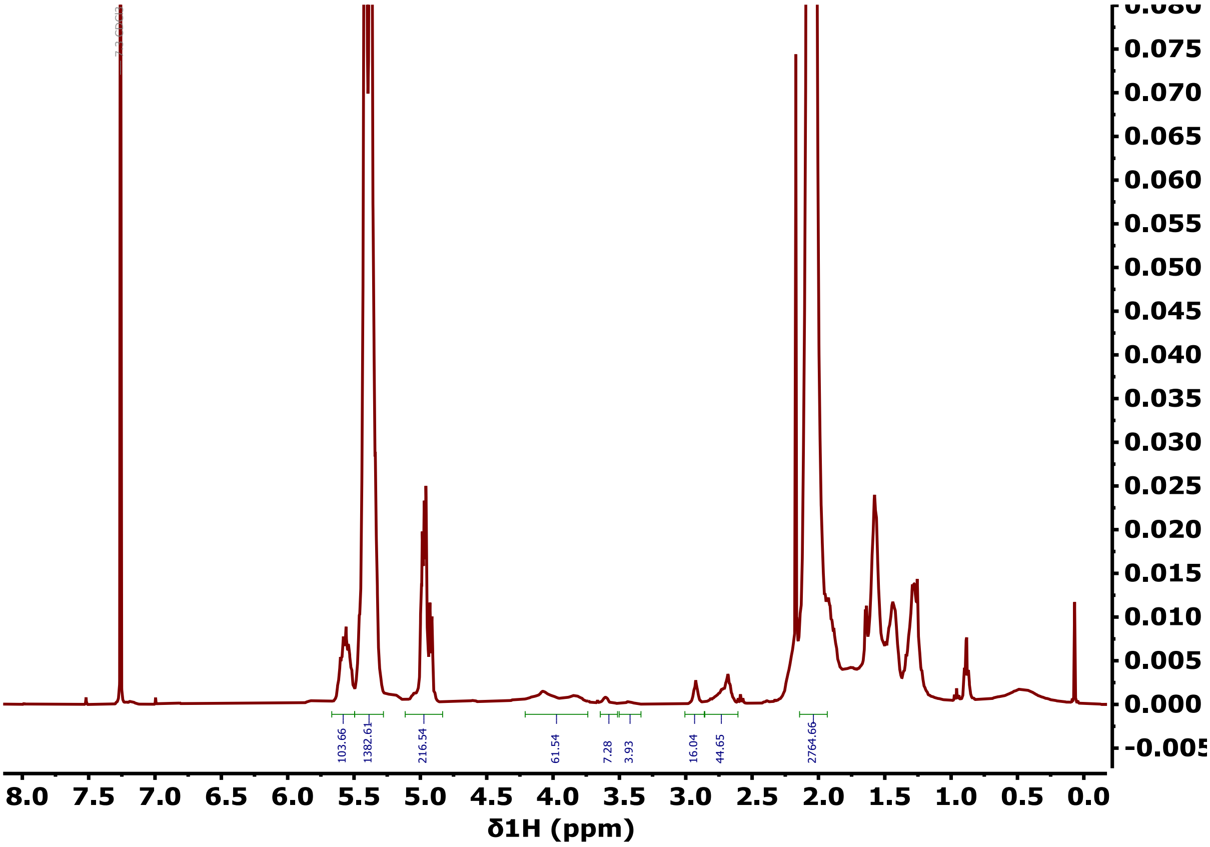

Figure S32: *^1H NMR spectrum of the oxidation of polybutadiene using 0.03 mol% Mn(TACN), with 3x batchwise addition of H_2O_2 with 24 h time intervals, measured in chloroform-d at 25 °C.*

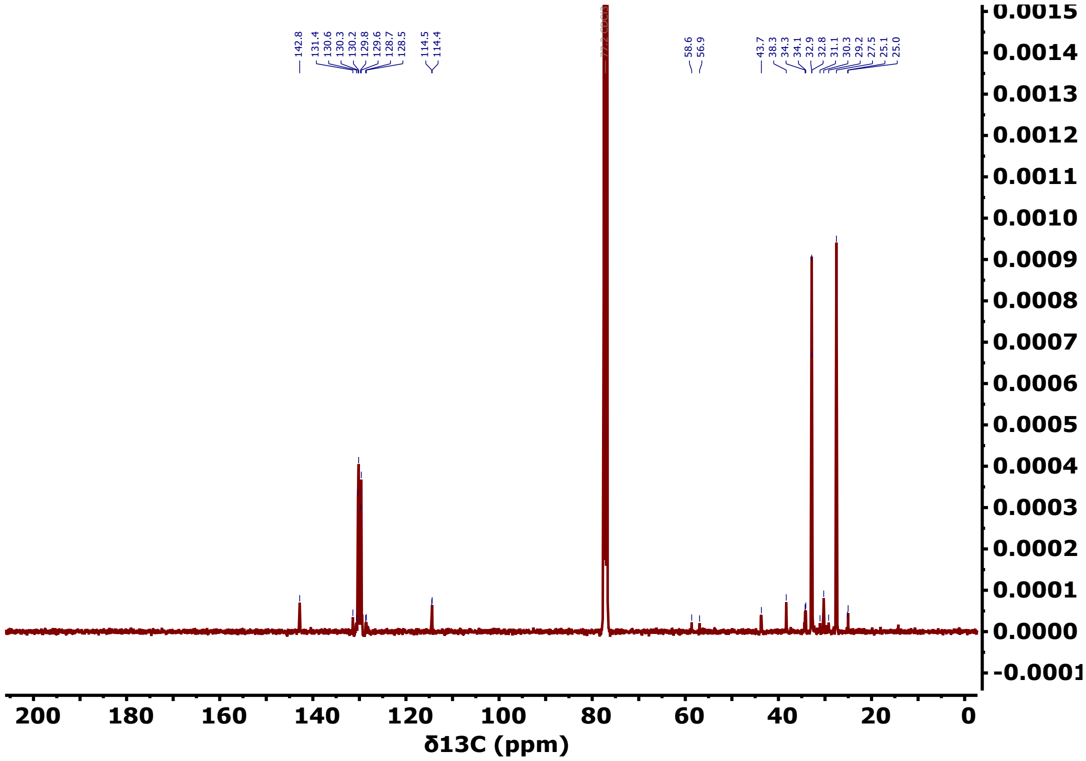

Figure S33: *^{13}C NMR spectrum of the oxidation of polybutadiene using 0.03 mol% Mn(TACN), with 3x batchwise addition of H_2O_2 with 24 h time intervals, measured in chloroform-d at 25 °C.*

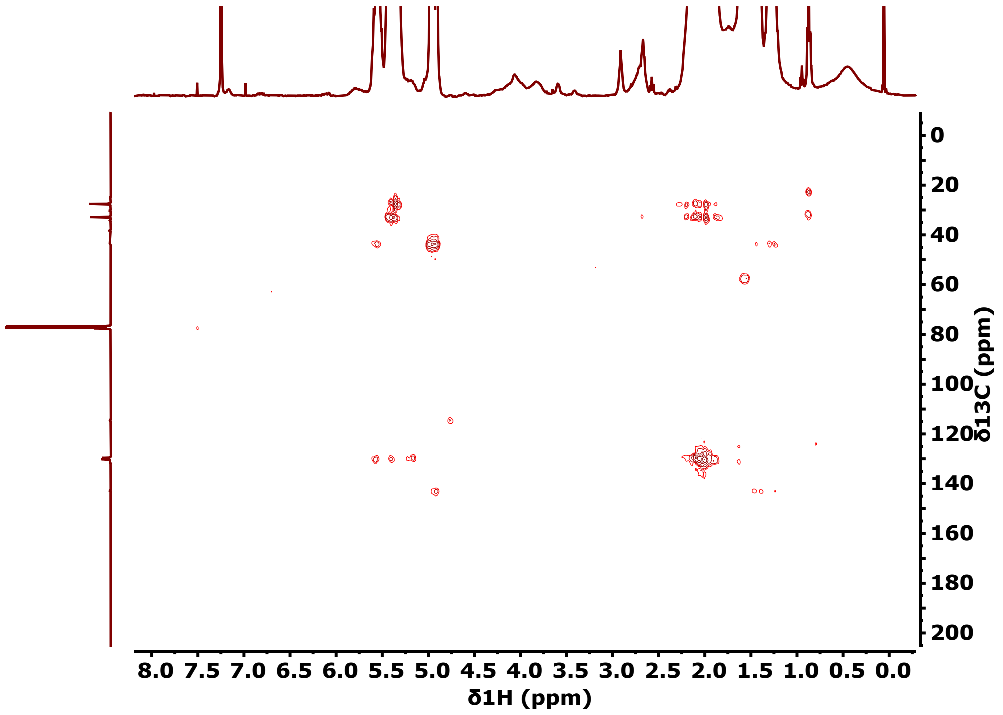

Figure S34: *$^1H^{13}C$ HMBC NMR spectrum of the oxidation of polybutadiene using 0.03 mol% Mn(TACN), with 3x batchwise addition of H_2O_2 with 24 h time intervals, measured in chloroform-d at 25 °C.*

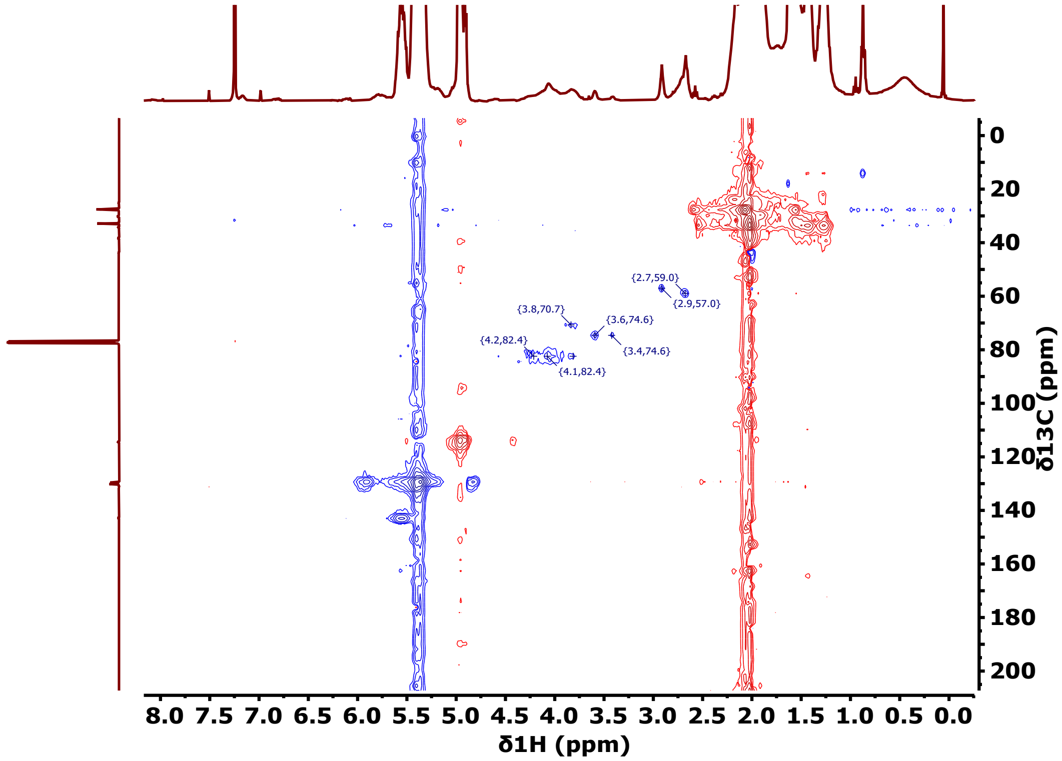

Figure S35: *^{1}H^{13}C HSQC NMR spectrum of the oxidation of polybutadiene using 0.03 mol% Mn(TACN), with 3x batchwise addition of H_2O_2 with 24 h time intervals, measured in chloroform-d at 25 °C.*

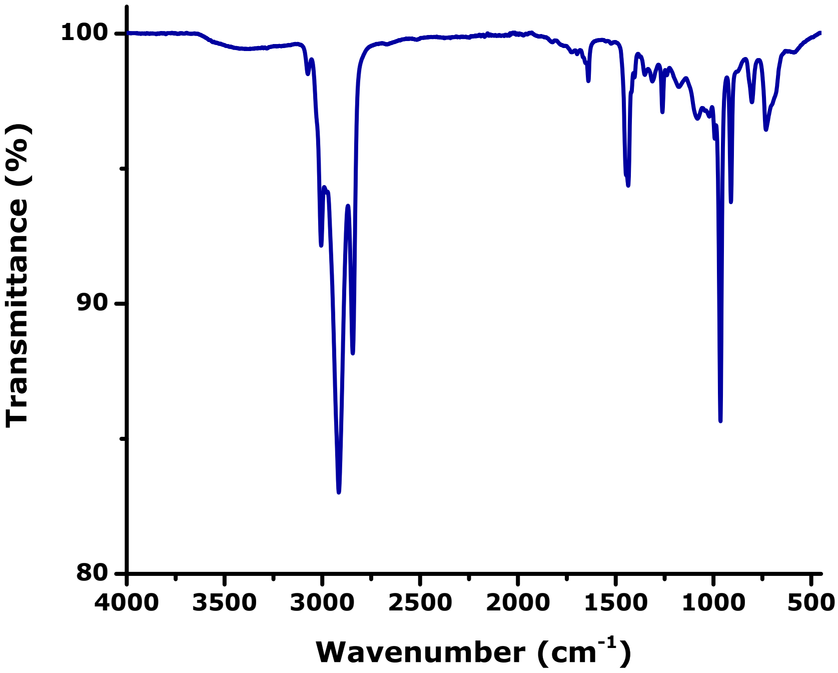

Figure S36: *FTIR spectrum of the oxidation of polybutadiene using 0.03 mol% Mn(TACN), with 3x batchwise addition of H_2O_2 with 24 h time intervals, measured in chloroform-d at 25 °C.*

Table 2, Entry 5

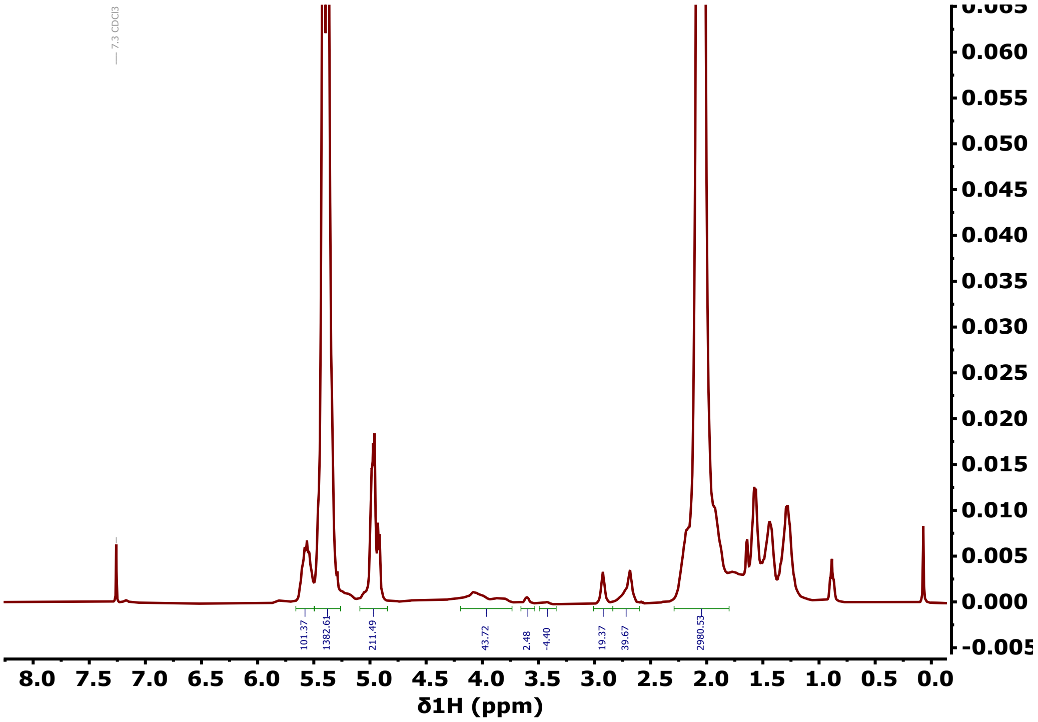

Figure S37: *^{1}H NMR spectrum of the oxidation of polybutadiene using 0.03 mol% Mn(TACN), with 2x batchwise addition of H_2O_2 with 96 h time intervals, measured in chloroform-d at 25 °C.*

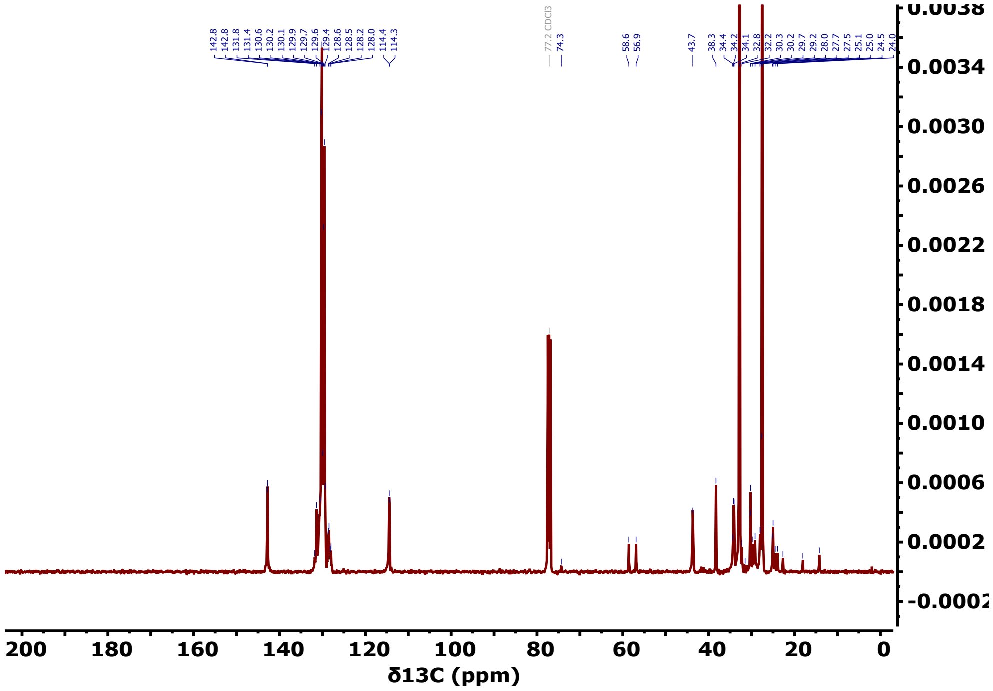

Figure S38: *^{13}C NMR spectrum of the oxidation of polybutadiene using 0.03 mol% Mn(TACN), with 2x batchwise addition of H_2O_2 with 96 h time intervals, measured in chloroform-d at 25 °C.*

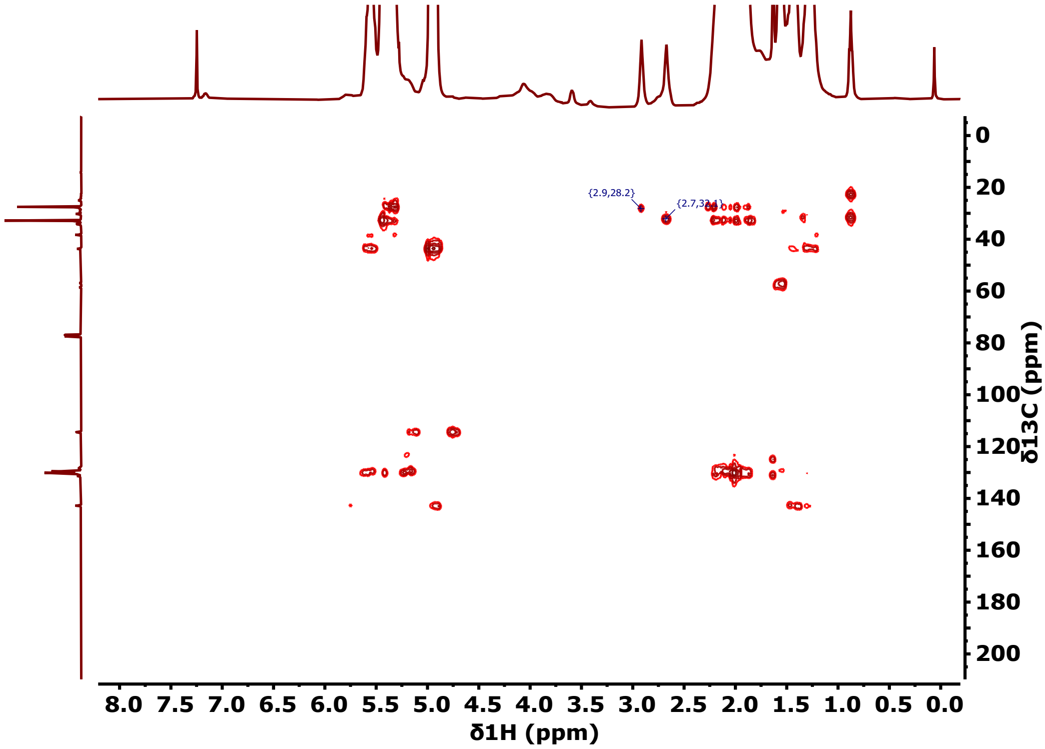

Figure S39: *$^{1}H^{13}C$ HMBC NMR spectrum of the oxidation of polybutadiene using 0.03 mol% Mn(TACN), with 2x batchwise addition of H_2O_2 with 96 h time intervals, measured in chloroform-d at 25 °C.*

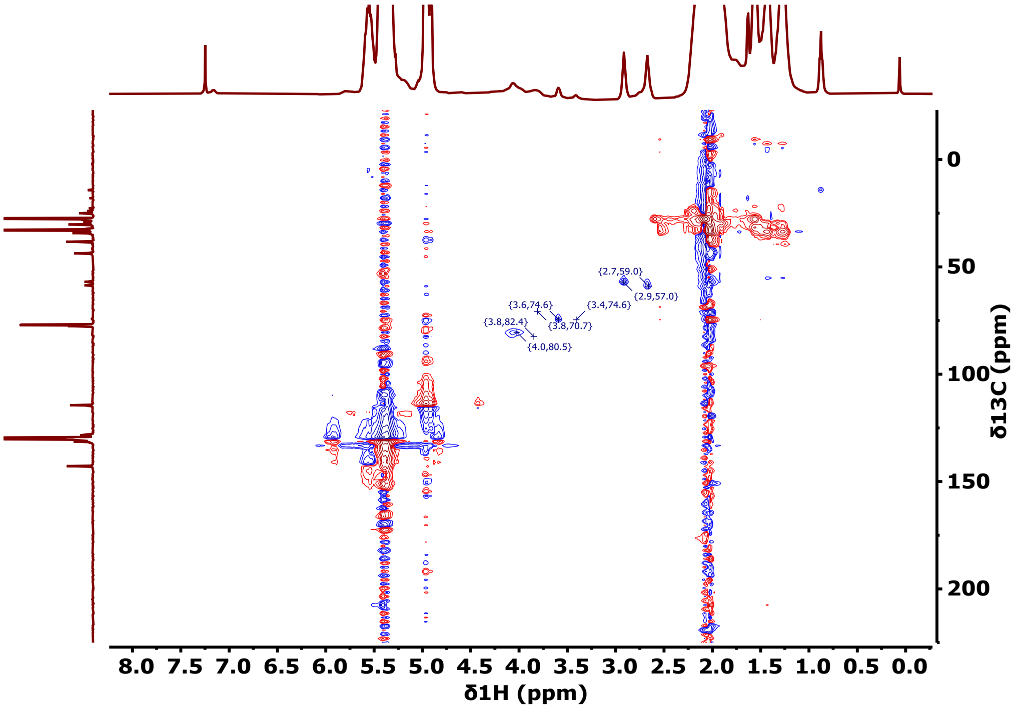

Figure S40: *$^{1}H^{13}C$ HSQC NMR spectrum of the oxidation of polybutadiene using 0.03 mol% Mn(TACN), with 2x batchwise addition of H_2O_2 with 96 h time intervals, measured in chloroform-d at 25 °C.*

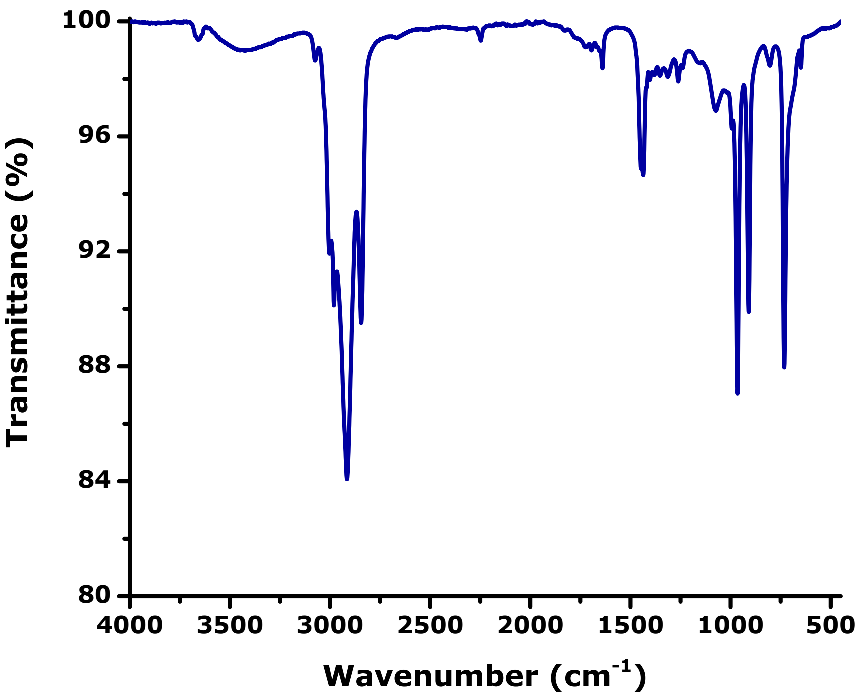

Figure S41: *FTIR spectrum of the oxidation of polybutadiene using 0.03 mol% Mn(TACN), with 2x batchwise addition of H_2O_2 with 96 h time intervals, measured in chloroform-d at 25 °C.*

2.5 Catalytic oxidation reaction of SBS with tri-oxo Mn(TACN) (1)

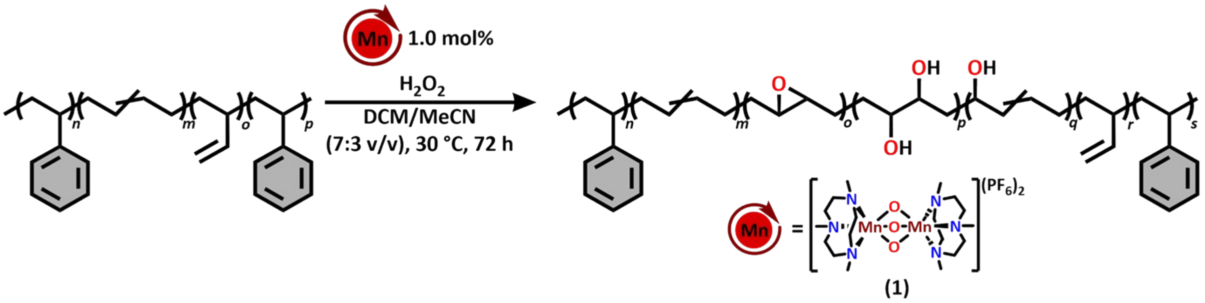

A 50 mL flask was charged with styrene-butadiene-styrene block-co-polymer (1000 mg, 15.8 mmol, 1 eq.) and dissolved in a mixture of DCM/MeCN (8 mL/2 mL) at 30 °C. The MnTACN (**1**) (128.0 mg, 0.158 mmol, 1 mol%) was added and the opening of the flask was covered using parafilm. 10 mL of 10% H_2O_2 was added to the red-coloured solution containing the catalyst and substrate using a syringe pump in 1 hour. During the addition, the colour changed to yellow, and the mixture was left to stir for 72 hours at 30 °C. Afterwards, the reaction mixture was washed with brine and DCM, the organics were dried over Na_2SO_4 and the solvent was evaporated using a rotary evaporator. The obtained off-white solids were thereafter dried in a vacuum oven for 24 hours (FG_{Total} = 11.4%, Yield recovered material = 91%)

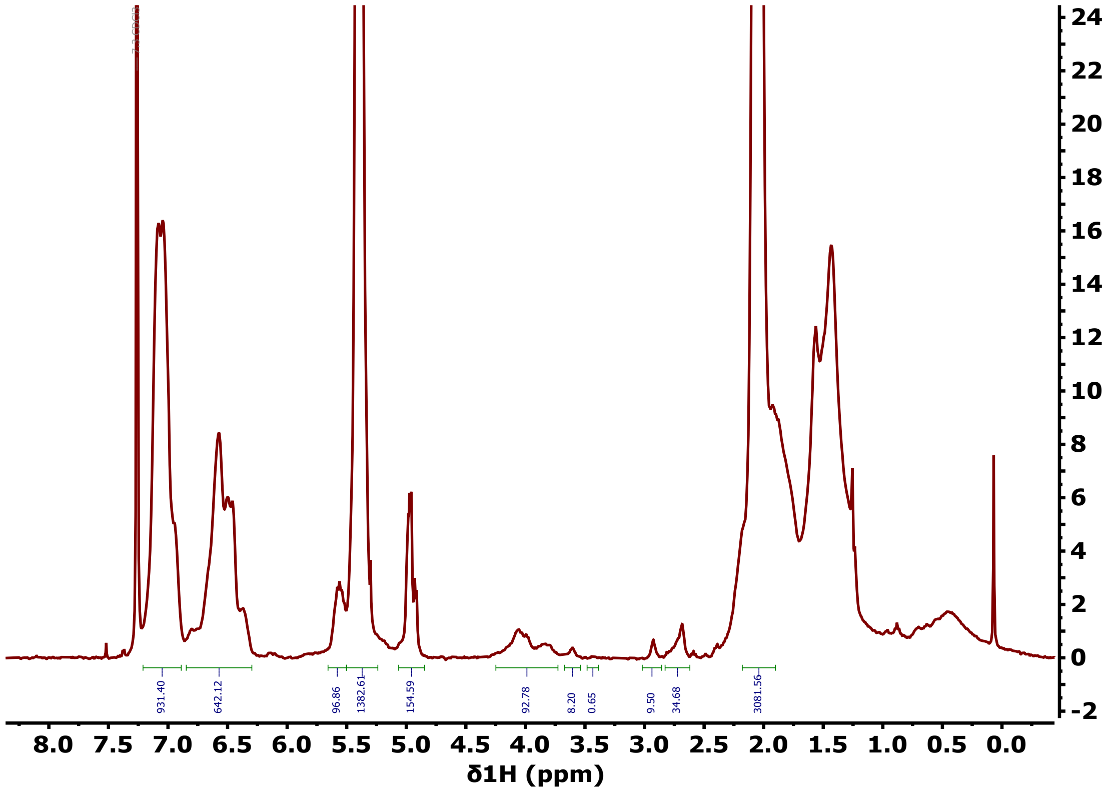

Figure S42: *^{1}H NMR spectrum of the scaled up oxidation of styrene-butadiene-styrene block-co-polymer using 1 mol% Mn(TACN), measured in chloroform-d at 25 °C.*

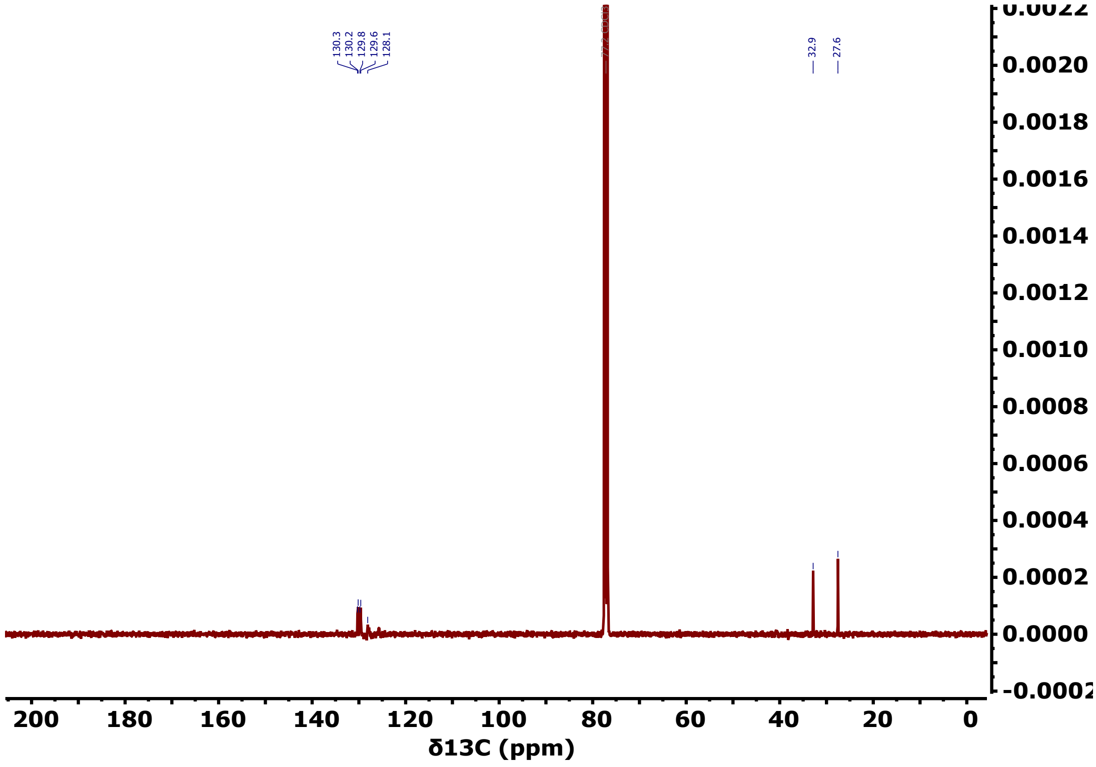

Figure S43: *^{13}C NMR spectrum of the scaled up oxidation of styrene-butadiene-styrene block-co-polymer using 1 mol% Mn(TACN), measured in chloroform-d at 25 °C.*

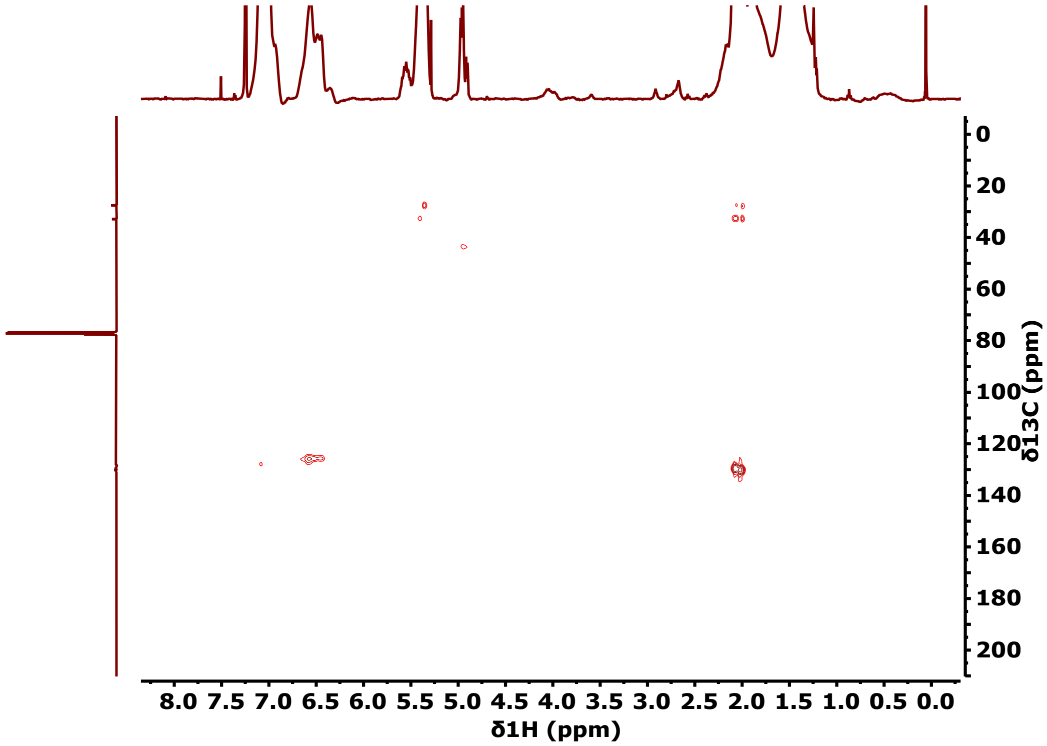

Figure S44: *$^1H^{13}C$ HMBC NMR spectrum of the scaled up oxidation of styrene-butadiene-styrene block-co-polymer using 1 mol% Mn(TACN), measured in chloroform-d at 25 °C.*

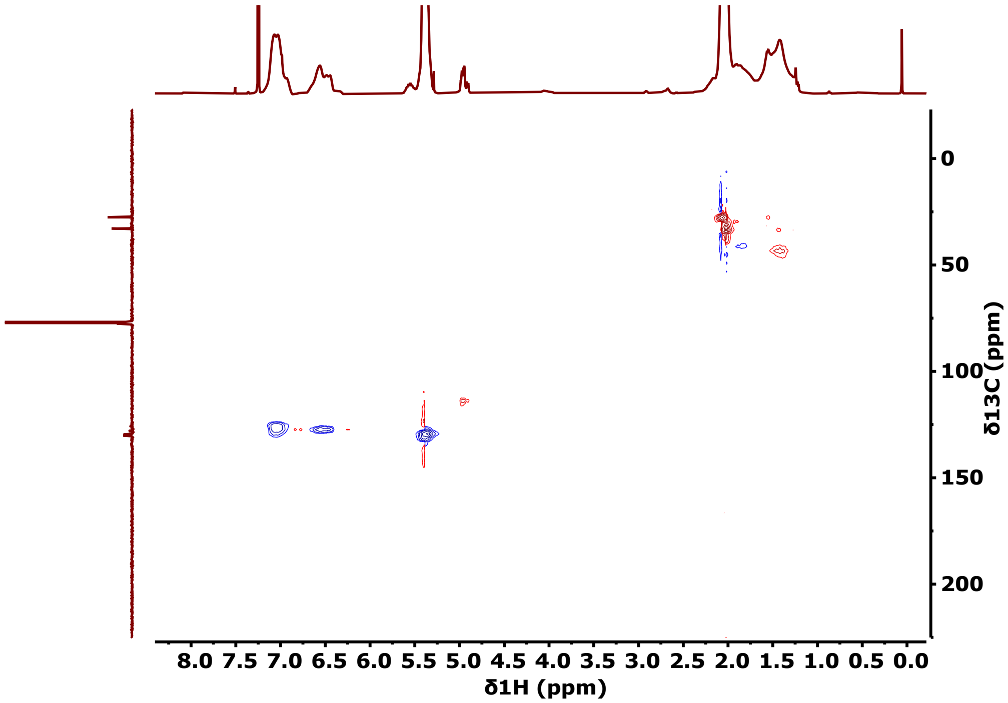

Figure S45: *$^1H^{13}C$ HSQC NMR spectrum of the scaled up oxidation of styrene-butadiene-styrene block-co-polymer using 1 mol% Mn(TACN), measured in chloroform-d at 25 °C.*

2.6 Epoxide opening to diol on oxypolybutadiene

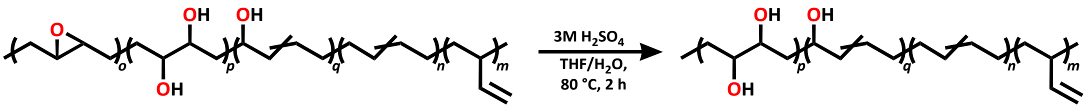

To a flask equipped with a stir bar and condenser was added 116.1 mg oxidised polybutadiene in 10 mL THF. To this mixture was added 10 mL 3M H_2SO_4 in H_2O (30 mmol), whereafter the mixture was refluxed at 80 °C for 2 hours. After cooling down the mixture was washed with 10 mL each of DCM and brine, whereafter the organics were dried over Na_2SO_4. After evaporation of DCM the diol functionalized polybutadiene was isolated as a clear wax (90.5% yield).

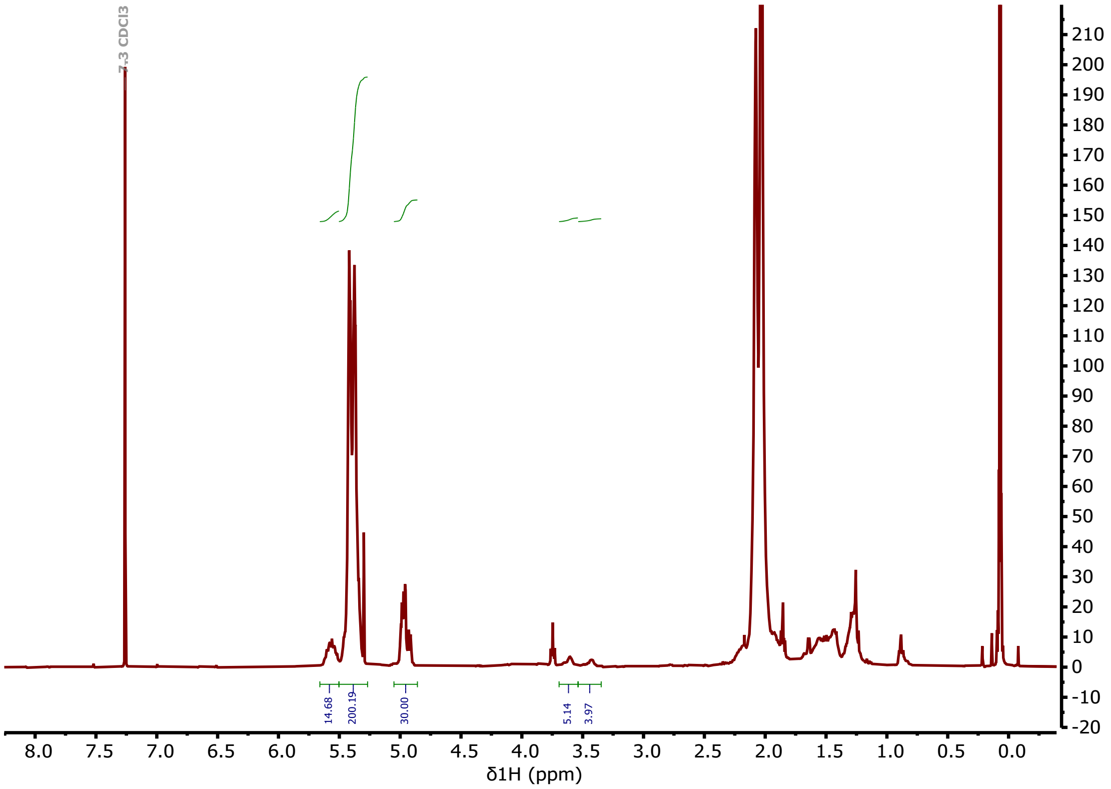

Figure S46: *^{1}H NMR spectrum of the fully diol functionalised polybutadiene, measured in chloroform-d at 25 °C.*

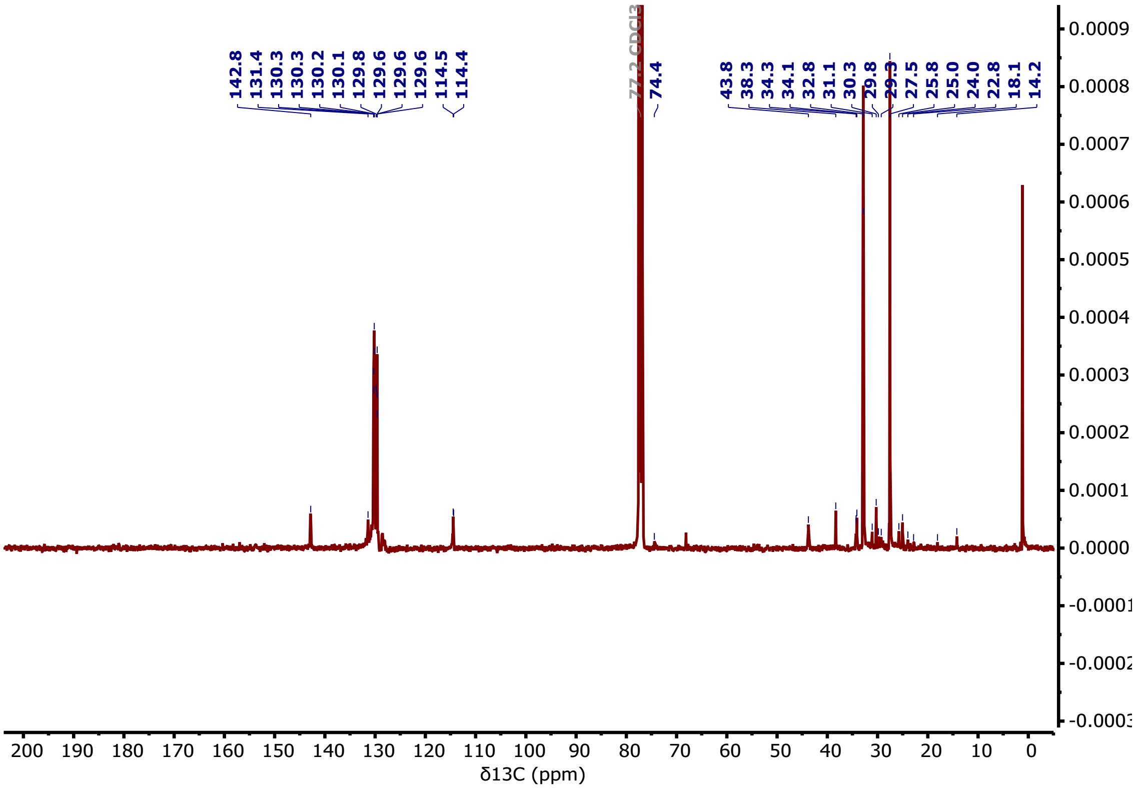

Figure S47: *^{13}C NMR spectrum of the fully diol functionalised polybutadiene, measured in chloroform-d at 25 °C.*

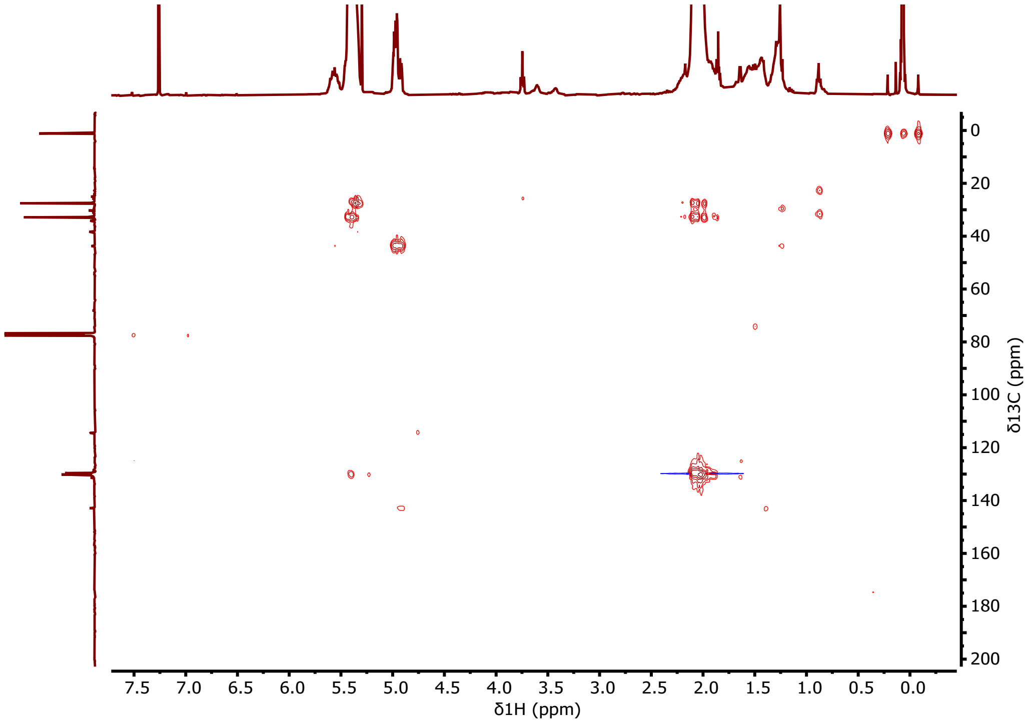

Figure S48: *$^{1}H^{13}C$ HMBC NMR spectrum of the fully diol functionalised polybutadiene, measured in chloroform-d at 25 °C.*

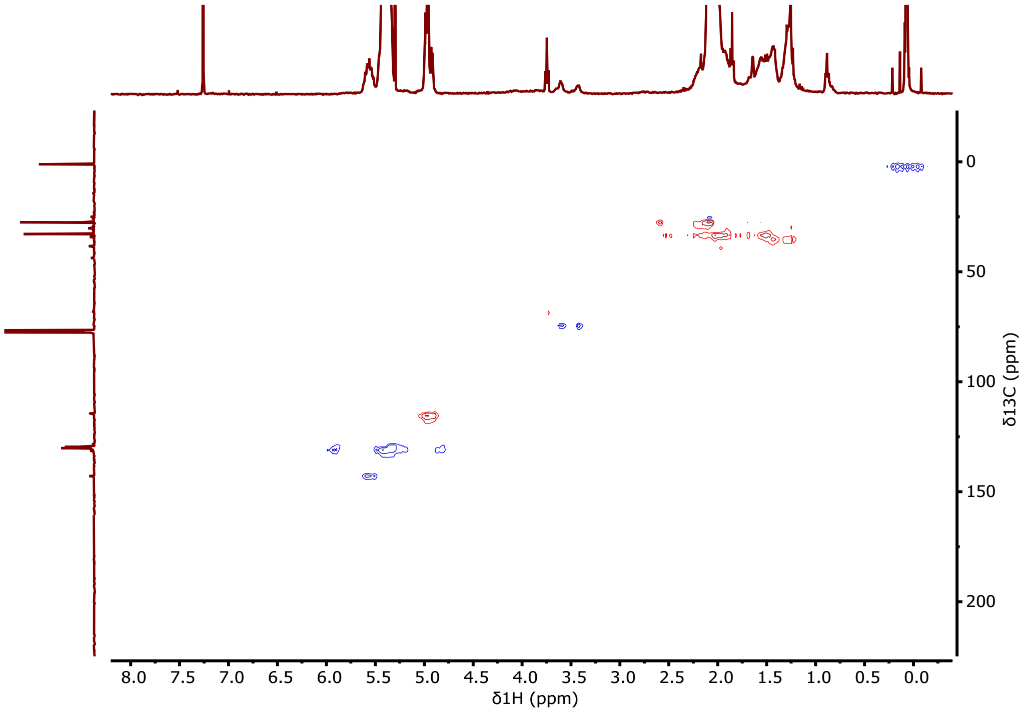

Figure S49: *^{1}H^{13}C HSQC NMR spectrum of the fully diol functionalised polybutadiene, measured in chloroform-d at 25 °C.*

2.7 Oxidative cleavage of the diol motives on oxypolybutadiene

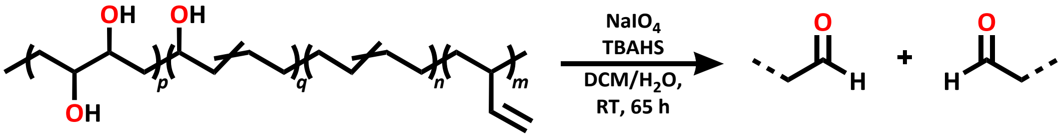

To a flask equipped with a stir bar was added 46.7 mg diol functionalized polybutadiene in 5 mL DCM. To this mixture was added 57.3 mg (0.27 mmol) $NaIO_4$ and 11.1 mg (0.03 mmol) tetrabutylammonium hydrogen sulphate in 5 mL H_2O. The resulting mixture was vigorously stirred at ambient temperature for 72 hours, whereafter the mixture was washed several times with DCM and brine. The organics were subsequently dried over Na_2SO_4 and the solvent was evaporated to isolate the oxidatively cleaved aldehyde species as a clear liquid/wax (85.0% yield). For the GPC measurement the solvent was not evaporated prior to the measurement to ensure no low molecular weight fragments would evaporate.

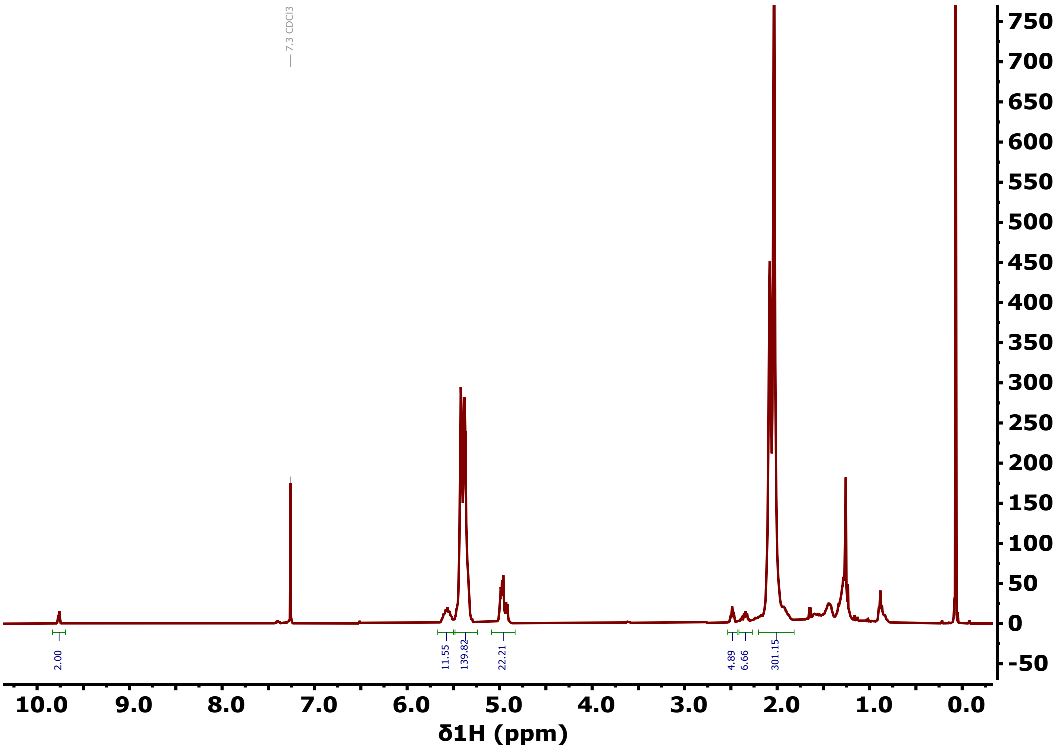

Figure S50: *^{1}H NMR spectrum of the cleaved diol functionalised polybutadiene to aldehydes, measured in chloroform-d at 25 °C.*

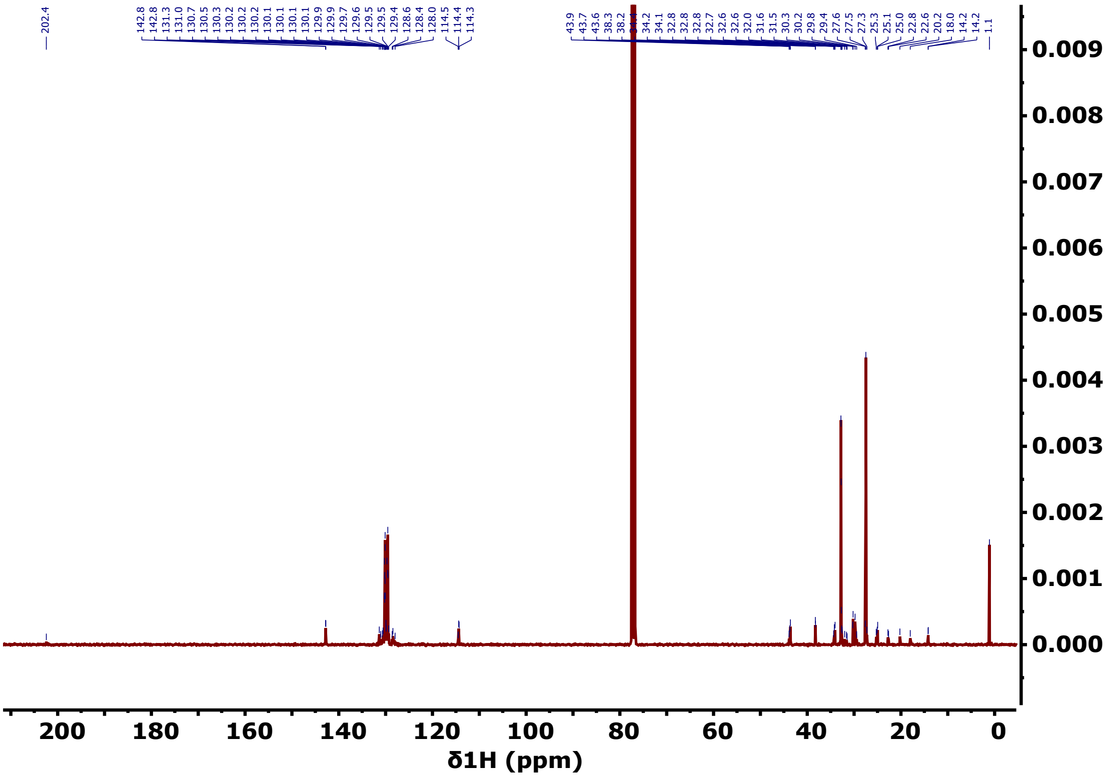

Figure S51: *^{13}C NMR spectrum of the cleaved diol functionalised polybutadiene to aldehydes, measured in chloroform-d at 25 °C.*

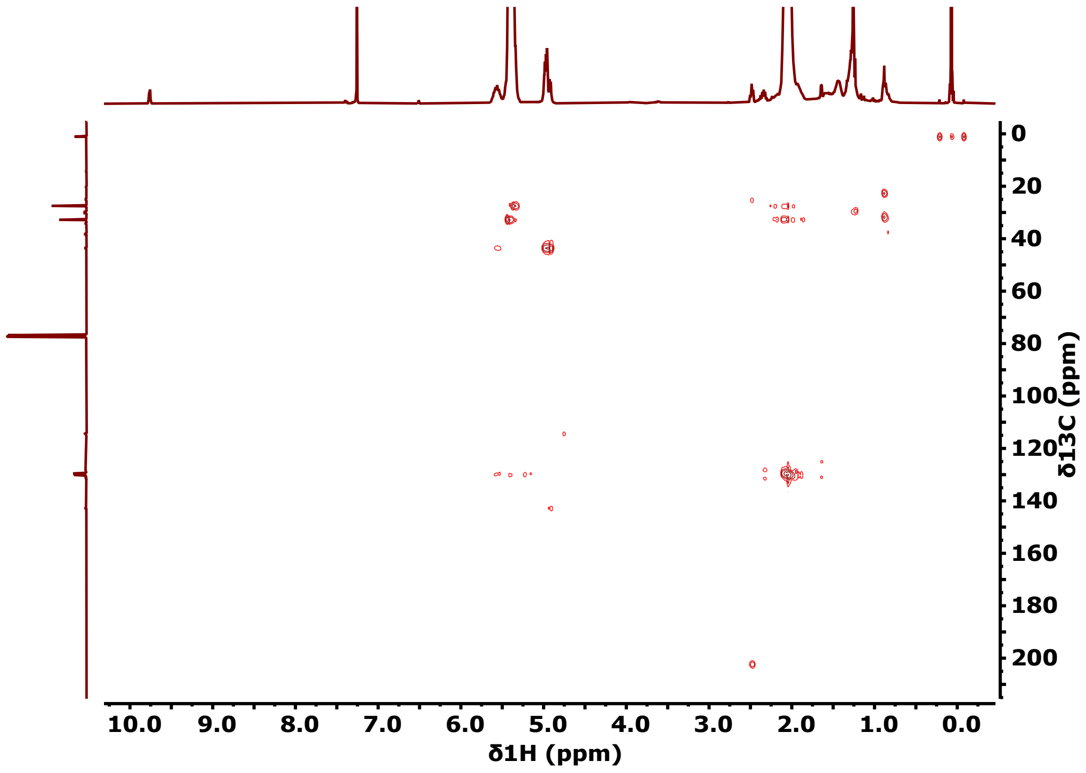

Figure S52: *$^{1}H^{13}C$ HMBC NMR spectrum of the cleaved diol functionalised polybutadiene to aldehydes, measured in chloroform-d at 25 °C.*

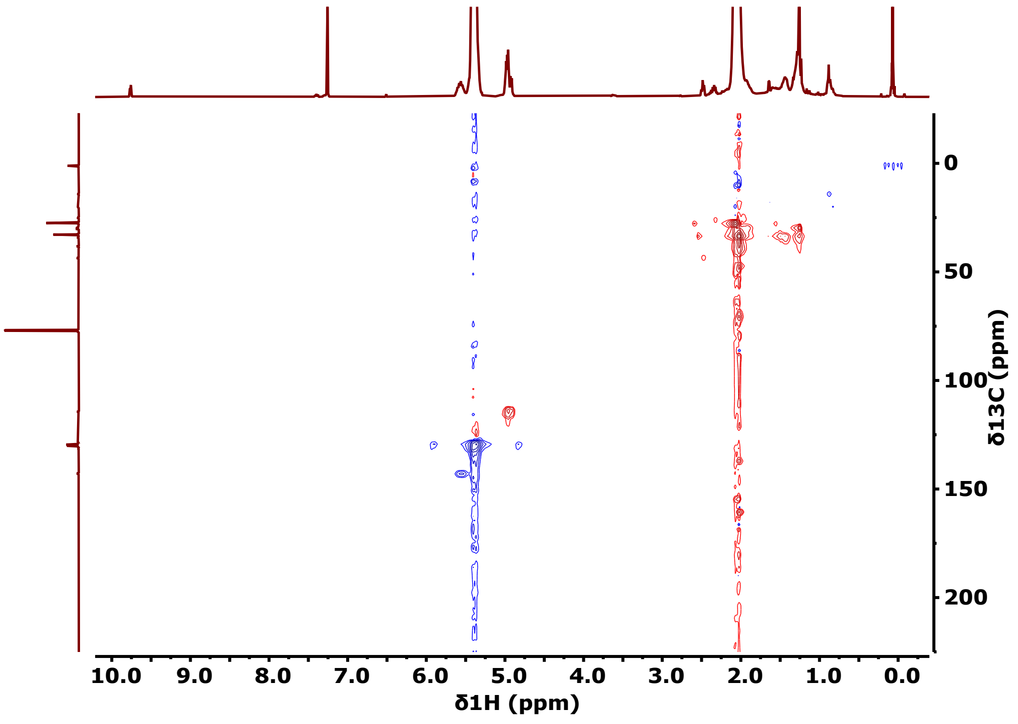

Figure S53: *$^{1}H^{13}C$ HSQC NMR spectrum of the cleaved diol functionalised polybutadiene to aldehydes, measured in chloroform-d at 25 °C.*

NMR Spectra of the reaction mixture of the catalytic oxidation of *trans*-4-octene

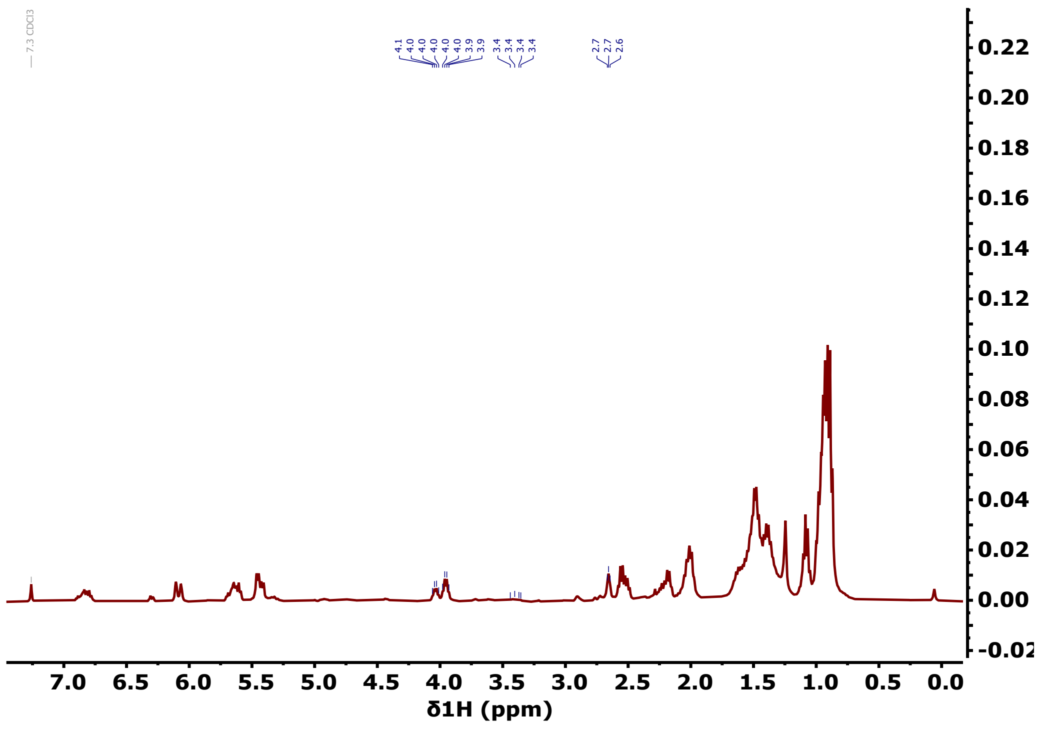

Figure S54: *^{1}H NMR spectrum of the reaction mixture after the catalytic oxidation of trans-4-octene, measured in chloroform-d at 25 °C.*

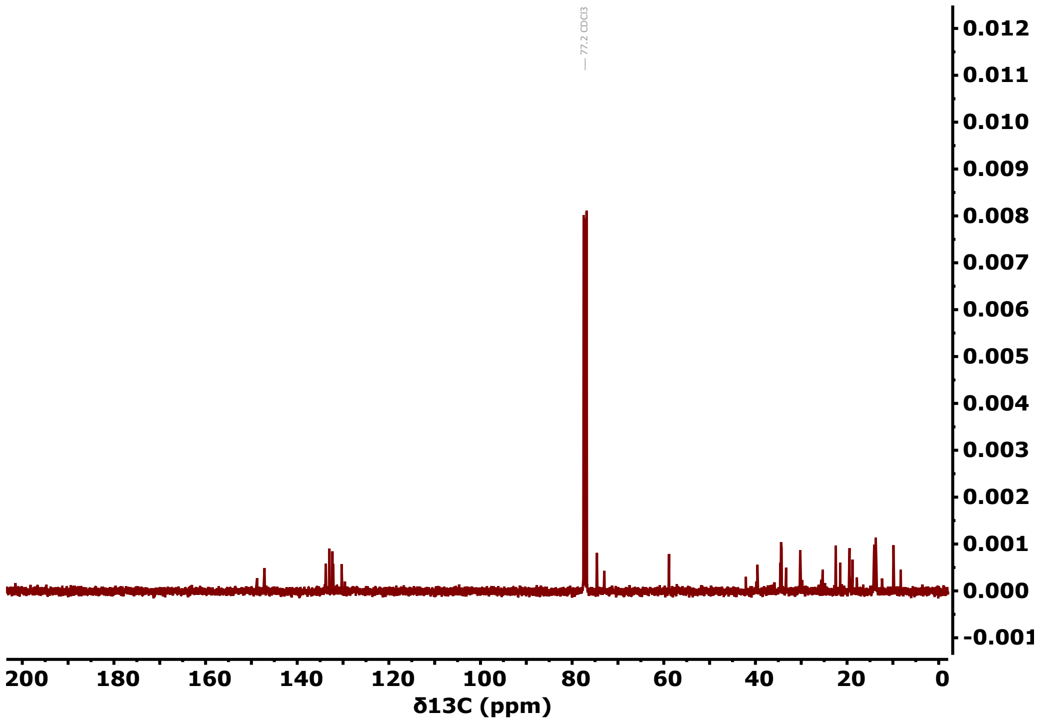

Figure S55: *^{13}C NMR spectrum of the reaction mixture after the catalytic oxidation of trans-4-octene, measured in chloroform-d at 25 °C.*

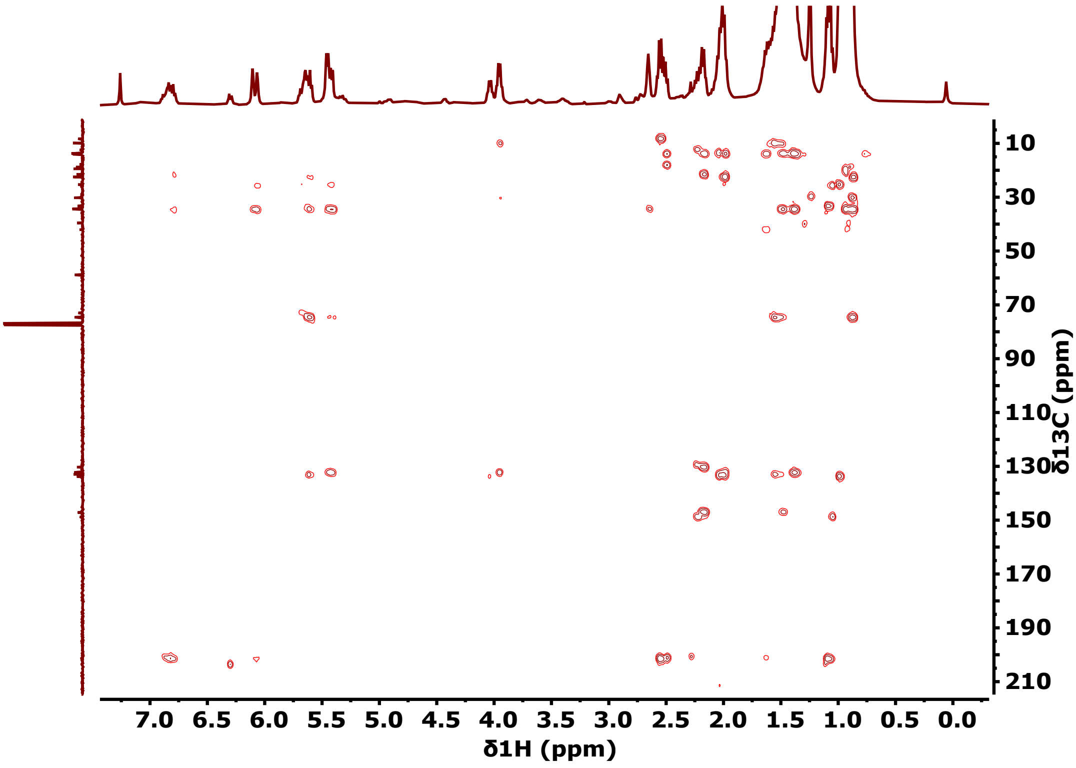

Figure S56: *^{1}H^{13}C HMBC NMR spectrum of the reaction mixture after the catalytic oxidation of trans-4-octene, measured in chloroform-d at 25 °C.*

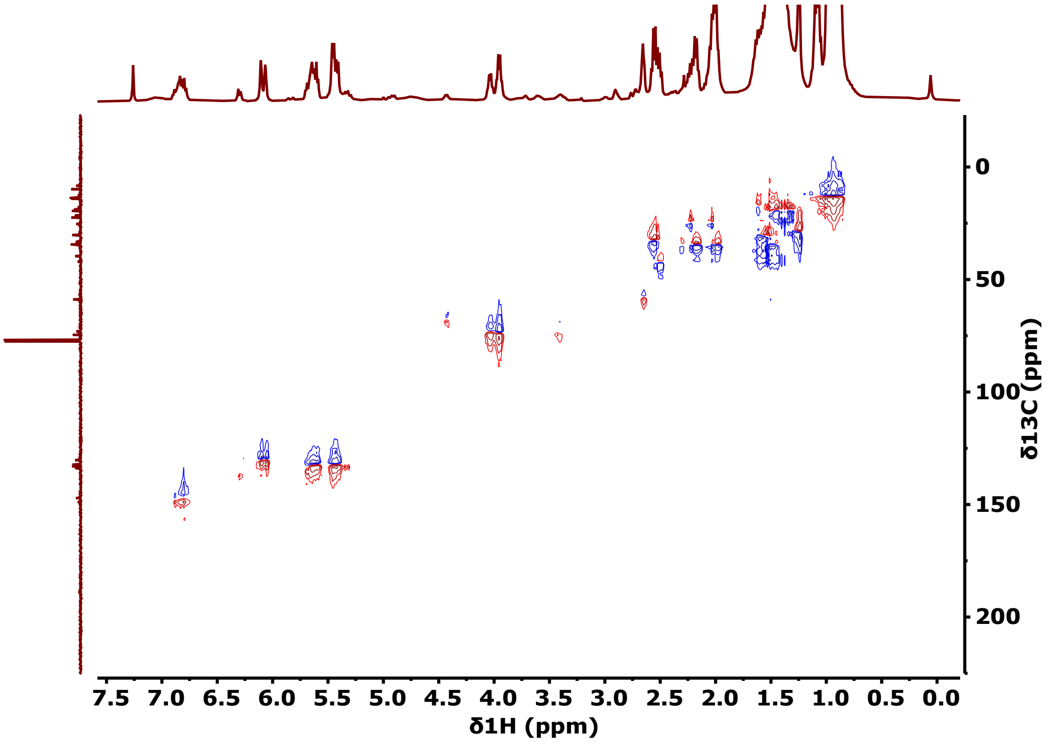

Figure S57: *^{1}H^{13}C HSQC NMR spectrum of the reaction mixture after the catalytic oxidation of trans-4-octene, measured in chloroform-d at 25 °C.*

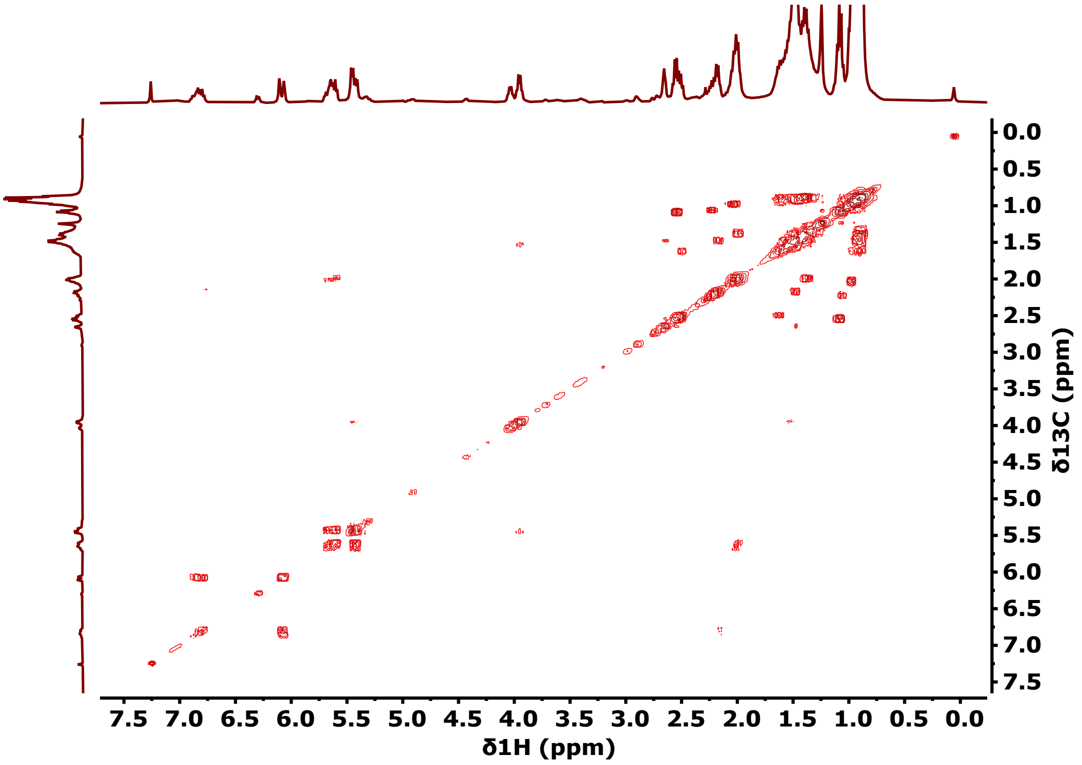

Figure S58: *$^1H^1H$ COSY NMR spectrum of the reaction mixture after the catalytic oxidation of trans-4-octene, measured in chloroform-d at 25 °C.*

3. DSC and TGA Data

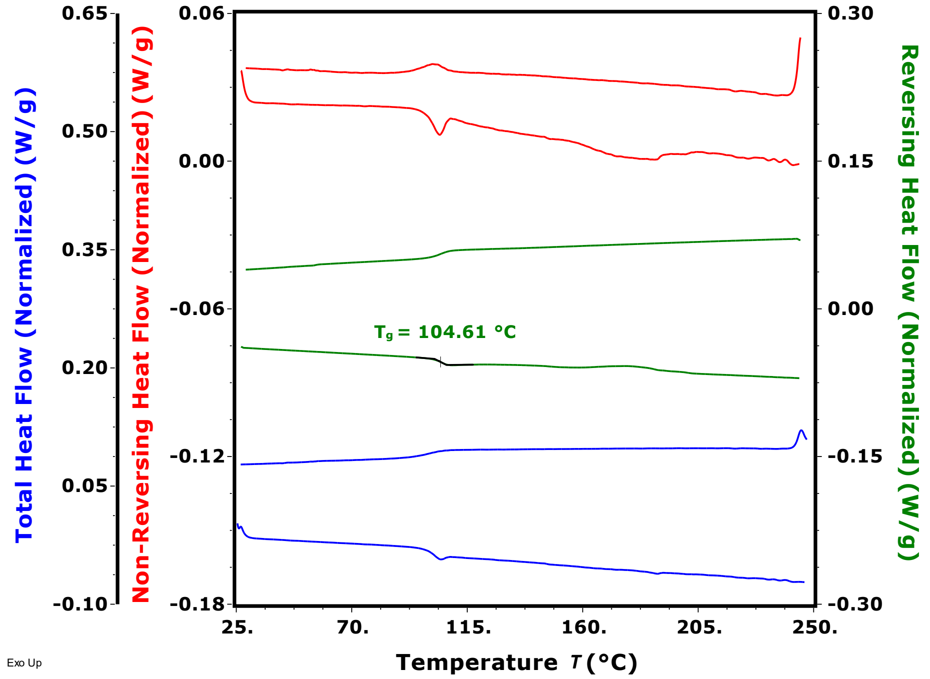

Figure S59: *Differential Scanning Calorimetry (DSC) of the unfunctionalised high molecular weight PS. The Blue trace depicts the normalized total heat flow, the green trace depicts the normalized reversing heat flow, and the red trace depicts the normalized non-reversed heat flow.*

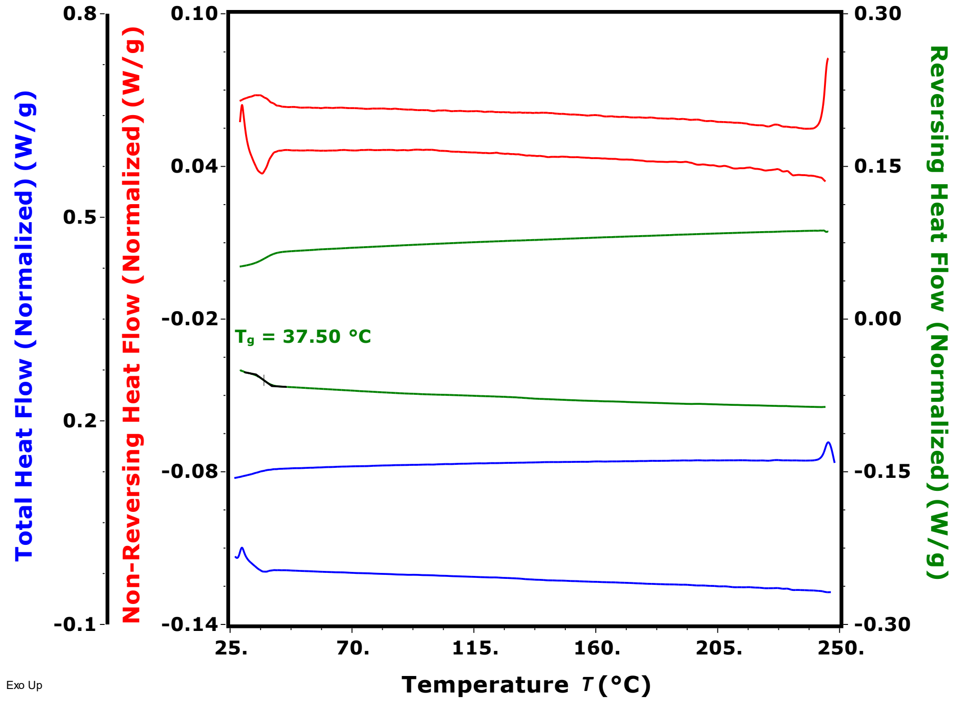

Figure S60: *Differential Scanning Calorimetry (DSC) of the unfunctionalised low molecular weight PS. The Blue trace depicts the normalized total heat flow, the green trace depicts the normalized reversing heat flow, and the red trace depicts the normalized non-reversed heat flow.*

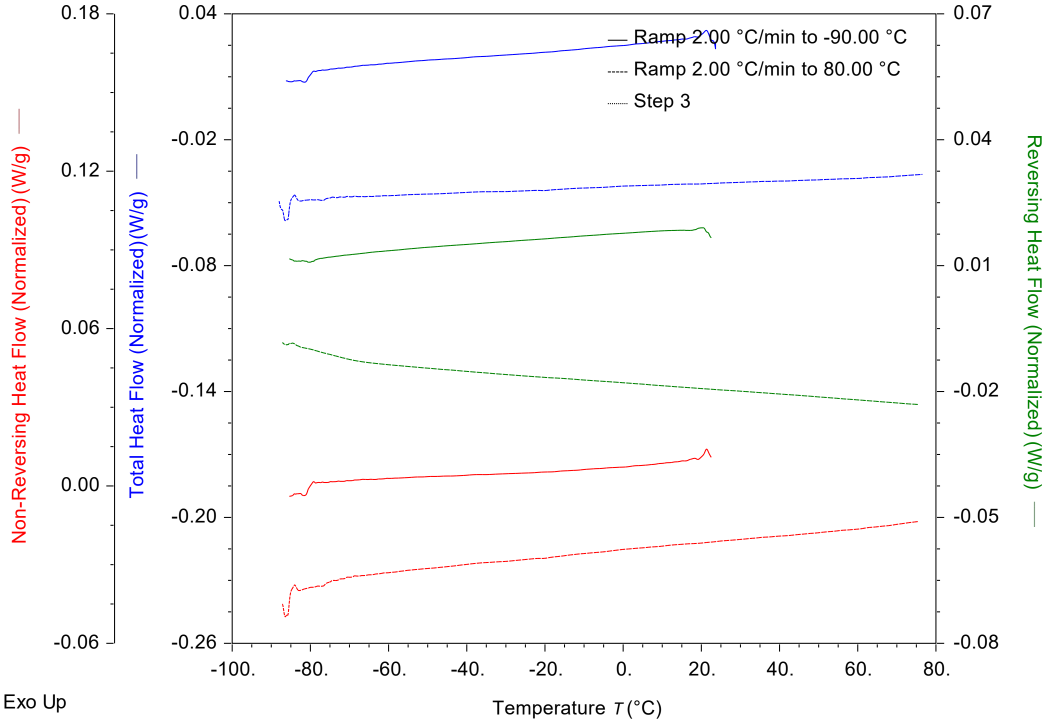

Figure S61: *Differential Scanning Calorimetry (DSC) of the unfunctionalised PBD8020. The Blue trace depicts the normalized total heat flow, the green trace depicts the normalized reversing heat flow, and the red trace depicts the normalized non-reversed heat flow.*

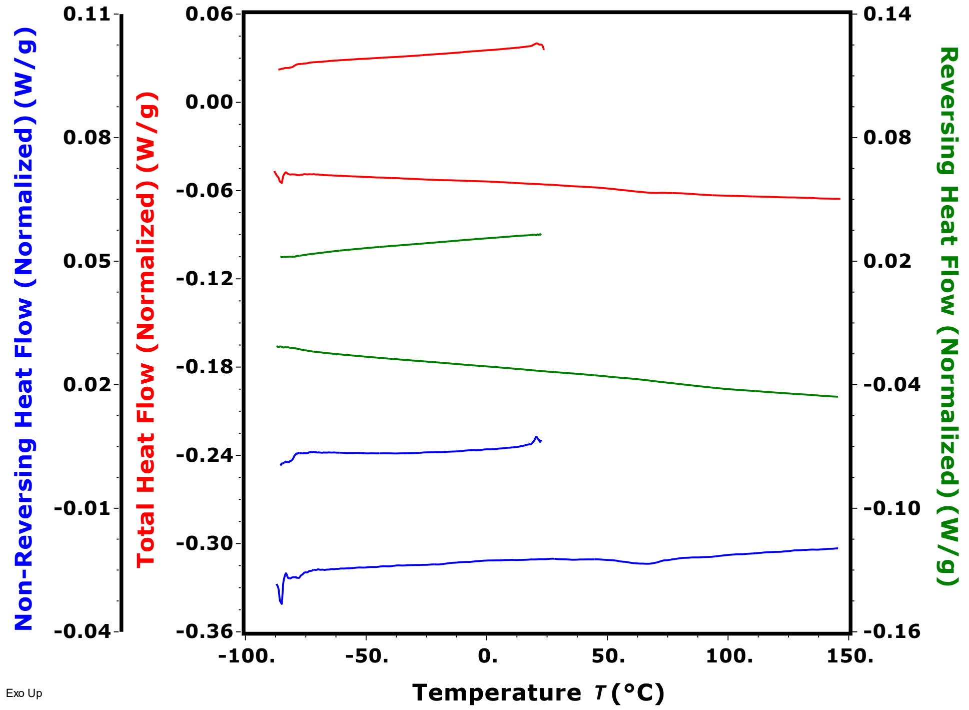

Figure S62: *Differential Scanning Calorimetry (DSC) of the unfunctionalised styrene-butadiene-styrene (SBS). The Blue trace depicts the normalized total heat flow, the green trace depicts the normalized reversing heat flow, and the red trace depicts the normalized non-reversed heat flow.*

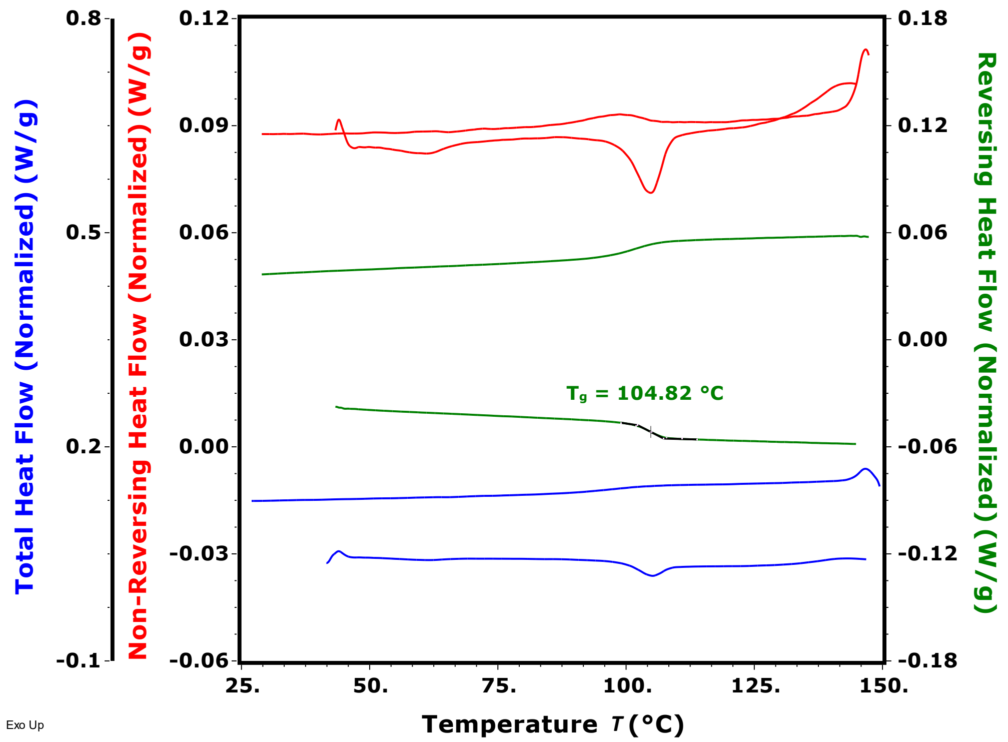

Figure S63: *Differential Scanning Calorimetry (DSC) of the oxyfunctionalised high molecular weight PS. The Blue trace depicts the normalized total heat flow, the green trace depicts the normalized reversing heat flow, and the red trace depicts the normalized non-reversed heat flow.*

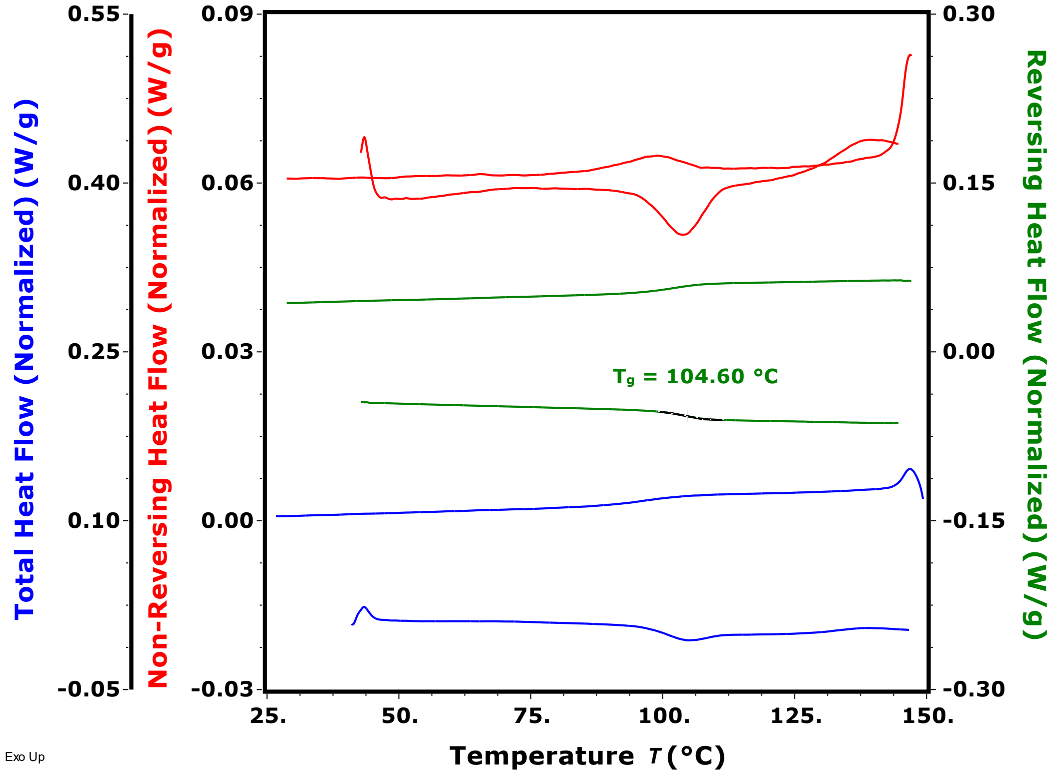

Figure S64: *Differential Scanning Calorimetry (DSC) of the oxyfunctionalised high molecular weight PS. The Blue trace depicts the normalized total heat flow, the green trace depicts the normalized reversing heat flow, and the red trace depicts the normalized non-reversed heat flow.*

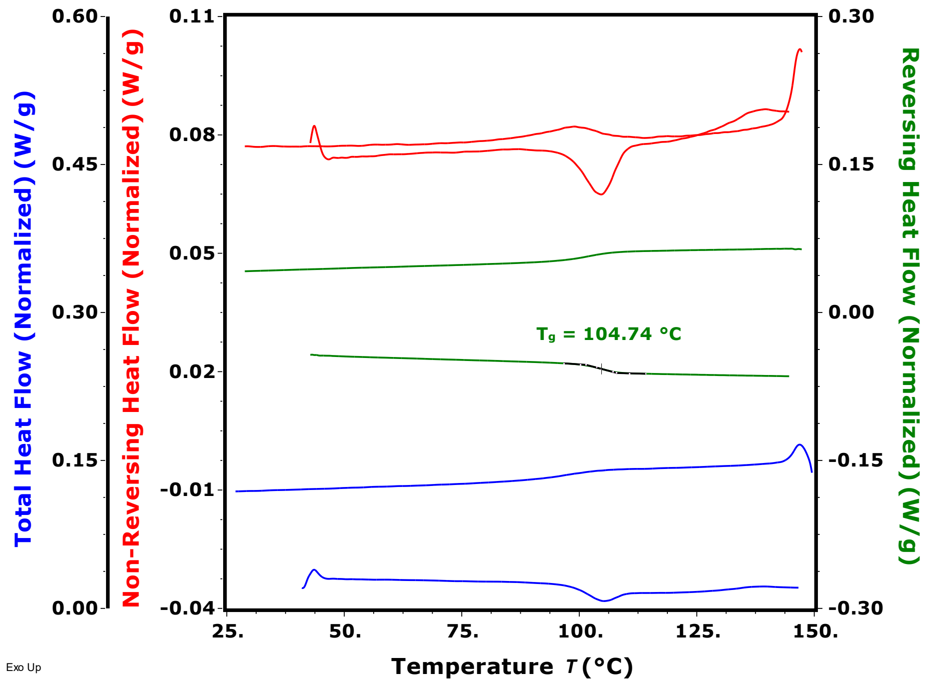

Figure S65: *Differential Scanning Calorimetry (DSC) of the oxyfunctionalised high molecular weight PS. The Blue trace depicts the normalized total heat flow, the green trace depicts the normalized reversing heat flow, and the red trace depicts the normalized non-reversed heat flow.*

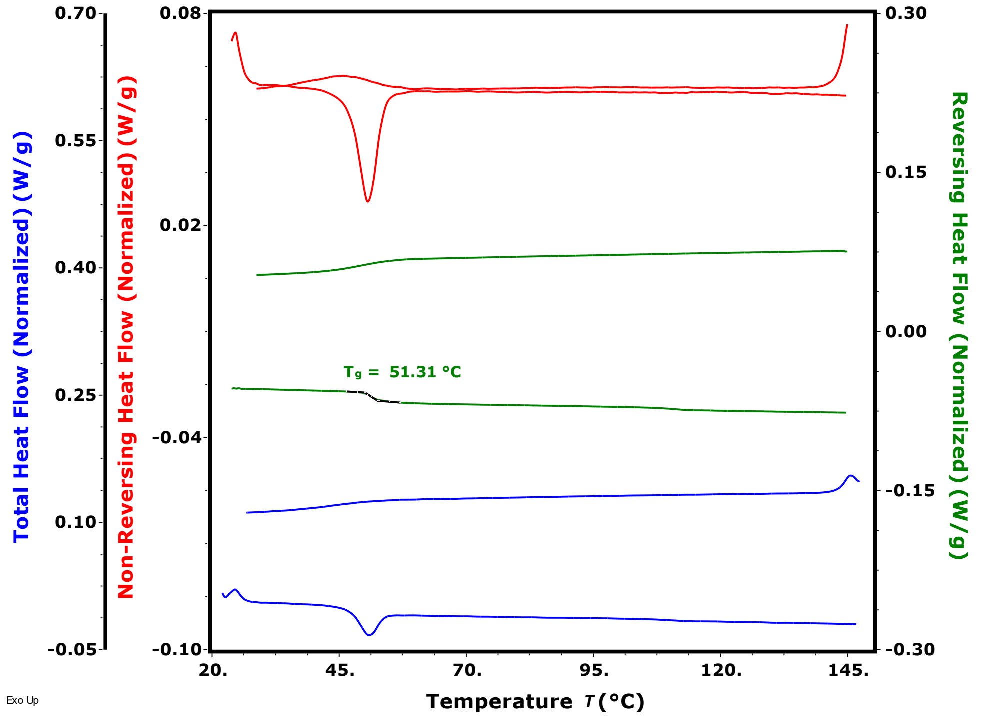

Figure S66: *Differential Scanning Calorimetry (DSC) of the oxyfunctionalised low molecular weight PS. The Blue trace depicts the normalized total heat flow, the green trace depicts the normalized reversing heat flow, and the red trace depicts the normalized non-reversed heat flow.*

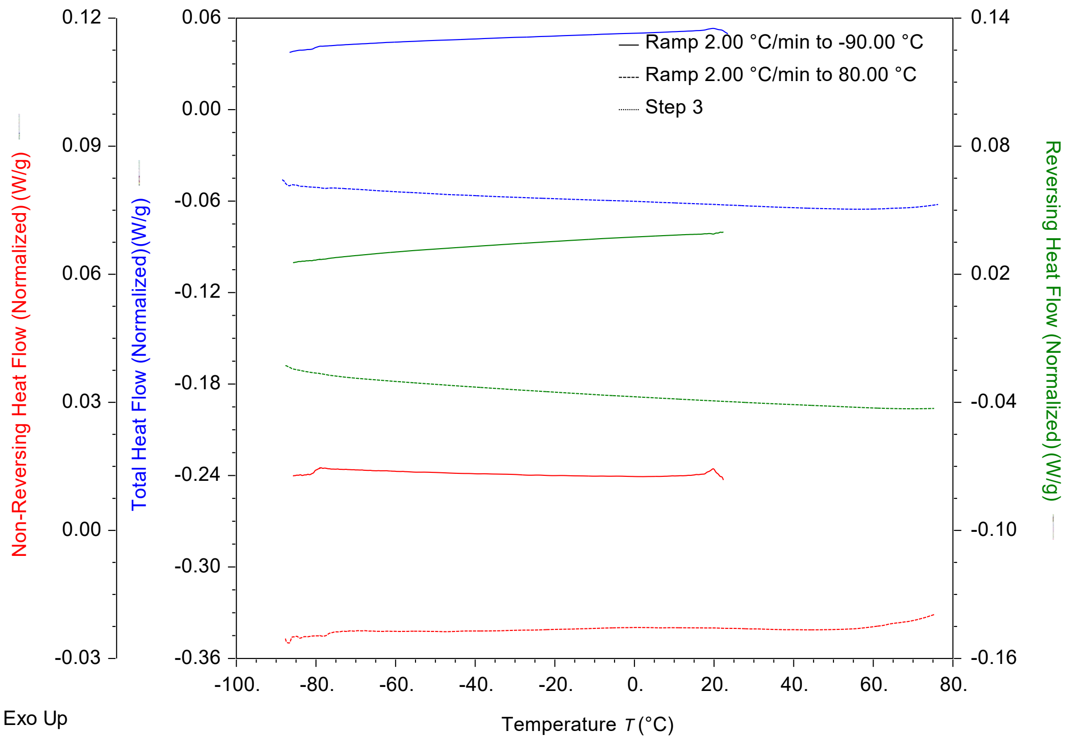

***Figure S67:** Differential Scanning Calorimetry (DSC) of the oxyfunctionalised PBD8020. The Blue trace depicts the normalized total heat flow, the green trace depicts the normalized reversing heat flow, and the red trace depicts the normalized non-reversed heat flow.*

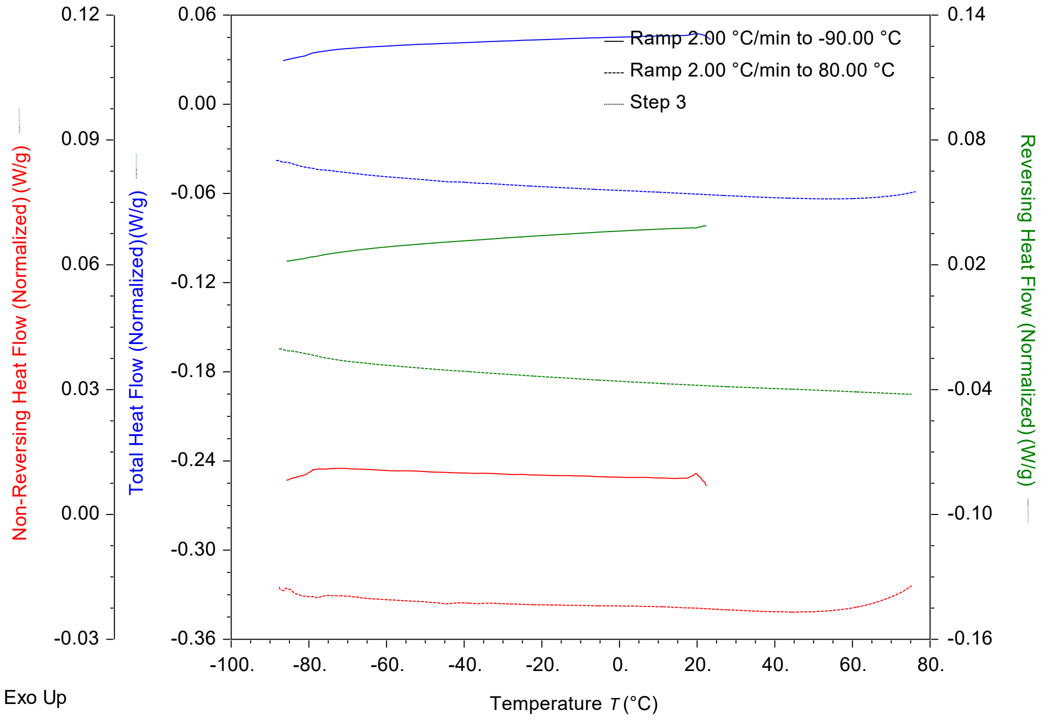

***Figure S68:** Differential Scanning Calorimetry (DSC) of the oxyfunctionalised PBD8020. The Blue trace depicts the normalized total heat flow, the green trace depicts the normalized reversing heat flow, and the red trace depicts the normalized non-reversed heat flow.*

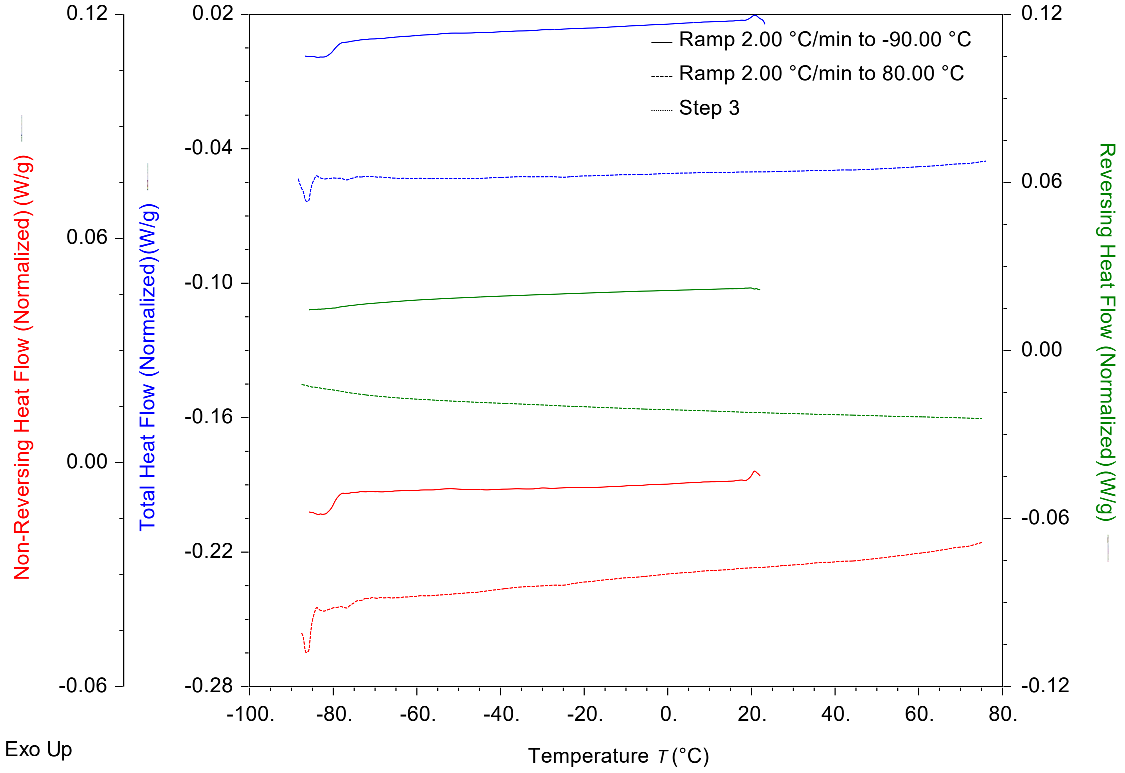

Figure S69: *Differential Scanning Calorimetry (DSC) of the oxyfunctionalised PBD8020. The Blue trace depicts the normalized total heat flow, the green trace depicts the normalized reversing heat flow, and the red trace depicts the normalized non-reversed heat flow.*

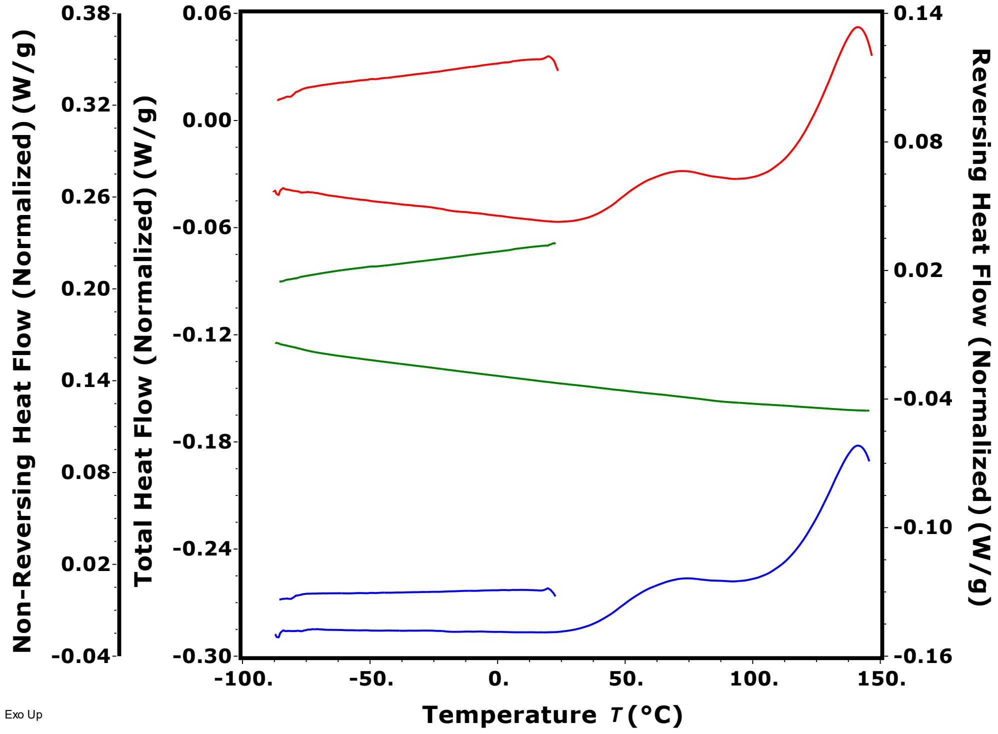

Figure S70: *Differential Scanning Calorimetry (DSC) of the oxyfunctionalised styrene-butadiene-styrene (SBS). The Blue trace depicts the normalized total heat flow, the green trace depicts the normalized reversing heat flow, and the red trace depicts the normalized non-reversed heat flow.*

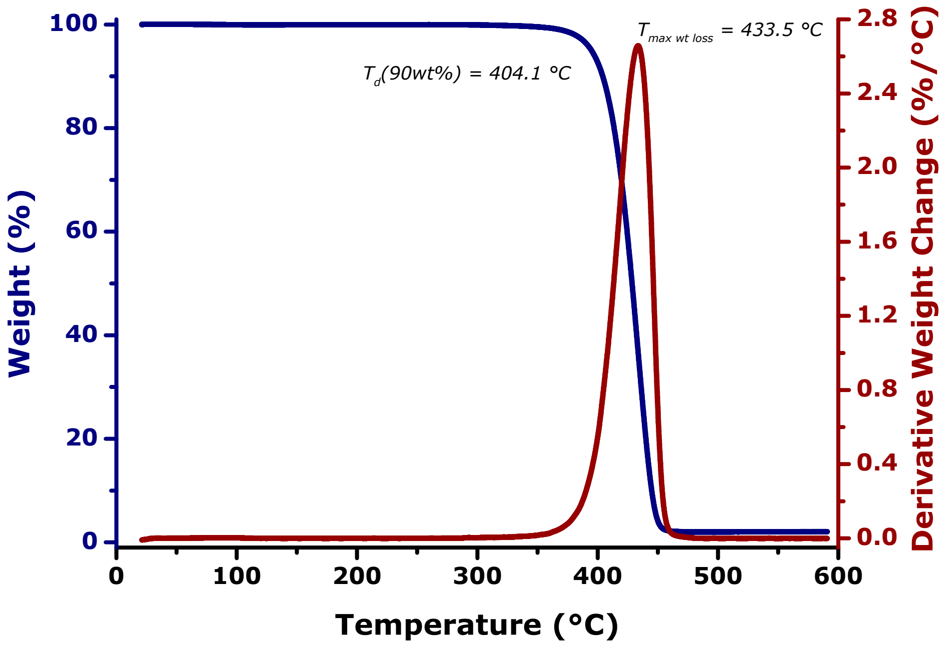

Figure S71: *Thermogravimetric Analysis (TGA) of the unfunctionalised high molecular weight PS. The Blue trace depicts the normalized total heat flow, the green trace depicts the normalized reversing heat flow, and the red trace depicts the normalized non-reversed heat flow.*

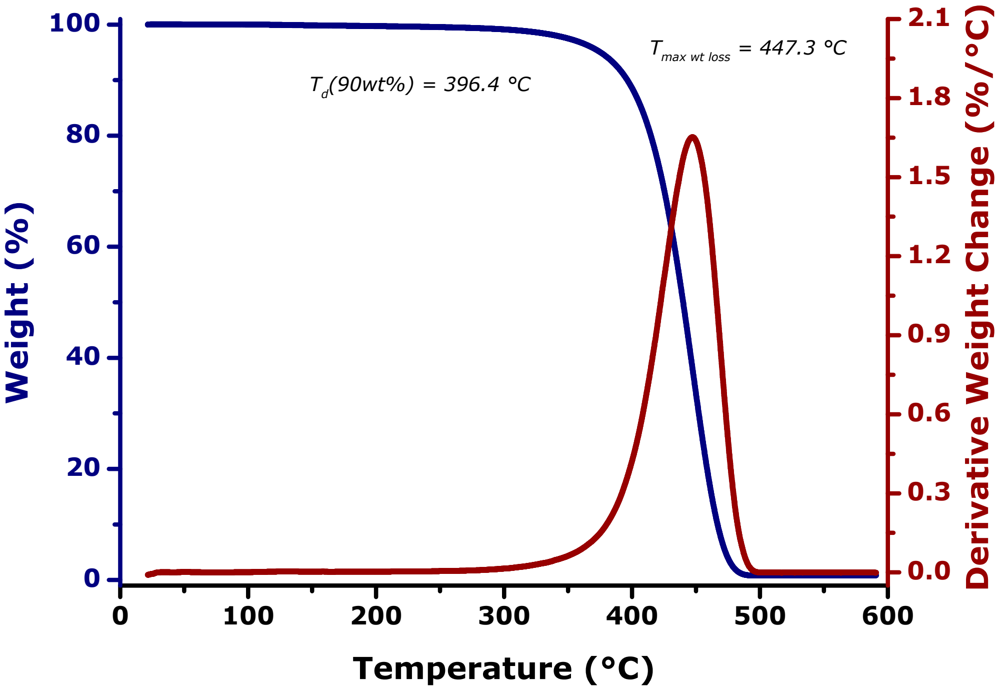

Figure S72: *Thermogravimetric Analysis (TGA) of the unfunctionalised low molecular weight PS. The Blue trace depicts the normalized total heat flow, the green trace depicts the normalized reversing heat flow, and the red trace depicts the normalized non-reversed heat flow.*

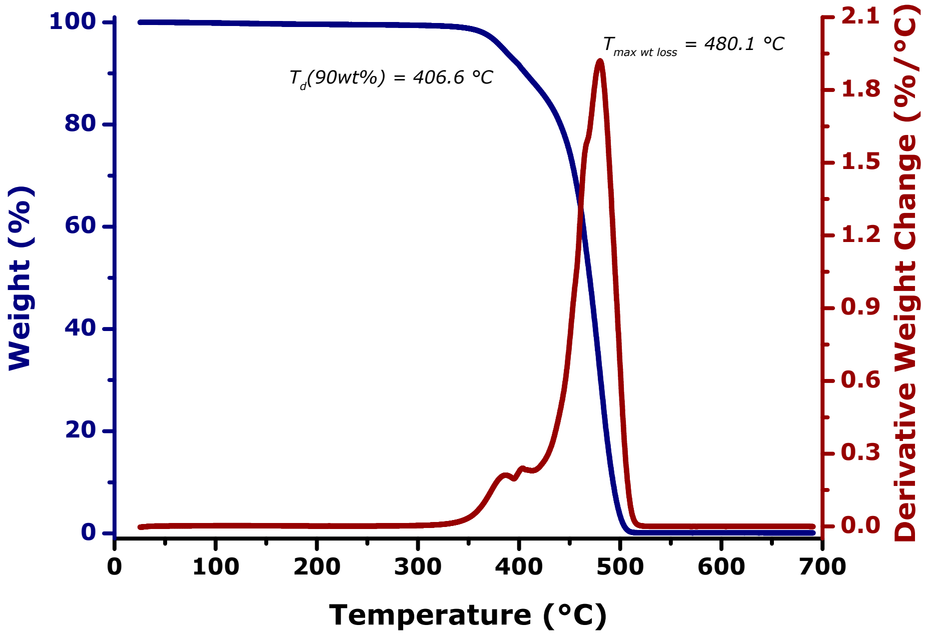

Figure S73: *Thermogravimetric Analysis (TGA) of the unfunctionalised PBD8020. The Blue trace depicts the normalized total heat flow, the green trace depicts the normalized reversing heat flow, and the red trace depicts the normalized non-reversed heat flow.*

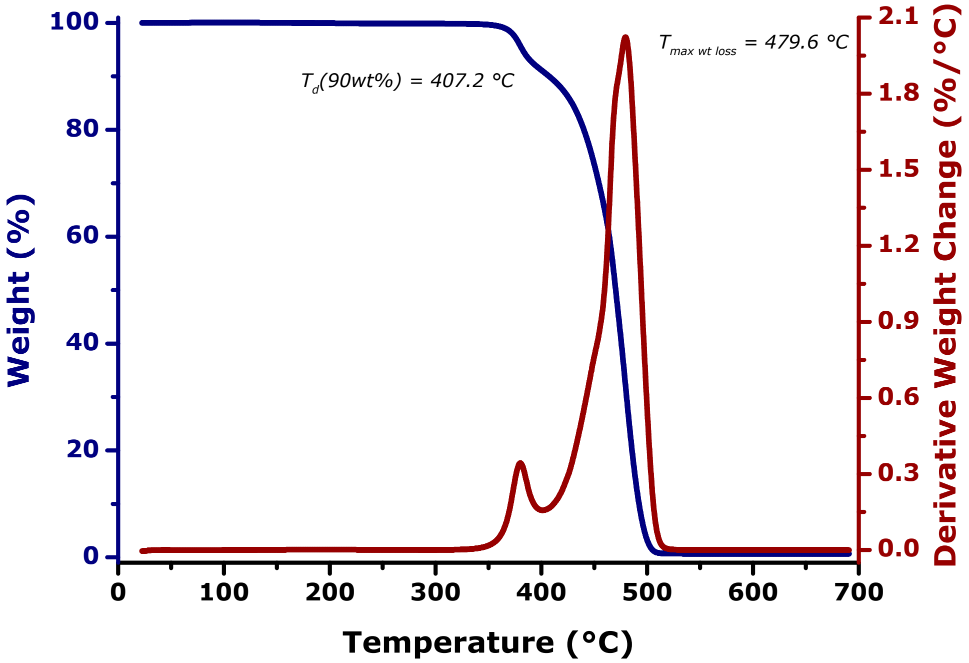

Figure S74: *Thermogravimetric Analysis (TGA) of the unfunctionalised styrene-butadiene-styrene (SBS). The Blue trace depicts the normalized total heat flow, the green trace depicts the normalized reversing heat flow, and the red trace depicts the normalized non-reversed heat flow.*

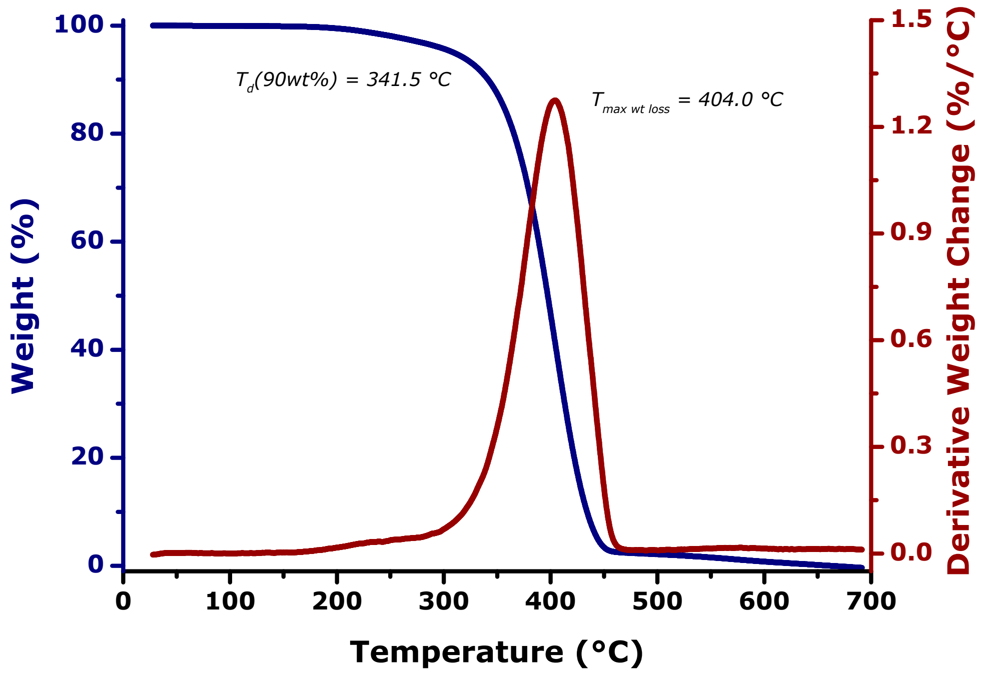

Figure S75: *Thermogravimetric Analysis (TGA) of oxyfunctionalised high M_w PS. The Blue trace depicts the normalized total heat flow, the green trace depicts the normalized reversing heat flow, and the red trace depicts the normalized non-reversed heat flow.*

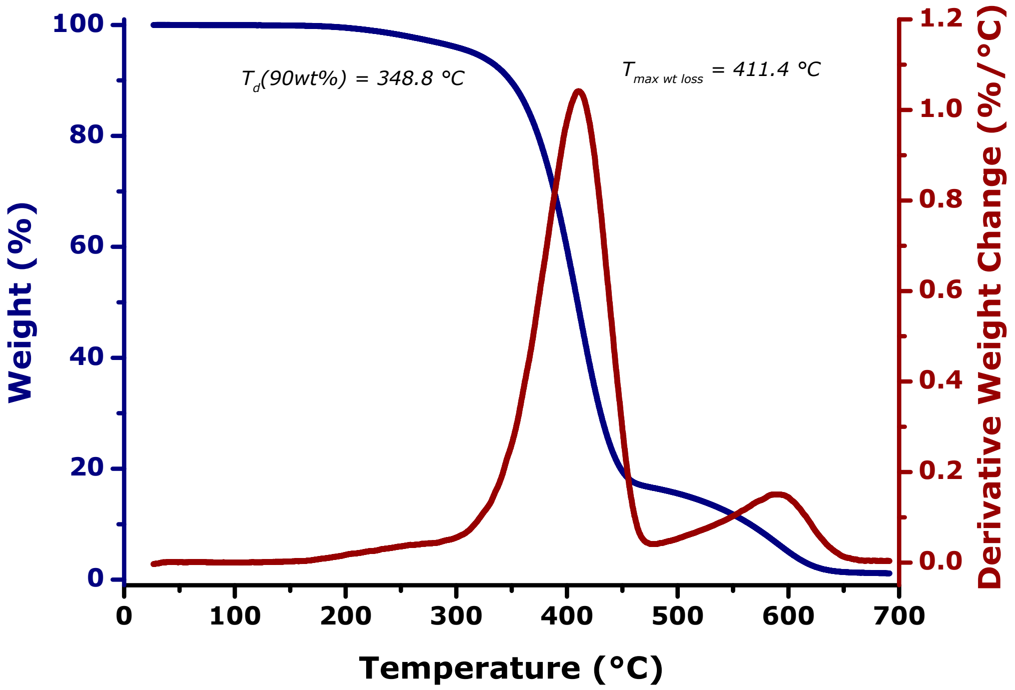

Figure S76: *Thermogravimetric Analysis (TGA) of oxyfunctionalised high M_w PS. The Blue trace depicts the normalized total heat flow, the green trace depicts the normalized reversing heat flow, and the red trace depicts the normalized non-reversed heat flow.*

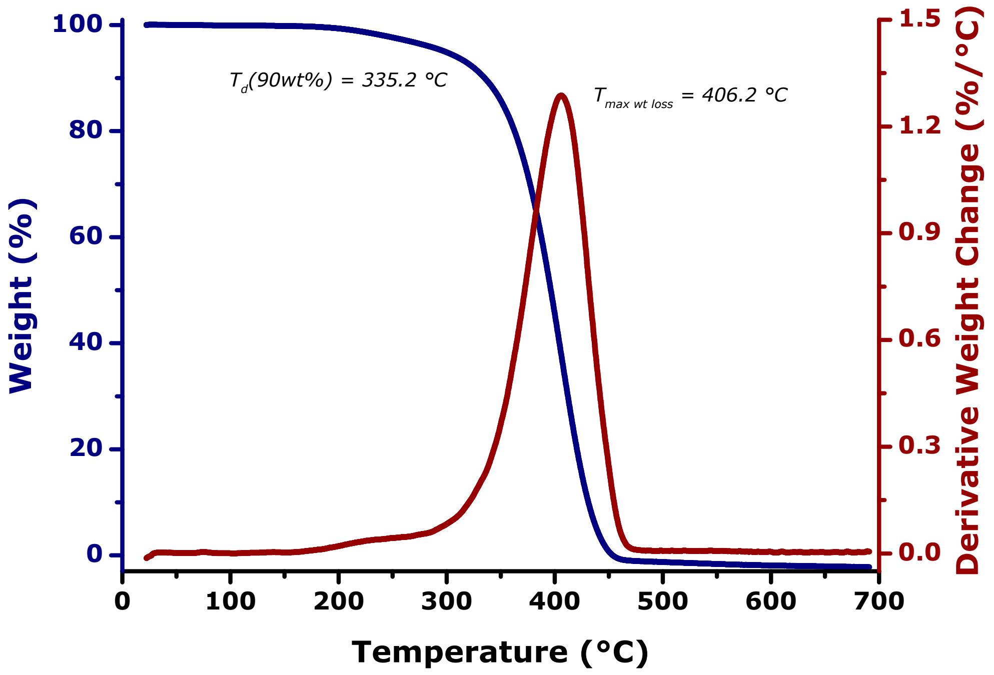

Figure S77: *Thermogravimetric Analysis (TGA) of oxyfunctionalised high M_w PS (entry 4). The Blue trace depicts the normalized total heat flow, the green trace depicts the normalized reversing heat flow, and the red trace depicts the normalized non-reversed heat flow.*

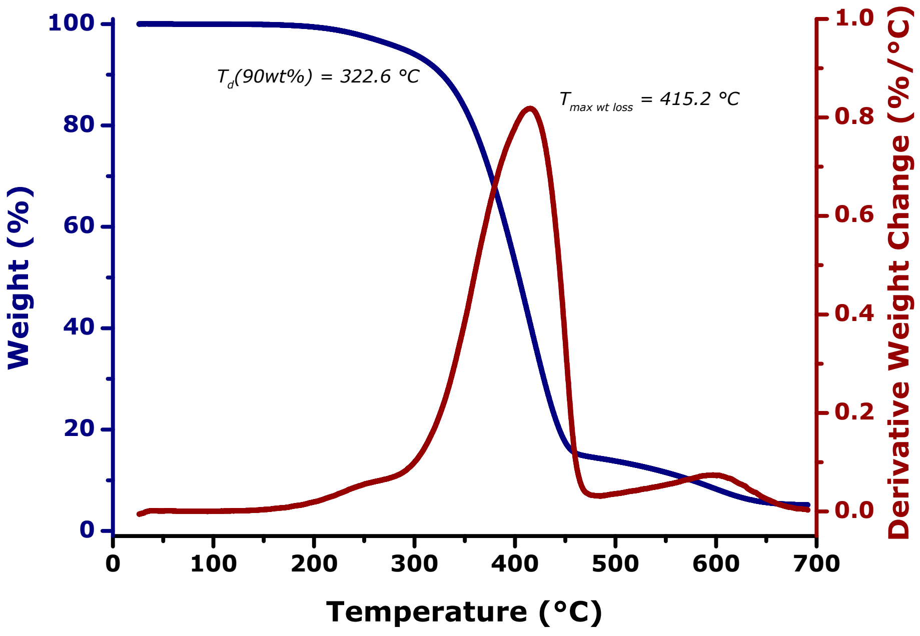

Figure S78: *Thermogravimetric Analysis (TGA) of oxyfunctionalised low M_w PS. The Blue trace depicts the normalized total heat flow, the green trace depicts the normalized reversing heat flow, and the red trace depicts the normalized non-reversed heat flow.*

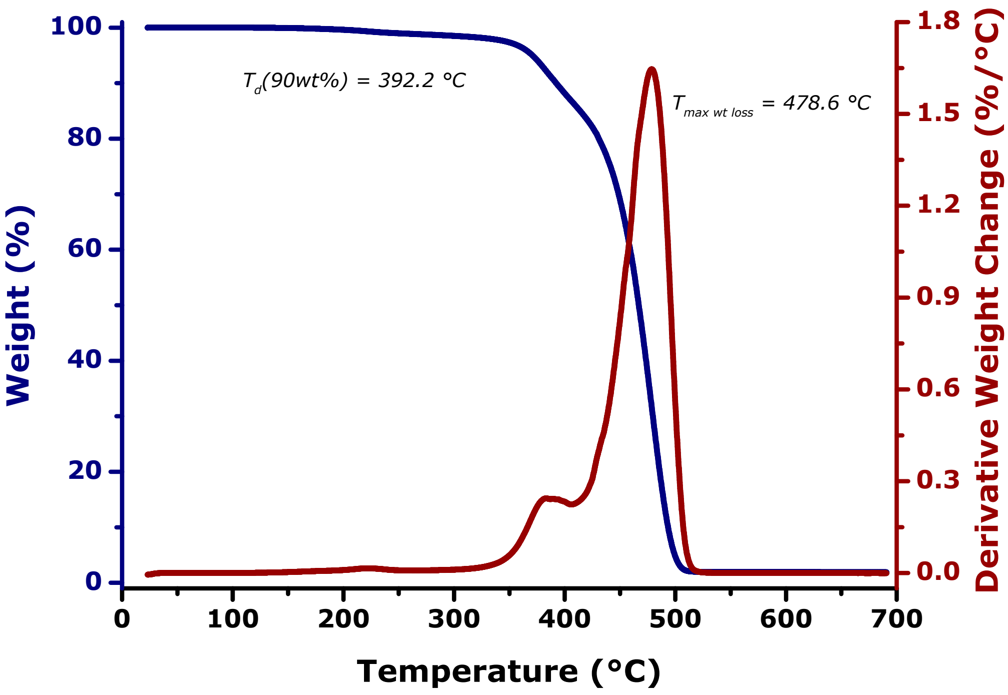

Figure S79: *Thermogravimetric Analysis (TGA) of oxyfunctionalised PBD820. The Blue trace depicts the normalized total heat flow, the green trace depicts the normalized reversing heat flow, and the red trace depicts the normalized non-reversed heat flow.*

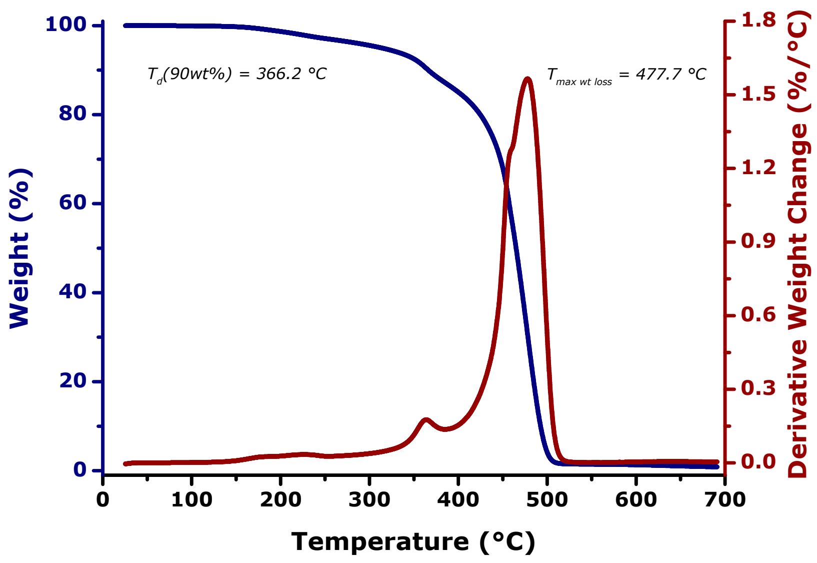

Figure S80: *Thermogravimetric Analysis (TGA) of oxyfunctionalised PBD820. The Blue trace depicts the normalized total heat flow, the green trace depicts the normalized reversing heat flow, and the red trace depicts the normalized non-reversed heat flow.*

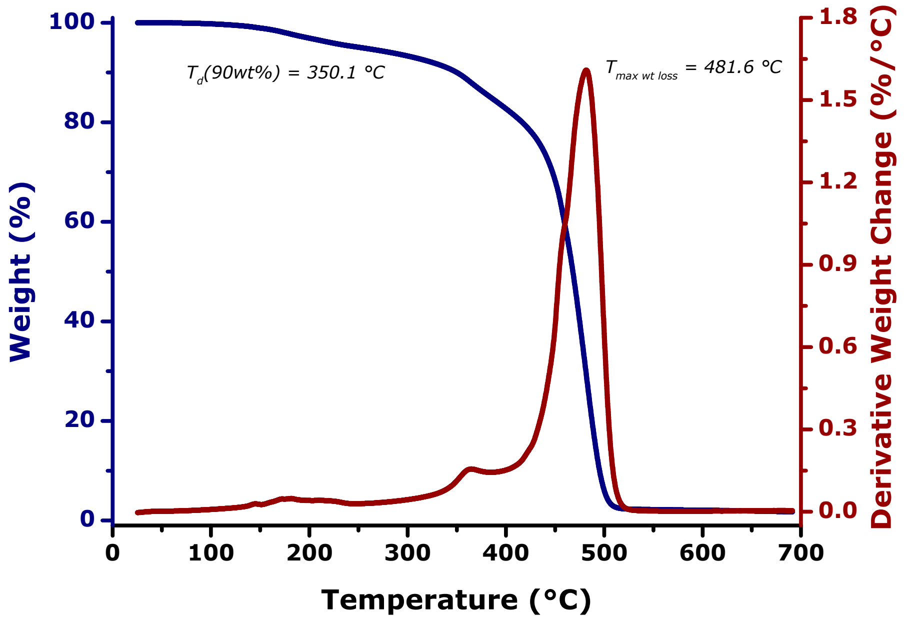

Figure S81: *Thermogravimetric Analysis (TGA) of oxyfunctionalised PBD820 (entry 10). The Blue trace depicts the normalized total heat flow, the green trace depicts the normalized reversing heat flow, and the red trace depicts the normalized non-reversed heat flow.*

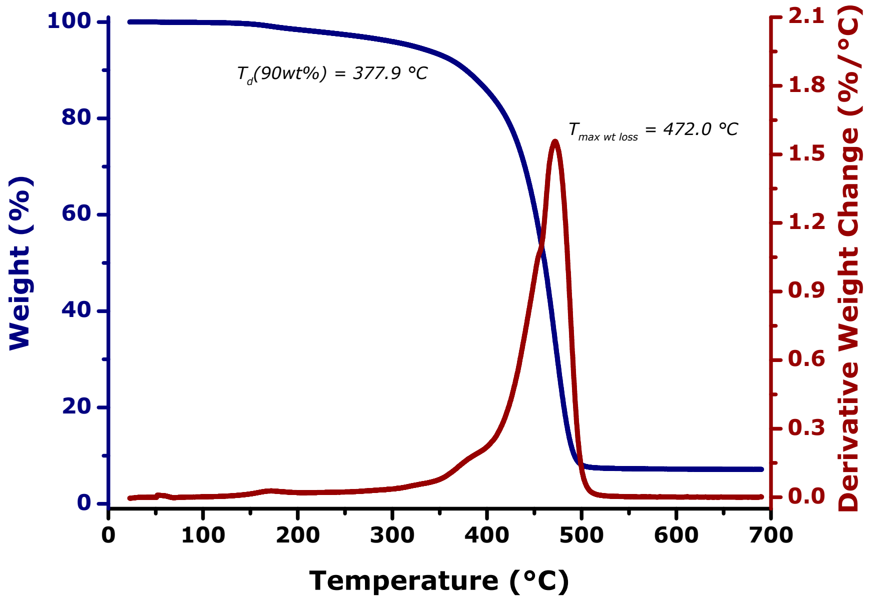

Figure S82: *Thermogravimetric Analysis (TGA) of oxyfunctionalised styrene-butadiene-styrene (SBS) (entry 12). The Blue trace depicts the normalized total heat flow, the green trace depicts the normalized reversing heat flow, and the red trace depicts the normalized non-reversed heat flow.*

4. GPC Data

For the high and mid molecular weight OPS entries and PS starting materials, GPC was measured in DMF using a refractive index detector. All samples were measured in duplo. The data for both starting material and oxidised polymer material is determined using the same calibration, shown below unless shown different.

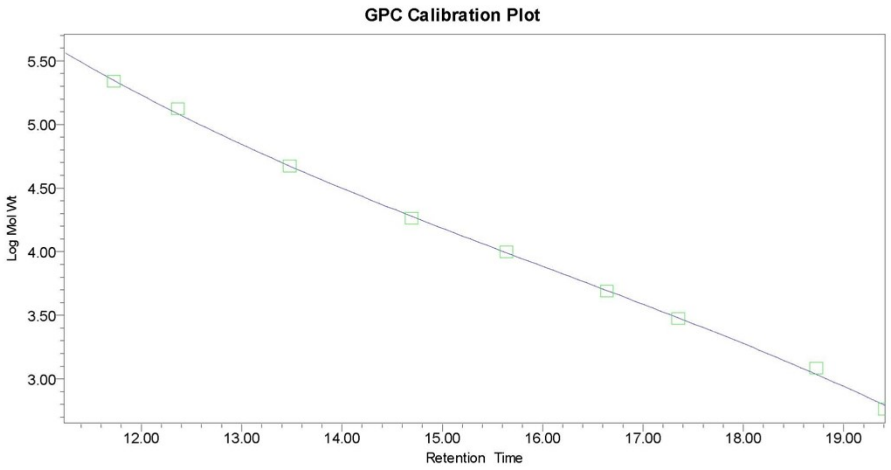

GPC Calibration Table

	Mol Wt (Daltons)	RT (min)	Calculated Weight (Daltons)	% Residual
1	364000	11.231	372441	-2.266
2	133300	12.364	122202	9.082
3	18340	14.692	19126	-4.112
4	4900	16.640	4985	-1.696
5	1220	18.726	1093	11.657
6	217900	11.727	223745	-2.612
7	47190	13.479	47500	-0.653
8	9960	15.638	9881	0.804
9	2980	17.350	3043	-2.064
10	580	19.411	622	-6.695

Figure S83: *GPC calibration Plot for the measurements in DMF.*

PS High M_w

SAMPLE INFORMATION

Sample Name:	PS_High_Mw	Acquired By:	Waters
Sample Type:	Broad Unknown	Sample Set Name:	20250422_MO_NK_OPS
Vial:	5	Acq. Method Set:	AI 100A@1 RI UV280_65C
Injection #:	2	Processing Method:	20250423 PS RI
Injection Volume:	50.00 ul	Channel Name:	W2414 RI
Run Time:	30.0 Minutes	Proc. Chnl. Descr.:	W2414 RI
Date Acquired:	22-Apr-25 20:15:35 CEST		
Date Processed:	23-Apr-25 11:49:09 CEST		

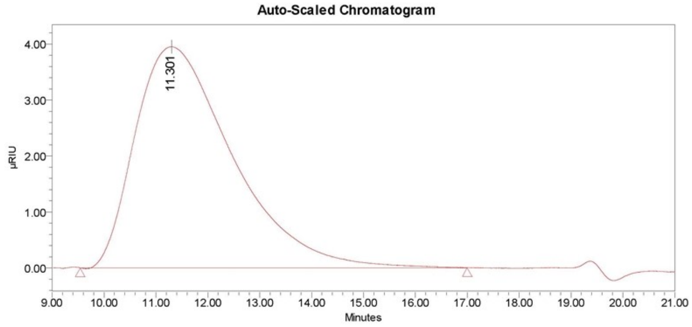

GPC Results

	SampleName	Mw	MP	Mn	Polydispersity	Area (µV*sec)	% Area
1	PS_High_Mw					514861	100.00

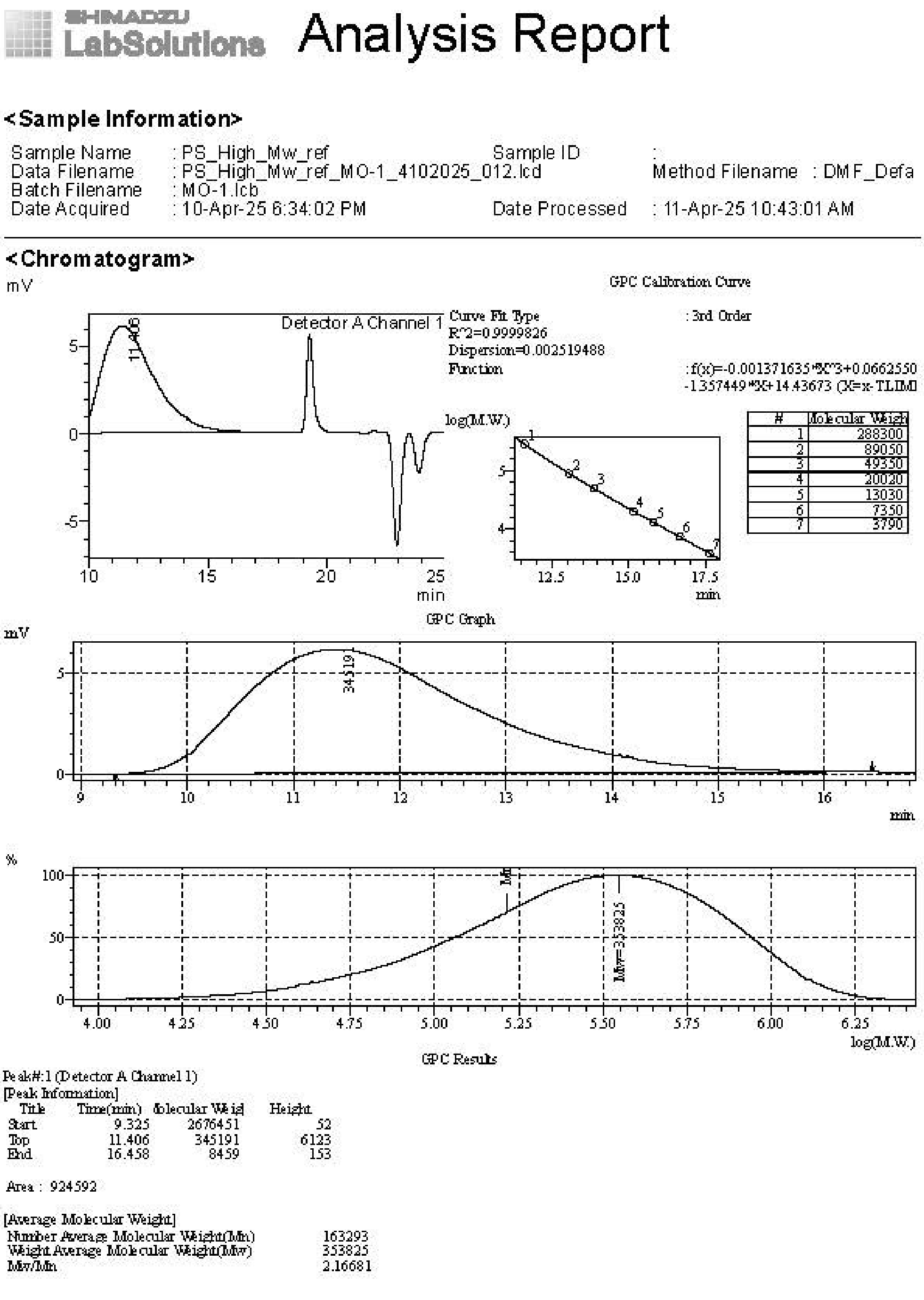

Figure S84: *GPC traces of the unfunctionalised High M_w PS on two different GPCs running both on DMF.*

OxyPS High M_w

SAMPLE INFORMATION

Sample Name:	NK014	Acquired By:	Waters
Sample Type:	Broad Unknown	Sample Set Name:	20250422_MO_NK_OPS
Vial:	7	Acq. Method Set:	AI 100A@1 RI UV280_65C
Injection #:	2	Processing Method:	20250423 PS RI
Injection Volume:	50.00 ul	Channel Name:	W2414 RI
Run Time:	30.0 Minutes	Proc. Chnl. Descr.:	W2414 RI
Date Acquired:	22-Apr-25 22:19:38 CEST		
Date Processed:	23-Apr-25 11:47:57 CEST		

Auto-Scaled Chromatogram

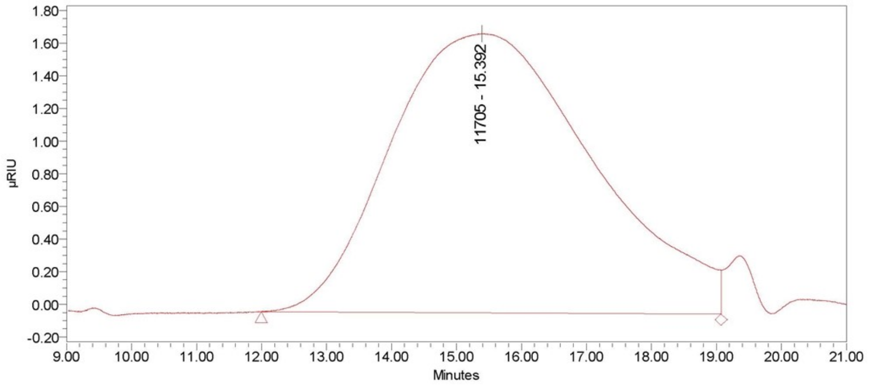

GPC Results

	SampleName	Mw	MP	Mn	Polydispersity	Area (µV*sec)	% Area
1	NK014	14653	11705	5906	2.480943	369472	100.00

Figure S85: *GPC trace of the oxyfunctionalised High M_w PS.*

OxyPS High M_w

SAMPLE INFORMATION

Sample Name:	NK028	Acquired By:	Waters
Sample Type:	Broad Unknown	Sample Set Name:	20250422_MO_NK_OPS
Vial:	9	Acq. Method Set:	AI 100A@1 RI UV280_65C
Injection #:	1	Processing Method:	20250423 PS RI
Injection Volume:	50.00 ul	Channel Name:	W2414 RI
Run Time:	30.0 Minutes	Proc. Chnl. Descr.:	W2414 RI
Date Acquired:	22-Apr-25 23:52:35 CEST		
Date Processed:	23-Apr-25 11:47:05 CEST		

Auto-Scaled Chromatogram

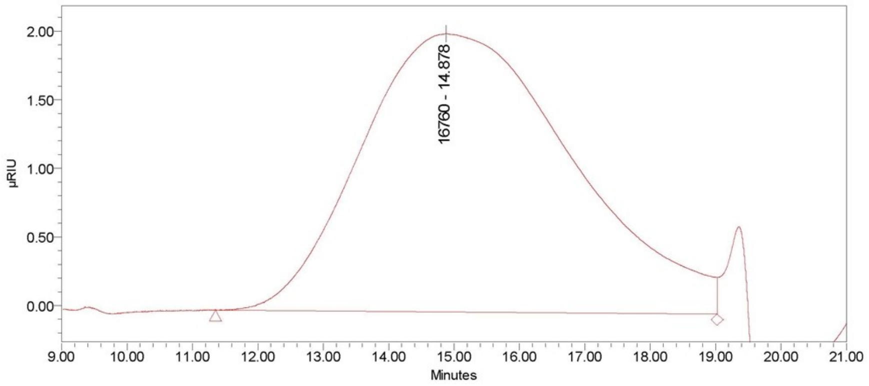

GPC Results

	SampleName	Mw	MP	Mn	Polydispersity	Area (µV*sec)	% Area
1	NK028	19543	16760	7093	2.755378	455251	100.00

Figure S86: *GPC trace of the oxyfunctionalised High M_w PS.*

OxyPS High M_w

SAMPLE INFORMATION			
Sample Name:	NK029	Acquired By:	Waters
Sample Type:	Broad Unknown	Sample Set Name:	20250422_MO_NK_OPS
Vial:	10	Acq. Method Set:	AI 100A@1 RI UV280_65C
Injection #:	1	Processing Method:	20250423 PS RI
Injection Volume:	50.00 ul	Channel Name:	W2414 RI
Run Time:	30.0 Minutes	Proc. Chnl. Descr.:	W2414 RI
Date Acquired:	23-Apr-25 00:54:32 CEST		
Date Processed:	23-Apr-25 11:49:37 CEST		

Auto-Scaled Chromatogram

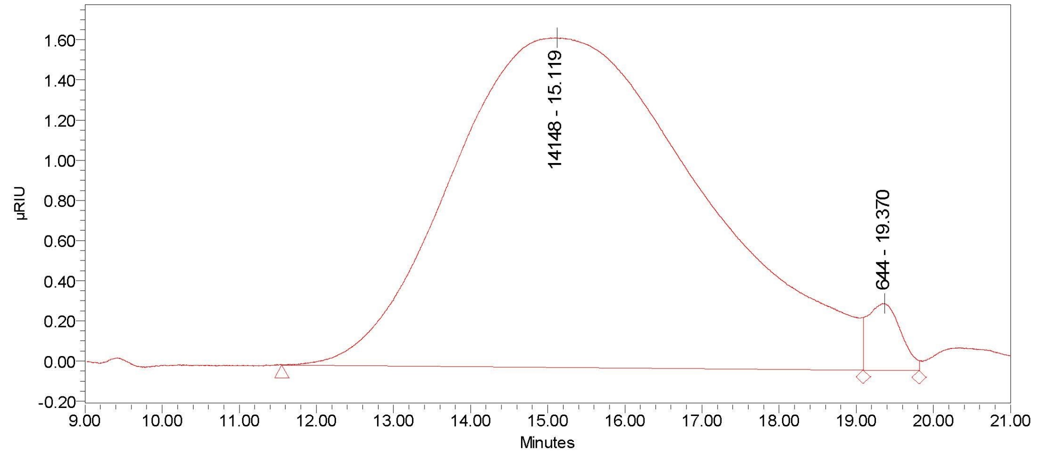

GPC Results

	SampleName	Mw	MP	Mn	Polydispersity	Area (µV*sec)	% Area
1	NK029	17008	14148	6285	2.705896	366200	97.37

Figure S87: *GPC trace of the oxyfunctionalised High M_w PS.*

PS Mid M_w

Analysis Report

SHIMADZU LabSolutions

<Sample Information>

Sample Name	: PS_24kDa_Reference_2	Sample ID	:
Data Filename	: PS_24kDa_Reference_2_MO-2_4282025_004.lcd	Method Filename	: DMF_Defa
Batch Filename	: MO-2.lcb		
Date Acquired	: 28-Apr-25 6:08:22 PM	Date Processed	: 28-Apr-25 6:33:23 PM

<Chromatogram>

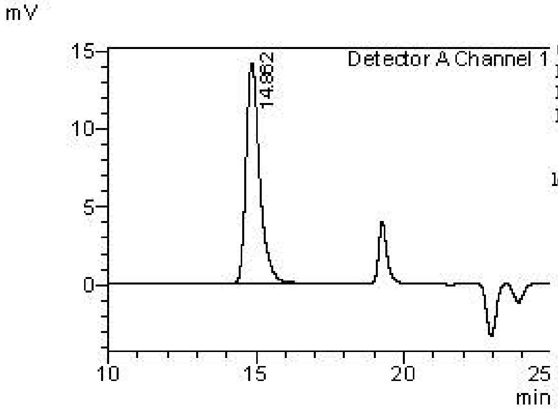

GPC Calibration Curve

Curve Fit Type :3rd Order
R^2=0.9999826
Dispersion=0.002519488
Function :f(x)=-0.001371635*X^3+0.0662550
-1357449*X+14.43673 (X=x-TLIMI)

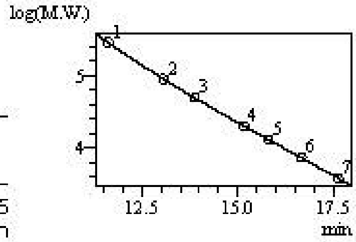

#	Molecular Weigh
1	288300
2	89050
3	49350
4	20020
5	13030
6	7350
7	3790

GPC Graph

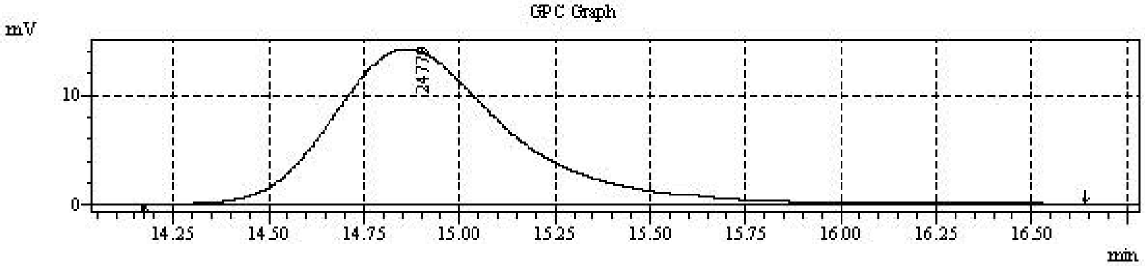

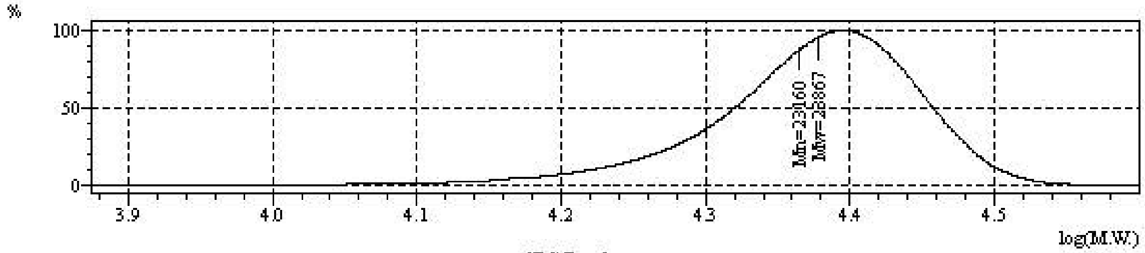

GPC Results

Peak#:1 (Detector A Channel 1)
[Peak Information]

Title	Time(min)	Molecular Weig	Height
Start	14.175	39891	81
Top	14.862	24779	14161
End	16.642	7480	119

Area : 446870

[Average Molecular Weight]

Number Average Molecular Weight(Mn)	23160
Weight Average Molecular Weight(Mw)	23867
Mw/Mn	1.03051

Figure S88: *GPC trace of the unfunctionalised Mid M_w PS.*

OxyPS Mid M_w

Analysis Report

SHIMADZU LabSolutions

<Sample Information>

Sample Name	: MOC066	Sample ID	:
Data Filename	: MOC066_MO-2_4282025_002.lcd	Method Filename	: DMF_Default_method.lcm
Batch Filename	: MO-2.lcb		
Date Acquired	: 28-Apr-25 5:17:24 PM	Date Processed	: 28-Apr-25 5:42:25 PM

<Chromatogram>

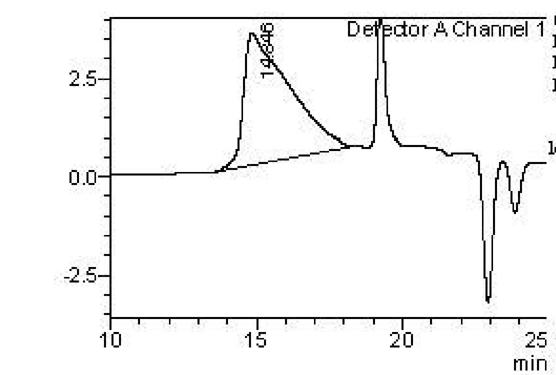

GPC Calibration Curve

Curve Fit Type : 3rd Order
R^2=0.9999826
Dispersion=0.002519488
Function :f(x)=-0.001371635*X^3+0.0662550 -1357449*X+14.43673 (X=x-TLIMI

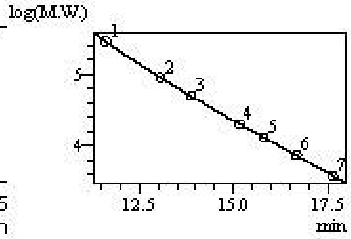

#	Molecular Weigh
1	288300
2	89050
3	49350
4	20020
5	13030
6	7350
7	3790

GPC Graph

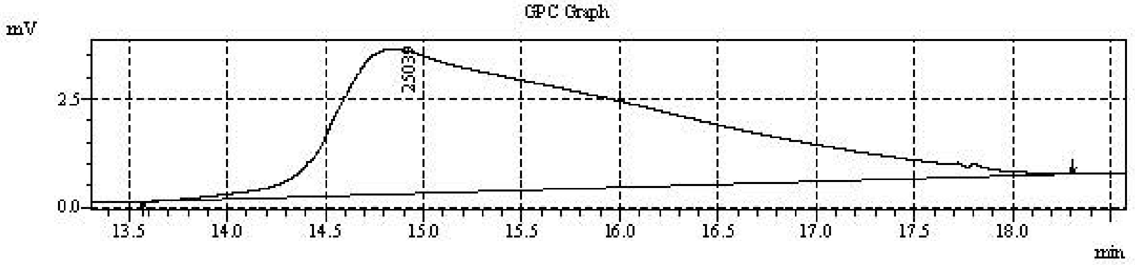

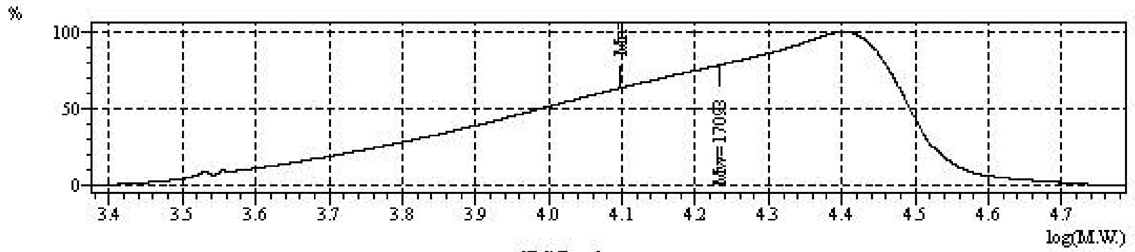

GPC Results

Peak#:1 (Detector A Channel 1)

[Peak Information]

Title	Time(min)	Molecular Weig	Height
Start	13.575	61317	123
Top	14.846	25039	3376
End	18.308	2371	769

Area : 365496

[Average Molecular Weight]

Number Average Molecular Weight(Mn)	12491
Weight Average Molecular Weight(Mw)	17093
Mw/Mn	1.36840

Figure S89: *GPC trace of the oxyfunctionalised Mid M_w PS.*

For the low molecular weight OPS entry and PS starting material, GPC was measured in THF using a refractive index detector. All samples were measured in duplo. The data for both starting material and oxidised polymer material is determined using the same calibration, shown below.

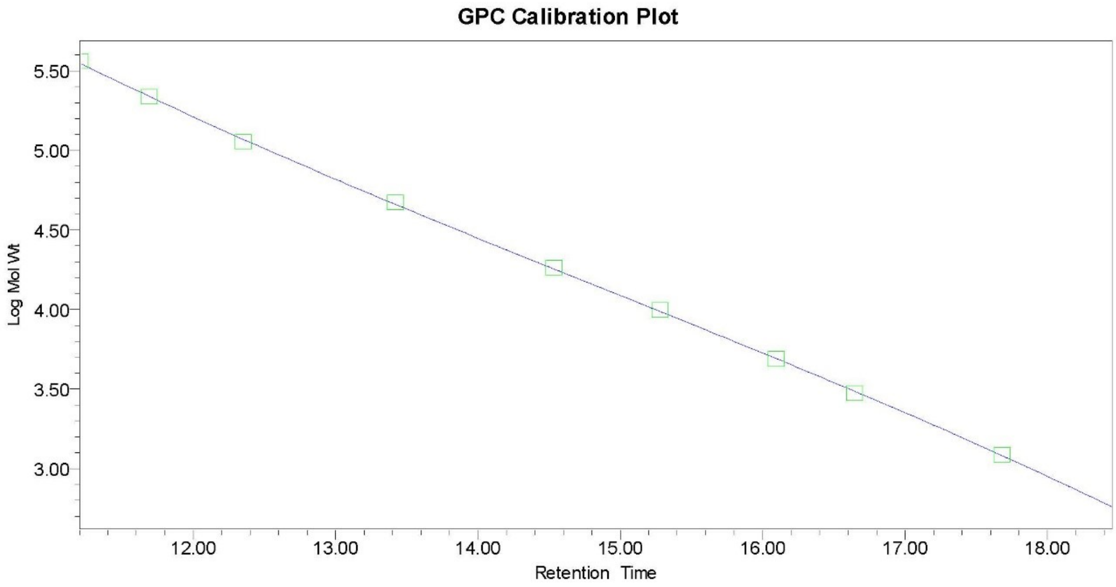

GPC Calibration Table

	Mol Wt (Daltons)	RT (min)	Calculated Weight (Daltons)	% Residual
1	364000	11.203	355366	2.430
2	113300	12.351	118385	-4.295
3	18340	14.534	18084	1.414
4	4900	16.093	4936	-0.735
5	1220	17.680	1213	0.556
6	217900	11.688	220272	-1.077
7	47190	13.420	46097	2.370
8	9960	15.277	9769	1.951
9	2980	16.647	3071	-2.948
10	580	18.452	577	0.571

Figure S90: *GPC calibration Plot for the measurements in THF.*

PS Low M_w

SAMPLE INFORMATION

Sample Name:	PS low Mw 1	Acquired By:	Waters
Sample Type:	Broad Unknown	Sample Set Name:	20250401 Maartje THF
Vial:	5	Acq. Method Set:	AL 100A@1 UV210_254_RI
Injection #:	1	Processing Method:	20250401 RI
Injection Volume:	50.00 ul	Channel Name:	W2414 RI
Run Time:	30.0 Minutes	Proc. Chnl. Descr.:	W2414 RI
Date Acquired:	01-Apr-25 18:35:18 CEST		
Date Processed:	02-Apr-25 16:22:58 CEST		

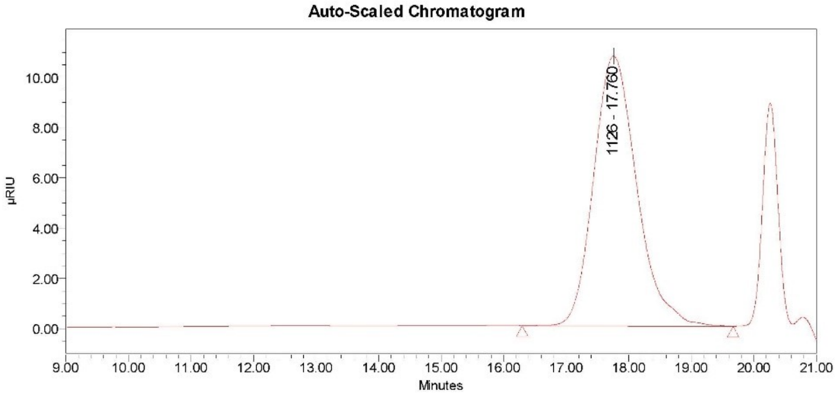

GPC Results

	SampleName	Mw	MP	Mn	Polydispersity	Area (µV*sec)	% Area
1	PS low Mw 1						
2	PS low Mw 1	1141	1126	1058	1.078165	514916	100.00

Figure S91: *GPC trace of the unfunctionalised Low M_w PS.*

OxyPS Low M_w

SAMPLE INFORMATION

Sample Name:	NK024 #1	Acquired By:	Waters
Sample Type:	Broad Unknown	Sample Set Name:	20250401 Maartje THF
Vial:	13	Acq. Method Set:	AL 100A@1 UV210_254_RI
Injection #:	1	Processing Method:	20250401 RI
Injection Volume:	50.00 ul	Channel Name:	W2414 RI
Run Time:	30.0 Minutes	Proc. Chnl. Descr.:	W2414 RI
Date Acquired:	01-Apr-25 22:43:24 CEST		
Date Processed:	02-Apr-25 16:22:00 CEST		

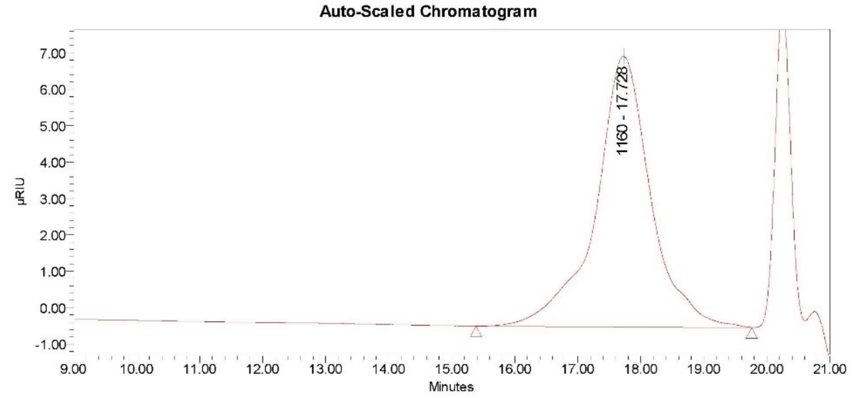

GPC Results

	SampleName	Mw	MP	Mn	Polydispersity	Area (µV*sec)	% Area
1	NK024 #1						
2	NK024 #1	1391	1160	1168	1.190548	452264	100.00

Figure S92: *GPC trace of the oxyfunctionalised Low M_w PS.*

For the OPBD8020 and OSBS entries as well as the PBD8020 and SBS starting material, GPC was measured in chloroform using a refractive index or 254 nm UV detector. All samples were measured in duplo. The data for both starting materials and oxidised polymer materials is determined using the same calibration, shown below.

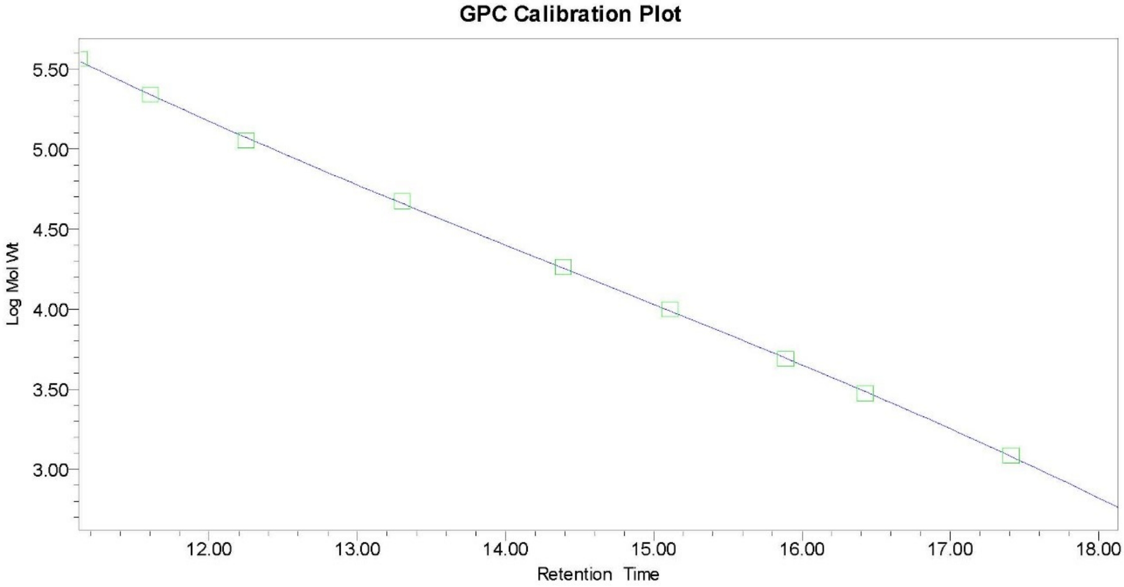

GPC Calibration Table

	Mol Wt (Daltons)	RT (min)	Calculated Weight (Daltons)	% Residual
1	364000	11.121	356342	2.149
2	113300	12.249	118594	-4.464
3	18340	14.389	18071	1.487
4	4900	15.889	4961	-1.220
5	1220	17.411	1207	1.055
6	217900	11.605	219352	-0.662
7	47190	13.302	46029	2.522
8	9960	15.109	9776	1.887
9	2980	16.426	3067	-2.839
10	580	18.131	578	0.327

Figure S93: *GPC calibration Plot for the measurements in chloroform.*

PBD8020

SAMPLE INFORMATION

Sample Name:	PBD8020 A	Acquired By:	Waters
Sample Type:	Broad Unknown	Sample Set Name:	20240710 Hanna
Vial:	3	Acq. Method Set:	AL 100A@1 UV210_254_RI
Injection #:	1	Processing Method:	Maartje_OCC_210nm 2peak
Injection Volume:	50.00 ul	Channel Name:	W2489 ChA
Run Time:	30.0 Minutes	Proc. Chnl. Descr.:	W2489 ChA 210nm
Date Acquired:	7/10/2024 11:36:06 AM CEST		
Date Processed:	7/11/2024 2:10:36 PM CEST		

Auto-Scaled Chromatogram

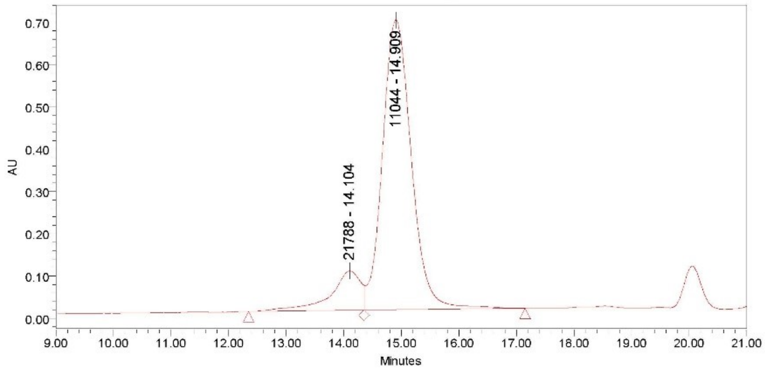

GPC Results

	SampleName	Mw	MP	Mn	Area (µV*sec)	% Area
1	PBD8020 A	26849	21788	24485	3311938	12.16
2	PBD8020 A	10834	11044	10201	23919826	87.84

Figure S94: *GPC trace of the unfunctionalised PBD8020.*

OPBD8020

SAMPLE INFORMATION

Sample Name:	MOC062_OPBD	Acquired By:	Waters
Sample Type:	Broad Unknown	Sample Set Name:	20250415_OCC_EVA_MAARTJE
Vial:	11	Acq. Method Set:	AI 100A@1 RI UV254
Injection #:	2	Processing Method:	20250405 MO RI
Injection Volume:	50.00 ul	Channel Name:	W2414 RI
Run Time:	30.0 Minutes	Proc. Chnl. Descr.:	W2414 RI
Date Acquired:	15-Apr-25 20:01:41 CEST		
Date Processed:	16-Apr-25 09:42:37 CEST		

Auto-Scaled Chromatogram

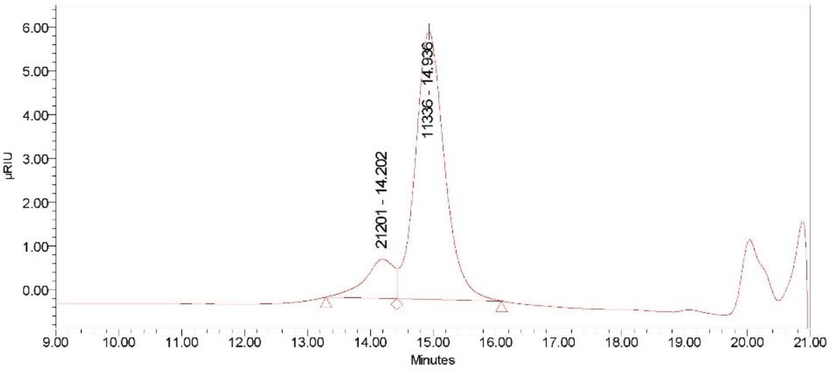

GPC Results

	SampleName	Mw	MP	Mn	Polydispersity	Area (µV*sec)	% Area
1	MOC062_OPBD	23638	21201	22732	1.039874	28890	12.79
2	MOC062_OPBD	11179	11336	10720	1.042780	196961	87.21

Figure S95: *GPC trace of the oxyfunctionalised PBD8020.*

OPBD8020

SAMPLE INFORMATION

Sample Name:	NK017_OPBD_2	Acquired By:	Waters
Sample Type:	Broad Unknown	Sample Set Name:	20250108 Hanna
Vial:	24	Acq. Method Set:	AI 100A@1 RI UV254
Injection #:	1	Processing Method:	20250108RI
Injection Volume:	50.00 ul	Channel Name:	W2414 RI
Run Time:	30.0 Minutes	Proc. Chnl. Descr.:	W2414 RI
Date Acquired:	1/9/2025 1:26:34 AM CET		
Date Processed:	1/9/2025 10:10:32 AM CET		

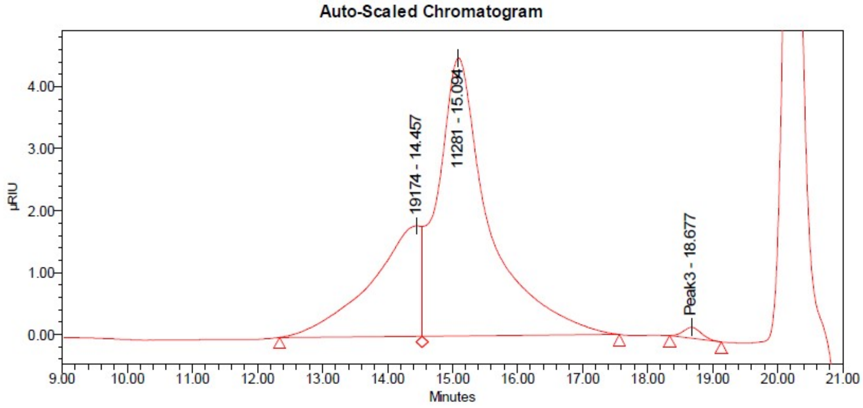

GPC Results

	SampleName	Mw	MP	Mn	Polydispersity	Area (µV*sec)	% Area
1	NK017_OPBD_2	31965	19174	27574	1.159222	94424	27.48
2	NK017_OPBD_2	10156	11281	8280	1.226580	245756	71.53

Figure S96: *GPC trace of the oxyfunctionalised PBD8020.*

OPBD8020

SAMPLE INFORMATION

Sample Name:	NK025_OPBD_2	Acquired By:	Waters
Sample Type:	Broad Unknown	Sample Set Name:	20250108 Hanna
Vial:	26	Acq. Method Set:	AI 100A@1 RI UV254
Injection #:	1	Processing Method:	20250108RI
Injection Volume:	50.00 ul	Channel Name:	W2414 RI
Run Time:	30.0 Minutes	Proc. Chnl. Descr.:	W2414 RI
Date Acquired:	1/9/2025 2:29:09 AM CET		
Date Processed:	1/9/2025 10:11:07 AM CET		

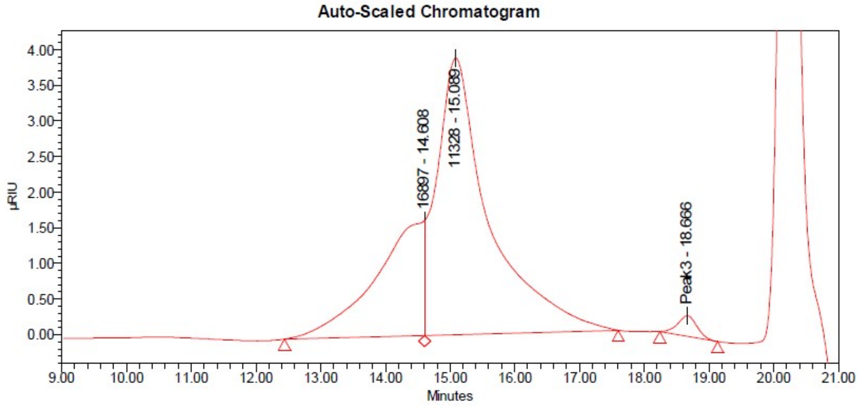

GPC Results

	SampleName	Mw	MP	Mn	Polydispersity	Area (µV*sec)	% Area
1	NK025_OPBD_2	29377	16897	25626	1.146392	83965	27.17
2	NK025_OPBD_2	9707	11328	7784	1.246968	218960	70.86

Figure S97: *GPC trace of the oxyfunctionalised PBD8020.*

SBS

SAMPLE INFORMATION

Sample Name:	PS_PBD_Copolymer	Acquired By:	Waters
Sample Type:	Broad Unknown	Sample Set Name:	20250425 MO
Vial:	3	Acq. Method Set:	AI 100A@1 RI UV254
Injection #:	2	Processing Method:	20250425UV
Injection Volume:	50.00 ul	Channel Name:	W2489 ChA
Run Time:	30.0 Minutes	Proc. Chnl. Descr.:	W2489 ChA 254nm
Date Acquired:	25-Apr-25 10:36:13 CEST		
Date Processed:	25-Apr-25 13:38:58 CEST		

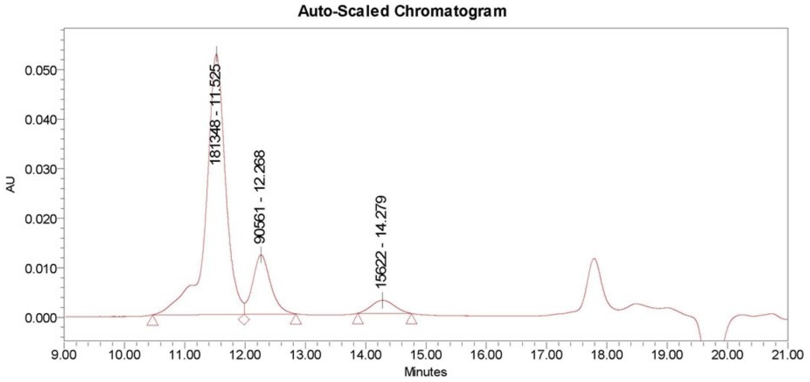

GPC Results

	SampleName	Mw	MP	Mn	Polydispersity	Area (µV*sec)	% Area
1	PS_PBD_Copolymer	190596	181348	183621	1.037986	1198793	79.17
2	PS_PBD_Copolymer	89486	90561	87947	1.017503	245887	16.24
3	PS_PBD_Copolymer	15537	15622	15227	1.020353	69602	4.60

Figure S98: *GPC trace of the unfunctionalised SBS.*

OSBS

SAMPLE INFORMATION

Sample Name:	MOC064	Acquired By:	Waters
Sample Type:	Broad Unknown	Sample Set Name:	20250425 MO
Vial:	4	Acq. Method Set:	AI 100A@1 RI UV254
Injection #:	2	Processing Method:	20250425UV
Injection Volume:	50.00 ul	Channel Name:	W2489 ChA
Run Time:	30.0 Minutes	Proc. Chnl. Descr.:	W2489 ChA 254nm
Date Acquired:	25-Apr-25 11:38:19 CEST		
Date Processed:	25-Apr-25 13:39:14 CEST		

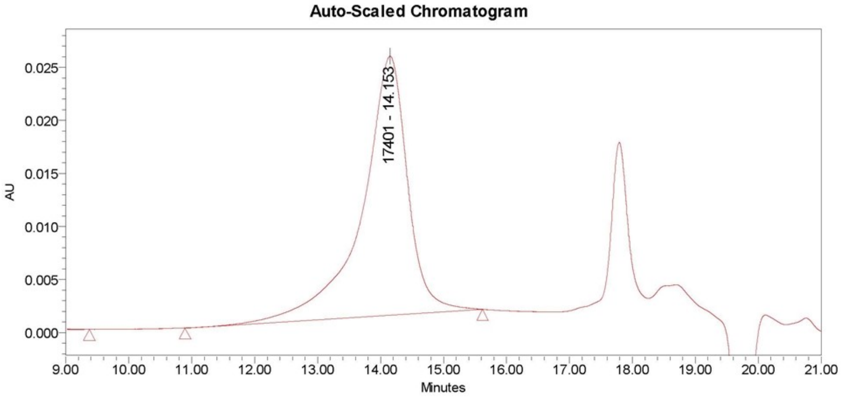

GPC Results

	SampleName	Mw	MP	Mn	Polydispersity	Area (µV*sec)	% Area
1	MOC064					46152	3.63
2	MOC064	22684	17401	18749	1.209874	1226441	96.37

Figure S99: *GPC trace of the oxyfunctionalised SBS.*

Oxidative cleavage of the fully hydroxylated PBD

SAMPLE INFORMATION

Sample Name:	MOC049#1	Acquired By:	Waters
Sample Type:	Broad Unknown	Sample Set Name:	20250402 Maartje CHCl3 Frances
Vial:	3	Acq. Method Set:	Al 100A@1 RI UV254
Injection #:	1	Processing Method:	20250402 UV
Injection Volume:	50.00 ul	Channel Name:	W2489 ChA
Run Time:	30.0 Minutes	Proc. Chnl. Descr.:	W2489 ChA 254nm
Date Acquired:	02-Apr-25 13:27:45 CEST		
Date Processed:	09-Apr-25 10:15:47 CEST		

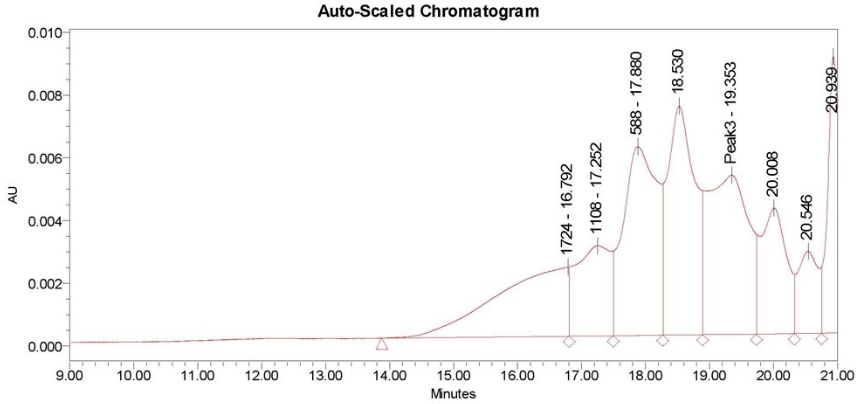

GPC Results

	SampleName	Mw	MP	Mn	Polydispersity	Area (µV*sec)	% Area
1	MOC049#1	4004	1724	3127	1.280431	171711	13.27
2	MOC049#1		1108			107441	8.30
3	MOC049#1	689	588	680	1.013028	221576	17.12

Figure S100: *GPC trace of the oxidatively cleaved fully hydroxylated PBD.*

References

(1) Wieghardt, K.; Bossek, U.; Ventur, D.; Weiss, J. Assembly and Structural Characterization of Binuclear μ-Oxo-di-μ-acetato Bridged Complexes of Manganese(III). Analogues of the Di-Iron(III) Centre in Hemerythrin. *J. Chem. Soc. Chem. Comun.* **1985**, 347–349.

(2) Hage, R.; Gunnewegh, E. A.; Niël, J.; Tjan, F. S. B.; Weyhermüller, T.; Wieghardt, K. NMR Spectra of Dinuclear Manganese and Iron Compounds Containing 1,4,7-Triazacyclononane and 1,4,7-Trimethyl-1,4,7-Triazacyclononane. *Inorganica Chim. Acta* **1998**, *268* (1), 43–48.

(3) Lindsay Smith, J. R.; Shul'pin, G. B. Efficient Stereoselective Oxygenation of Alkanes by Peroxyacetic Acid or Hydrogen Peroxide and Acetic Acid Catalysed by a Manganese(IV) 1,4,7-Trimethyl-1,4,7-Triazacyclononane Complex. *Tetrahedron Lett.* **1998**, *39* (27), 4909–4912.
